# In vitro and in vivo antibacterial activities and phytochemical screening of 80% methanol extract from *Ehretia cymosa* leaves

**DOI:** 10.1371/journal.pone.0354982

**Published:** 2026-07-31

**Authors:** Eshetu Mekonnen, Dumessa Edessa, Fitsum Weldegebreal, Abenezer Aklog, Tigist Gashaw

**Affiliations:** 1 Besidemo Hospital, Pharmacy Department, Harar, Ethiopia; 2 Clinical Pharmacy Department, School of Pharmacy, College of Health and Medical Sciences, Haramaya University, Harar, Ethiopia; 3 School of Medical Laboratory Sciences, College of Health and Medical Sciences, Haramaya University, Ethiopia; 4 Laboratory Bacteriology Research, Department of Diagnostic Sciences, Faculty of Medicine and Health Sciences, Ghent University, Ghent, Belgium; 5 Pharmacology Department, School of Pharmacy, College of Health and Medical Sciences, Haramaya University, Harar, Ethiopia; Universidade Federal do Para, BRAZIL

## Abstract

Antibiotic resistance has emerged as one of the most urgent global health threats, undermining the effective treatment of bacterial infections. In response, scientific interest is increasingly focused on identifying natural and effective antimicrobial agents derived from medicinal plants. In Ethiopia, *Ehretia cymosa* (*E. cymosa*) is traditionally used to treat wound infections, fever, gastric ulcers, dysentery, and toothache. However, there is limited scientific evidence to support these traditional claims. Hence, the present study aimed to evaluate the in vitro and in vivo antibacterial activities and to screen the phytochemical profile of the 80% methanol extract of *E. cymosa* leaves. The air-dried and powdered leaves of *E. cymosa* were extracted using cold maceration with 80% methanol. The antibacterial activity of the crude extract was tested using the disk diffusion method against selected bacterial pathogens commonly associated with infections. An in vivo model of burn followed by infection was established in mice. Qualitative phytochemical screening was also performed. One-way analysis of variance followed by Tukey’s post hoc multiple tests was used to compare the means of all parameters. The leaves of *E. cymosa* demonstrated significant antibacterial activity (p < 0.001) against the tested bacterial strains in a dose-dependent manner compared with the control. The minimum inhibitory concentration ranged from 6.25 to 75 mg/mL, while the minimum bactericidal concentration against *P. aeruginosa* and *E. coli* was 200 mg/mL. In the in vivo model, the extract resulted in faster wound contraction and a shorter epithelialization period against *S. aureus* than against *P. aeruginosa*. The plant leaf is also rich in flavonoids, terpenoids, and tannins. The 80% methanol extract of *E. cymosa* leaves exhibited antibacterial activity in vitro and in vivo, which corroborates the traditional use of the leaves against infectious diseases. Further studies involving the isolation and characterization of the active compounds are recommended.

## Introduction

The discovery, development, and therapeutic application of antibiotics in the nineteenth century have significantly decreased the public health risks posed by bacterial infections [1,2]. However, the misuse and overuse of these drugs have led to a troubling rise in bacterial resistance to current chemotherapeutic agents. Additionally, antibiotics can sometimes cause adverse effects, including allergic reactions, immunosuppression, and hypersensitivity [3,4]. These challenges highlight the urgent need to intensify efforts toward developing new antibacterial agents that are effective against resistant pathogenic microorganisms [5].

One promising approach to combat the growing issue of antibiotic resistance is the systematic extraction and screening of bioactive compounds from traditional medicinal plants. This strategy aims to discover new, effective substances with potential antimicrobial properties [6,7]. The demand for plant-based treatments is growing in both developed and developing countries, driven by the traditional practices that these remedies are natural, safe, affordable, and associated with fewer side effects [8,9].

Medicinal plants have shown antibacterial activity against various pathogens responsible for infections, especially wounds, including *Staphylococcus aureus* (*S. aureus),* Pseudomonas *aeruginosa (P. aeruginosa),* and *Streptococcus* species [10,11]. An astounding array of antibacterial plants has been found worldwide, where larger populations reside in lower- and middle-income countries that are dependent on traditional plants; *Achiella millefolium, Aloe vera, Althaea officinalis, Calendula officinalis, Matricaria chamomilla, Curcuma longa, Eucalyptus, Jojoba, plantain, pine, green tea, pomegranate, Inula, Arctium lappa*, and root extracts of *Astragalus propinquus* and *Rehmannia glutinosa***;** and the roots of Ampelopsis japonica, *Andrographis paniculata, Angelica sinensis, Blumea balsamifera, Boswellia sacra, Caesalpinia sappan, Calendula officinalis, Camellia sinensis, Carthamus tinctorius, Celosia argentea, Centella asiatica, Cinnamomum cassia, and Commiphora myrrha* [10,12–14].

Ethiopia is home to a diverse array of plant species, about 12% of which are indigenous, making the country a valuable source of medicinal plants with potential health benefits. *Ehretia cymosa* (*E. cymosa*) is a native plant widely distributed across Ethiopia. It is locally known as “Game” in Amharic and “Hulaagaa, Ulaagaa, Garmi” in Afan Oromo. It is traditionally used to treat gastrointestinal problems, bone fractures, skin infections, paralysis, and epilepsy in different regions of Africa [15]. Experimental studies have reported that crude extracts and solvent fractions of *E. cymosa* exhibit biological activities, including antioxidant, antihyperglycemic, analgesic, anti-inflammatory, and antimicrobial effects [16–18]. In eastern Ethiopia, the plant leaves are prepared as a poultice, a soft, moist mass of plant material applied directly to wounds. To make a poultice, fresh leaves are mashed with cold water and then reduced in size, or dried leaves are boiled in water and cooled.

Although preliminary in vitro studies have demonstrated antibacterial activity, further studies are needed to support the broader traditional use of this plant and to verify that the therapeutic effects observed in vitro translate to living systems. Experiments conducted within a living organism are critical, often mandatory, steps in scientific, biomedical, and pharmaceutical research that laboratory tests cannot fully replace [19,20]. Therefore, we tested additional strains, quantified the minimum bactericidal concentration (MBC), and employed in vivo models. Hence, the present study aimed to evaluate the in vitro and in vivo antibacterial activities and to screen the phytochemical profile of the 80% methanol extract of *E. cymosa* leaves.

## Materials and methods

### Chemicals

All solvents used for the extraction process were of laboratory (analytical) grade. Drugs and chemicals, including absolute methanol (LOBA Chemie Pvt. Ltd., India) and distilled water, were used to extract the plant material. A simple ointment base was prepared using the following ingredients: wool fat, hard paraffin, and cecostearyl alcohol (all from BDH Chemicals Ltd., England), along with white soft paraffin (Anonchem Ltd., China). The additional chemicals used included Mueller-Hinton agar, normal saline, and Nitrofurazone ointment USP 0.2% (Galentic Pharm, Pvt. Ltd. Co., India), ketamine hydrochloride injection USP (Neon Laboratories Ltd., India), ciprofloxacin 5 µg standard antibiotic disc (OXOID LIMITED, Basingstoke, Hampshire, England), and dimethyl sulfoxide (DMSO)

### Collection, authentication, and extraction of *E. cymosa* leaves

The plant material was collected from eastern Ethiopia, identified, and authenticated by a biologist. A voucher specimen, labeled HUHE0000002255 (023551), was deposited in the Herbarium of the Biology Department, Faculty of Natural and Computational Science at Haramaya University.

Fresh *E. cymosa* leaves were collected and thoroughly washed with tap water to remove surface dirt. The cleaned leaves were shade-dried and ground into a fine powder using a mortar and pestle. Methanol (80% (v/v)) was selected as the solvent because of its high polarity, efficiency, compatibility with analytical techniques, and proven effectiveness in previous studies. Although some solvents may offer higher potency, 80% methanol provides a balanced approach in terms of safety, solubility, and extraction yield.

A total of 600 grams of powdered plant material was weighed using an electronic digital balance and soaked in 300 mL of 80% methanol in Erlenmeyer conical flasks. Extraction was performed via cold maceration. The flasks were placed on mini-orbital shakers set at 120 revolutions per minute (rpm) and agitated periodically for 72 hours. The mixture was first filtered through a nylon cloth, and the residue was macerated twice more to maximize the extraction yield. The filtrate was filtered again using Whatman filter paper (No. 1) under a pressure-suction system. The total filtrate was concentrated via a low-pressure rotary evaporator set at 121 mbar, 45 rpm, and 40°C. The resulting concentrate was stored in a deep freezer and subsequently freeze-dried via a lyophilizer at −20°C under vacuum pressure (200 mBar), yielding a brownish crude extract.

### Microbial organisms

Standard strains (American Type Culture Collections (ATCC)) of the gram-positive bacterial species *Staphylococcus aureus (S. aureus) (ATCC 25923) and Streptococcus pyogenes (S. pyogenes) (NCTC 12696)* and the gram-negative bacteria *Escherichia coli (E. coli) (ATCC 25922),* Pseudomonas *aeruginosa (*P. *aeruginosa) (ATCC27853),* and Klebsiella *pneumoniae (*K. *pneumoniae)* were obtained from Eastern Hararghe Health Research at Haramaya University, Harar.

### Preparation of experimental animals

Healthy Swiss albino adult mice of both sexes weighing 20–30 g and aged 6–8 weeks were obtained from the National Veterinary Institute (NVI), Bishoftu, Ethiopia, and used throughout the experiment. The animals were housed in plastic cages under standard conditions, with a 12-hour light/dark cycle and ad libitum access to standard food and water. The samples were allowed to acclimate to laboratory conditions for one week before any experiments were initiated. Throughout the study, the animals were handled in accordance with international guidelines for the use and care of experimental animals [20,21]. At the conclusion of the experiment, the animals were euthanized with the recommended dose of ketamine.

### Dosing in the mouse model

The concentrations were selected based on a combination of factors, including prior toxicity studies that established the extract’s safety up to 2000 mg/kg and the need to assess dose-dependent efficacy within a safe range. When calculating a dose for in vivo delivery, we aimed to use a dose that was greater than the minimum inhibitory concentration (MIC) or minimum bactericidal concentration (MBC) determined in vitro. Because in vitro experiments are performed under controlled settings, they do not account for the wide range of physiological situations encountered in the animal body [22]. The in vivo dose must account for skin permeability, protein binding, and local metabolism to ensure that the medication reaches the infection site at concentrations higher than the MBC or maintains levels above the MIC while taking pharmacokinetic/pharmacodynamic features into account. According to research, the target skin layer should start at 4x to 10x MIC [23].

### *Ehretia cymosa* leaf ointment formulation

A simple ointment base was prepared according to the British Pharmacopoeia [24]. The indicated ointment preparations, each 200 g, were prepared in two strengths (5% w/w and 10% w/w), and a simple ointment without an active substance (as a control) was prepared via a reduced formula (Table 1). To prepare the ointment, a calculated amount of hard paraffin and cetostearyl alcohol was combined and heated in a beaker in a water bath. In a separate beaker, the wool, fat, and white soft paraffin mixture was heated while stirring to ensure homogeneity. After removal from the water bath, the two mixtures were combined and mixed until cold.

**Table 1:** Master and reduced formulas used for formulating a simple ointment.

Ingredients	Master Formula	Reduced Formula
Wool fat	50 g	10 g
Hard paraffin	50 g	10 g
Ceto-stearyl alcohol	50 g	10 g
White soft Paraffin	850 g	170 g
QS	1000 g	200 g

**Abbreviations:** Q.S. sufficient quantity

A medicated ointment with a homogeneous consistency and smooth texture was produced. To prepare 5% (w/w) ointment, 10 g of extract was weighed and combined with 190 g of simple ointment base. Similarly, to prepare 10% (w/w) ointment, 20 g of extract was mixed with 180 g of ointment base. This preparation was performed at five-day intervals until all the experiments were completed. A nonmedicated ointment base was used as a negative control.

### In vitro antibacterial activity testing

The in vitro antibacterial activity of the crude extract was evaluated against five bacterial strains: *S. aureus, S. pyogenes*, *E. coli,* P. *aeruginosa,* and K. *pneumoniae.* The selection of these strains was based on the common bacteria known in skin infections and wounds, which was the traditional claim in the study community [25–30].

### Agar disc diffusion

The agar disc diffusion method was used to determine the antibacterial activity (zone of inhibition) of the *E. cymosa* extract [31,32]*.* The five bacterial strains were activated on their selective media, MacConkey agar for K. pneumoniae and mannitol‒salt agar for S. aureus, and incubated at 37°C for 24 hours**.** Muller‒Hinton agar (MHA) was prepared according to the manufacturer’s recommendations (39 g diluted in 1,000 mL of distilled water). The powdered medium was added to a flask containing the measured volume of distilled water, and the flask was set on a hot plate, occasionally shaken until boiling.

The media were sterilized in an autoclave at 121°C for 15 minutes. The medium was cooled to 45–50°C in a water bath. For fastidious *Streptococcus* species (*S. pyogenes*) that require enriched media, 5% sterile sheep blood was added to the MHA medium. In microbiology and research, sheep blood cannot be heat-sterilized, as high temperatures will destroy (hemolyze) red blood cells and coagulate proteins. Instead, it must be collected using strict aseptic techniques. Blood will be collected from a sheep in good health, without any antibiotic treatment or vaccine in the past 30 days. A trained veterinarian will collect the blood following aseptic technique into sterile EDTA tubes [33]. A total of 20 mL of medium was aseptically poured into prelabeled sterile Petri dishes (90 mm) and allowed to solidify [34–36]. Using a sterile inoculating loop, a few bacterial colonies—each strain from the agar plate—were transferred to nutritional broth until the turbidity reached 10^8^ cells per milliliter of the McFarland 0.5 turbidity standard [37]. The culture was then evenly dispersed over MHA using sterile cotton brushes and aseptic settings in a safety cabinet with Petri dishes rotated 60° to ensure consistent bacterial growth. Finally, the medium was allowed to cure at room temperature for five minutes.

The external surface of each plate was divided into five parts and labeled with a permanent marker; each consisted of five paper discs: three discs containing extracts at different concentrations, one for the positive control, and the remaining for the negative control. Paper discs were punched from a sheet of absorbent filter paper (Whatman No. 1; 6 mm in diameter) via an ordinary office two-hole puncher. The discs were dispensed into batches in screw-capped bottles and sterilized at 121°C for 1 hr.

A test sample was prepared at three concentrations, 300 mg/mL, 200 mg/mL, and 100 mg/mL, using 10% DMSO as the solvent. Previous research at 25 mg/mL revealed initial antibacterial potential; however, the present concentrations were used to determine whether higher doses yield stronger antibacterial effects. Testing across this range allows us to capture dose‒response relationships that could identify an optimal therapeutic concentration. A 10% DMSO solution was used as a solvent due to its effective solubilizing properties and low cytotoxicity, ensuring reliable in vitro results.

The sterilized filter paper discs were soaked in beakers containing different concentrations of solvent extracts and the negative control (DMSO). The discs were then removed with sterilized forceps, air-dried, and placed on plates with test organisms. A 5-µg ciprofloxacin disc was used as a positive control. Ciprofloxacin is selected as a standard in antibacterial research, primarily because of its broad spectrum of activity, well-understood bactericidal mechanism of action, established pharmacokinetic profile, and frequent use as a reference standard in official pharmacopoeias for quality control [38–40].

The plates were left at room temperature for 2 hours to allow diffusion of the test samples and then incubated at 37°C for 24 hours. After incubation, the plates were examined for zones of inhibition (areas without bacterial growth). Inhibition zones were measured in millimeters (mm) via a ruler, and the tests were performed in triplicate [34,35].

In agar diffusion experiments, free diffusion is strictly dependent on the polarities of the medium, extract, and controls. Because agar is a highly polar, watery matrix, polar molecules diffuse freely, but non-polar or highly lipophilic compounds do not, resulting in inconsistent or false-negative results [41]. Agar is an aqueous (water-based) and hydrophilic gel, which makes it extremely polar. Our plant extract is polar (extracted with hydro methanol) and will diffuse freely, resulting in a uniform concentration gradient [33,42]. Positive Control: standard antibiotic (ciprofloxacin) is water-soluble (polar), allowing it to diffuse freely and radially across the agar [43]. Negative Control: the solvent used to dissolve the extract (DMSO) has a polarity similar to that of the extract and agar, preventing precipitation and ensuring assay validity [44]. For accurate comparisons in agar-diffusion tests, the extract, solvent, controls, and agar must all have equivalent polarity.

### Determination of the minimum inhibitory concentration

For the concentration that produced a zone of inhibition greater than or equal to 7 mm for the tested organism, the MIC was determined via the broth macrodilution method [34]. The extract, which showed activity at 300 mg/mL, was further diluted to a 1:2 ratio to obtain 150 mg/mL, 125 mg/mL, and 75 mg/mL (1:4) extracts. The concentration that showed activity at 100 mg/mL was diluted to 50 mg/mL (1:2), 25 mg/mL (1:4), 12.5 mg/mL (1:8), and 6.25 mg/mL (1:16) to explore lower concentrations to refine the MIC.

Broth media were prepared according to the manufacturer’s recommendation. Inoculum preparation and standardization were performed aseptically [34]. Each extra concentration was diluted 1:2 in the broth medium, and 1 mL of each mixture was added to a standard test tube. The standard inocula were diluted in broth media (1:150) [45,46]. One milliliter of the diluent containing different concentrations of the extract was added to each tube. A tube without any test sample was used as a control for bacterial growth. The tubes were covered and incubated at 37°C for 24 hours. Broth Dilution (Liquid Broth) is widely considered the gold standard reference method. The MIC is the lowest concentration that prevents visible bacterial growth, measured by the absence of cloudiness or “turbidity” [34,47,48].

### Determination of the minimum bactericidal concentration

MBC was determined by subculturing the extract at a concentration of at least the MIC. The contents of the MIC test solutions were streaked on an agar plate via sterile cotton swabs and incubated at 37°C for 24 hours. The lowest concentration that yielded no single bacterial colony was taken as the MBC [48–50]

### In vivo antibacterial activity testing in the infected wound model

In vivo antibacterial activity was tested using *P. aeruginosa* and *S. aureus.* The burn model followed by the infection model is crucial for understanding and improving burn wound treatment because it closely replicates the clinical scenario of burn injuries, which are highly susceptible to infection [51].

### Animal grouping

The animals were randomly assigned to two experimental arms and grouped into five groups in each arm. Each group was then randomly assigned to five mice, assuming that the mean difference between the groups was more than the hypothesized value. Since there is no study on our topic of interest, we are unable to use previous effect sizes. Accordingly, the degree of freedom (E) was considered. In this method, a value “E” is measured, which is simply the degree of freedom of analysis of variance (ANOVA). The value of E should be between 10 and 20. If E is less than 10, adding more animals will increase the chance of obtaining a more significant result. But if E is more than 20, adding more animals will not increase the chance of obtaining significant results. Although this method is based on ANOVA, it applies to all animal experiments. Any sample size that keeps E between 10 and 20 should be considered adequate. E can be measured via the following formula: E = total number of animals − total number of groups. Hence, E = (5 mice × 5 groups) – 5 groups = 20 [52].

### Antibacterial testing

There were five interventions with two infecting bacterial strains: the control (no treatment), negative control (receiving placebo ointment), positive control (0.2% nitrofurazone ointment), 5% *E. cymosa* ointment-treated, and 10% *E. cymosa* ointment-treated groups. Nitrofurazone is selected as a standard in experimental research, primarily because of its well-characterized, broad-spectrum antibiotic activity and known toxicological profile, which provides a reliable and consistent baseline for comparison with new or related compounds [53].

The mice were burned and infected with *P. aeruginosa* and *S. aureus* at a sublethal dose of 1 × 10^7^ colony-forming units (CFUs)/mL/mouse in each intervention category. First, the mice were anesthetized, shaved, and cleaned. A metal mold with selected dimensions was used to provide the desired burn size in an animal of known weight. The upper limit of burn size permitted by this approach is approximately 30% of the TBSA, which is calculated via Meeh’s formula [54,55], and the mean burn area for each group is 28.26 mm^2^. To make the wound, the metal mold was heated via a Bunsen burner. The temperature of the heated metal was 100°C. The metal mold was then cautiously pressed on the mouse’s back to avoid skin damage. The metal mold was removed, and cold water was sprayed for cooling after 10 s, based on the literature recommendations [56–59].

To prevent hypothermia and shock, the mice were placed on an electric heating pad following the burn. Such an injury is not fatal, but it results in a third-degree (full-thickness) burn. Fluid replacement therapy was initiated with a subcutaneous injection of 0.8 mL of 0.9% sodium chloride solution. Mice were infected with *P. aeruginosa* and *S. aureus* at sublethal doses of

1 × 10^7^ CFU/mL/mouse from a preprepared McFarland 0.5 turbidity standard, according to their groups. After recovery, the mice were returned to their cages and fed regularly. Starting 72 hours after wounding (day 3), the animals were treated with the corresponding ointment as specified in the grouping and dosing section, except for the last group, which was left untreated and served as a benchmark (untreated negative control). Since untreated wounds were exposed to open air, each animal was housed in a separate cage. The lesion healing progress, normal skin structure restoration, and hair growth on skin repaired by topical wound therapy were documented and calculated using Meeh’s formula as follows:

$TBSA=kW^{\text{2}/\text{3}}$, where TBSA is the total body surface area, k is Meeh’s constant (9.83), and W is the weight of the mice in g [60].

### Data collection procedures

We employed single-blinding (masking) during the experiment and outcome assessment to reduce subjective bias and increase validity. The groups were coded, and the intervention information was masked [61,62].

### Preliminary phytochemical screening

Qualitative screening for the presence of secondary metabolites in the 80% methanol extract of *E. cymosa* leaves was performed via standard tests described previously [18,63–65]. The presence of alkaloids, saponins, flavonoids, terpenoids, phenols, glycosides, and tannins was tested following the procedures discussed below [18,65].

#### Test for saponins (foam test).

From the extract, 0.25 g of the sample was taken and dissolved in 5 mL of distilled water. The mixture was subsequently shaken vigorously and observed for stable, persistent froth. The formation of froth indicates the presence of saponins [65].

#### Test for Alkaloids.

A total of 0.5 g of the extract was stirred with 1% HCl (10 mL) in a water bath for 5 min and then filtered. The filtrate was divided into three equal parts. To one portion of the filtrate, 1 mL of Wagner’s reagent (1 g of iodine + 3 g of potassium iodide in 50 mL of distilled water) was added. The formation of a reddish-brown precipitate indicated the presence of alkaloids [65–67].

#### Test for Tannins.

A total of 0.25 g of the extract was boiled in 10 mL of water in a test tube and filtered. To the filtrate, a few drops of 0.1% FeCl_3_ (ferric chloride) were added, resulting in a brownish-green or blue‒black color, which confirms the presence of tannins [65,66].

#### Test for Terpenoids.

A total of 0.25 g of the extract was mixed with 2 mL of chloroform, and 3 mL of concentrated H_2_SO_4_ was carefully added to form a layer. A reddish-brown color of the interface was formed, indicating the presence of terpenoids [64].

#### Test for flavonoids.

Ten milliliters of ethyl acetate was added to 0.2 g of the extract, which was subsequently heated for 3 min in a water bath. The mixture was filtered, and the filtrate was then mixed with 1 mL of dilute ammonia solution. The formation of an intense yellow color highlights the presence of flavonoids [18,63].

#### Test for steroids.

A total of 0.25 g of the extract was dissolved in 0.25 mL of dichloromethane to produce a dilute solution. To this mixture, 0.25 mL of acetic anhydride was added, followed by three drops of concentrated sulfuric acid. The formation of a blue‒green color indicates the presence of steroids [64].

#### Test for glycoside.

From the extract, 0.25 g was diluted with 5 mL of distilled water, and 2 mL of glacial acetic acid containing one drop of ferric chloride solution was added. One milliliter of concentrated sulfuric acid was then added to the solution. The formation of a brown ring at the interface indicates the presence of glycosides [64,65].

#### Test for phenols.

A 10 mg sample from the extract was dissolved in 1 mL of water. Half a milliliter of 5% ferric chloride solution was added to the solution, and the development of a deep blue or black color was taken as an indicator of the presence of phenols [63–66].

### Statistical analysis

Statistical analysis was performed using the software programs for social science students (SPSS) version 27. Data are reported as mean ± SEM. The means of the groups’ parameters were compared using one-way ANOVA and Tukey’s post hoc multiple testing. P-values < 0.05 were considered statistically significant.

#### Ethical clearance.

Before the study, ethical approval was obtained from Haramaya University, specifically from the College of Veterinary Medicine, Animal Ethical Review Committee (Reference number: CVM/412/2024). Moreover, this study adhered to the guidelines outlined in the “Guide for the Care and Use of Laboratory Animals” by the National Research Council of the National Academies [68]. All surgeries were performed under anesthesia, and all efforts were made to minimize suffering.

## Results

### Yields of extraction

A total of 600 g of the coarse leaf powder of *E. cymosa was* extracted with 80% methanol, and 104.17 g of dark brown paste was obtained. The yield was calculated to be 17.36% (w/w).

### In vitro antibacterial activity

#### Bacterial growth inhibition.

Using the agar disc diffusion method, the 80% methanol extract of *E. cymosa* leaves demonstrated statistically significant (p < 0.001) antibacterial activity against all standard bacterial strains compared with the negative control (DMSO) in a concentration-dependent manner. The zones of inhibition diameter ranged from 7.17 mm (*S. pyogenes*) to 22.5 mm (*S. aureus)*. The extract had the greatest growth inhibitory effect on the gram-positive bacterium *S. aureus,* followed by the gram-negative bacteria *P. aeruginosa, E. coli, and K. pneumoniae*. Among the tested strains, *S. pyogenes* was the least sensitive to the extract across all concentrations. The positive control (ciprofloxacin) consistently showed significant differences (p < 0.05 to p < 0.001) compared with all extract concentrations and the negative control across all bacterial strains (Table 2).

**Table 2:** Zone of inhibition of the 80% methanol extracts of *E. cymosa* leaves at various concentrations and the standard ciprofloxacin.

Interventions	Zone of inhibition (mm) mean ± SEM				
	Gram-positive bacteria		Gram–negative bacteria		
	*S. aureus*	*S. pyogens*	*P. aeruginosa*	*E. coli*	*K. pneumoniae*
5 µg/mL (Cip)^**a**^	26.33 ± 0.88^cd*be***^	25.00 ± 0.33^bcde***^	25.00 ± 1.00^d**bce***^	35.00 ± 2.08^bcd**e***^	21.00 ± 0.58^d**bce***^
100 mg/mL^**b**^	17 ± 0.58 ^e***^	7.17 ± 0.17^e***^	11.67 ± 0.67^e***^	11 ± 2.08^e**^	8.67 ± 0.73^e***^
200 mg/mL**^c^**	21.17 ± 1.26^b*e***^	9.00 ± 0.58^b*e***^	15.00 ± 0.58^b*e***^	14.00 ± 3.00^e**^	11.17 ± 0.93^e***^
300 mg/mL^**d**^	22.5 ± 0.87^b**e***^	11.33 ± 0.88^b**e***^	16.67 ± 0.67^b** e***^	15.67 ± 3.18^e**^	12.33 ± 0.88^b*e***^
DMSO^**e**^	NA	NA	NA	NA	NA

Note: Values are expressed as the means ± SEM (n = 3). **Abbreviations:** Cip, ciprofloxacin; DMSO, dimethyl sulfide; NA, no activity/inhibition at the tested dose(s); *a* compared with the positive control (ciprofloxacin); *b* compared with 100 mg/ml; *c* compared with 200 mg/ml; *d* compared with 300 mg/ml; and e compared with the negative control (DMSO). *P < 0.05; **P < 0.01; ***P <0.001.

### Minimum inhibitory concentration and minimum bactericidal concentration

To quantitatively assess the antibacterial activity of the 80% methanol extract of *E. cymosa* leaves, the MIC and MBC cutoff values were determined for each concentration. These values were obtained for bacterial strains whose growth was inhibited in the agar disc diffusion assay. The lowest MIC (6.25 mg/mL) was recorded for *P. aeruginosa*. MBC (200 mg/mL) was observed against both *P. aeruginosa* and *E. coli.* However, no MBC values were observed for *S. aureus, K. pneumoniae,* or *S. pyogenes* within the tested concentration range (Table 3).

**Table 3:** MICs and MBCs of the 80% methanol extract of the leaves of *E. cymosa.*

Bacterial strains		MICs (mg/mL)	MBCs (mg/mL)
Gram +Ve	*S. aureus*	50.00 ± 0.00	–
	*S. pyogen*	75.00 ± 0.00	–
Gram –Ve	*P. aeruginosa*	6.25.00 ± 0.00	200.00 ± 0.00
	*E. coli*	50.00 ± 0.00	200.00 ± 0.00
	*K. pneumonia*	75.00 ± 0.00	–

Note: Values are expressed as the mean ± SEM (n = 3); Abbreviations: MIC, minimum inhibitory concentration; MBC, minimum bactericidal concentration.

### In vivo antibacterial activities

#### Wound contraction in *S. aureus*-infected mice.

**Fig 1:**
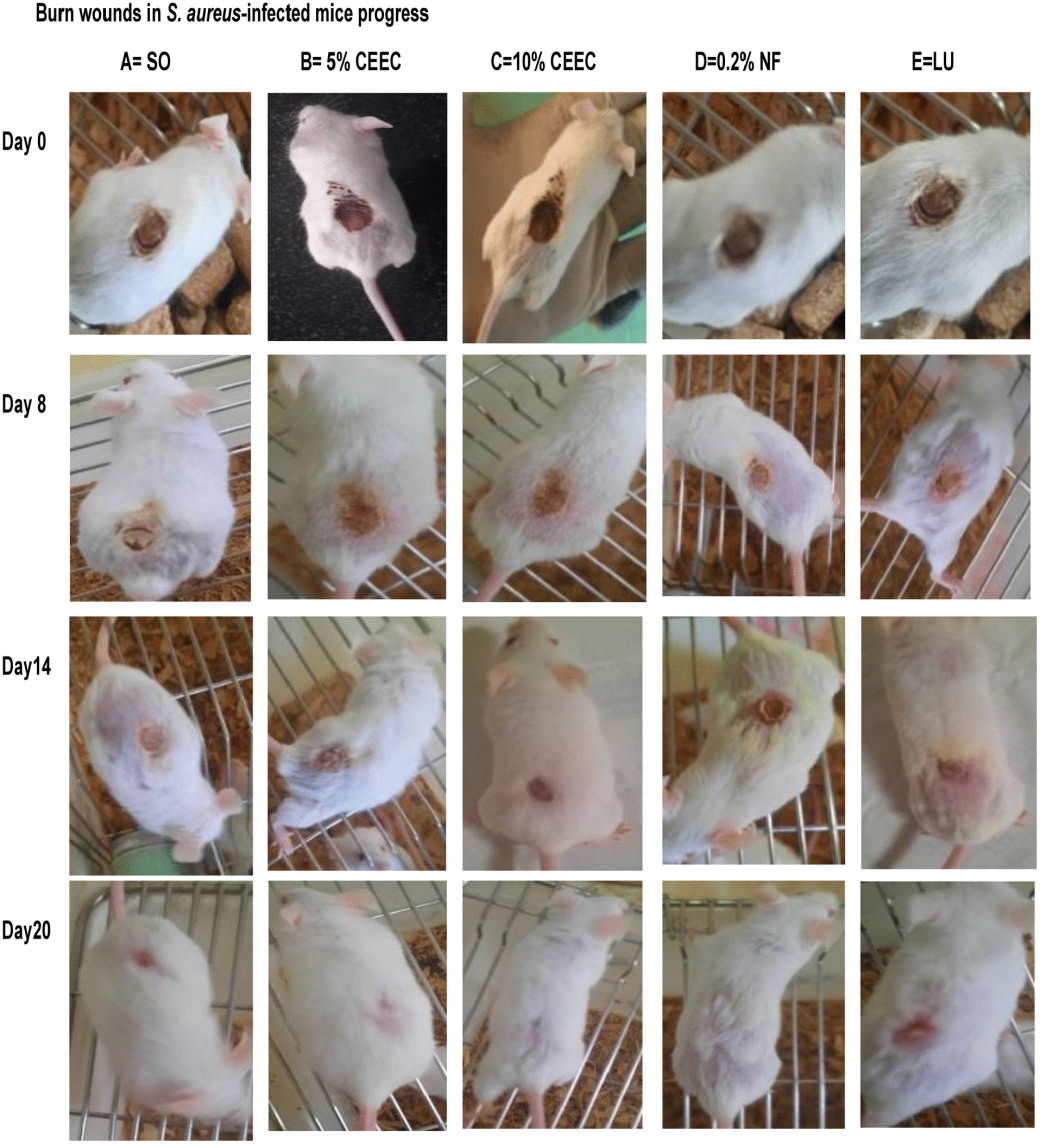
Burn wounds followed by *S. aureus*-infected mice progress: SO-simple ointment, CEEC-Crude Extract *E. cymosa*, NF-nitrofurazone, and LU- left untreated.

**Fig 2:**
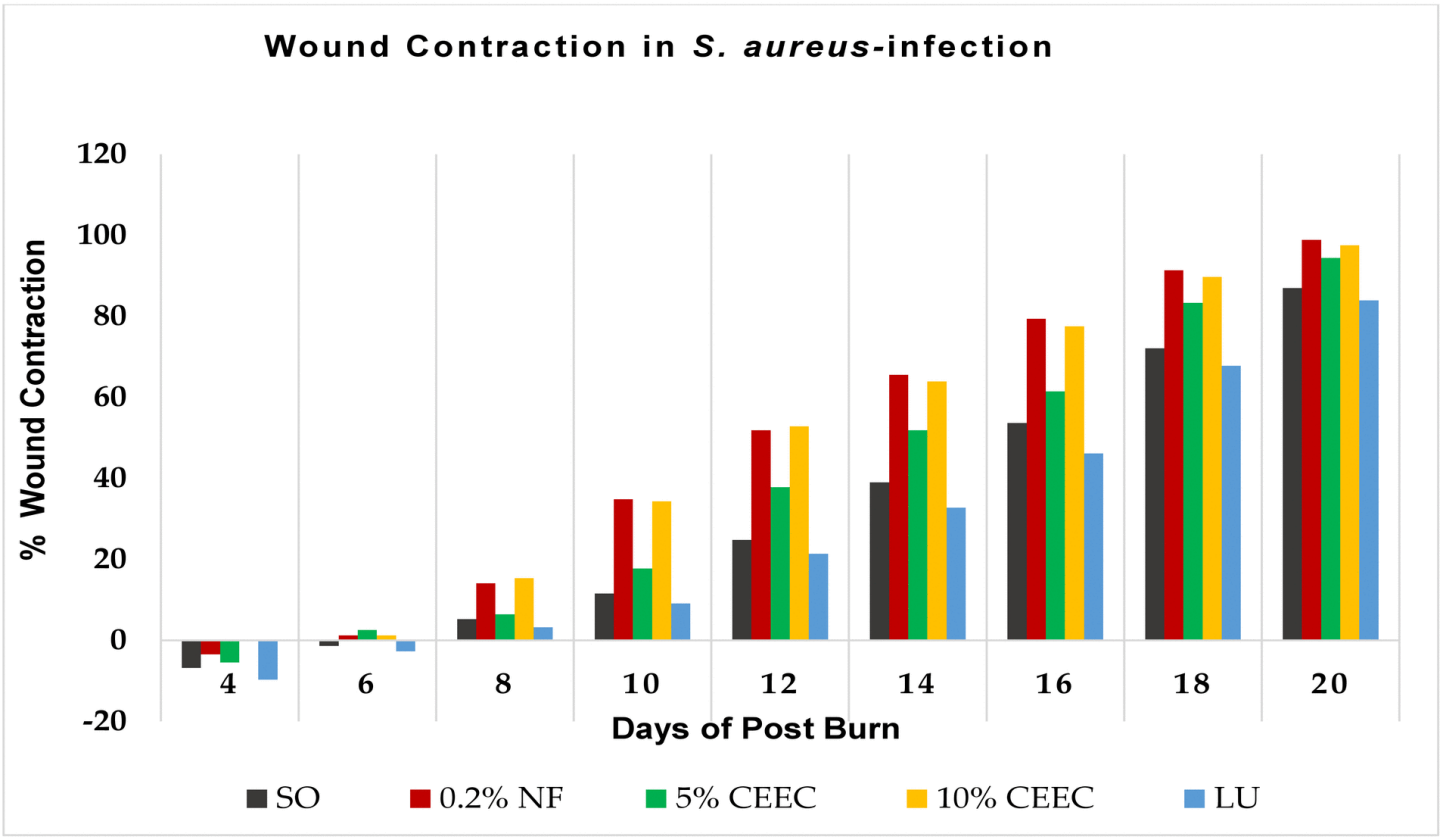
Wound contraction in *S. aureus*-infected mice: SO-simple ointment, NF-nitrofurazone, CEEC-Crude Extract *E. cymosa*, and LU- left untreated.

For the first few days, wound contraction was negative. On the 8th day after the burn, the 10% crude extract ointment (p < 0.01) and the positive control (p < 0.05) showed significant wound contraction compared with the negative control (SO-treated). On the 10th day after the burn, the 5% extract ointment had a substantial effect (p < 0.05). Furthermore, beginning on the 8th day after the burn, the effects of the 10% and 5% crude extracts differed significantly (Table 4). Starting on the 10th day, the 10% (w/w) crude extract ointment of *E. cymosa* leaves and the 0.2% NF ointment showed the highest rate of wound contraction compared with the negative SO-treated control and untreated groups (p < 0.001) (Figs 1 and 2) (S1 Table).

**Table 4:** Effects of 80% methanol extract of the leaves of *E. cymosa* on *S. aureus-infected* wounds in mice.

Days	Wound area (mm^2^) ± SEM (% contraction) *S. aureus-*infection				
	SO ^a^	0.2% NF ^b^	5% CEEC ^c^	10% CEEC ^d^	LU ^e^
**4**	30.19 ± 0.62 (−6.82)	29.24 ± 0.87 (−3.45)	29.81 ± 0.73 (−5.47)	28.27 ± 0.60 (−0.04)	30.99 ± 1.01 (−9.67)
**6**	28.66 ± 0.71 (−1.4)	27.91 ± 0.76 (1.26)	27.53 ± 0.75 (2.58)	27.91 ± 0.76 (1.26)	29.03 ± 0.47 (−2.71)
**8**	26.79 ± 0.62 (5.21)	24.285 ± 0.66 (14.1) ^a*e**^	26.43 ± 0.81 (6.47)	23.93 ± 0.42 (15.33) ^a** c*e**^	27.34 ± 0.58 (3.26)
**10**	24.99 ± 1.48 (11.58)	18.42 ± 0.76 (34.82) ^a***c**e***^	23.25 ± 0.64 (17.73) ^e*^	18.55 ± 0.46 (34.35) ^a***c***e***^	25.69 ± 0.44 (9.09)
**12**	21.24 ± 0.52 (24.84)	13.60 ± 0.49 (51.87) ^a***c*e***^	17.57 ± 1.22 (37.82) ^a*e**^	13.33 ± 0.32 (52.82) ^a***c**e***^	22.23 ± 0.41 (21.36)
**14**	17.24 ± 0.88 (39.00)	9.73 ± 0.27 (65.56) ^a***c***e***^	13.60 ± 0.49 (51.87) ^a**e***^	10.19 ± 0.36 (63.96) ^a***c***e***^	19.01 ± 0.38 (32.73)
**16**	13.09 ± 0.50 (53.69) ^e*^	5.82 ± 0.21 (79.42) ^a***c***e***^	10.88 ± 0.48 (61.49) ^a*e***^	6.35 ± 0.33 (77.53) ^a***c***e***^	15.21 ± 0.44 (46.18)
**18**	7.88 ± 0.57 (72.11)	2.45 ± 0.21 (91.33) ^a***c**e***^	4.72 ± 0.42 (83.31) ^a**e***^	2.90 ± 0.15 (89.73) ^a***c**e***^	9.11 ± 0.57 (67.76)
**20**	3.68 ± 0.26 (86.98) ^e*^	0.31 ± 0.19 (98.89) ^a***c**e***^	1.58 ± 0.22 (94.42) ^a***e***^	0.69 ± 0.31 (97.56) ^a***c*e***^	4.53 ± 0.24 (83.96)

Note: Values are expressed as the mean ± SEM (n = 5); n = 5 mice in each group. a Compared with the negative control (SO), b compared with the positive control (NF), c compared with 5% CEEC, d compared with 10% CEEC, and e compared with the untreated negative control. *P < 0.05; **P < 0.01; ***P < 0.001. Abbreviations: CEEC, crude extract of Ehretia cymosa; SO, simple ointment base (negative control); NF, nitrofurazone; LU, left untreated.

### Wound contraction in *P. aeruginosa*-infected mice

**Fig 3:**
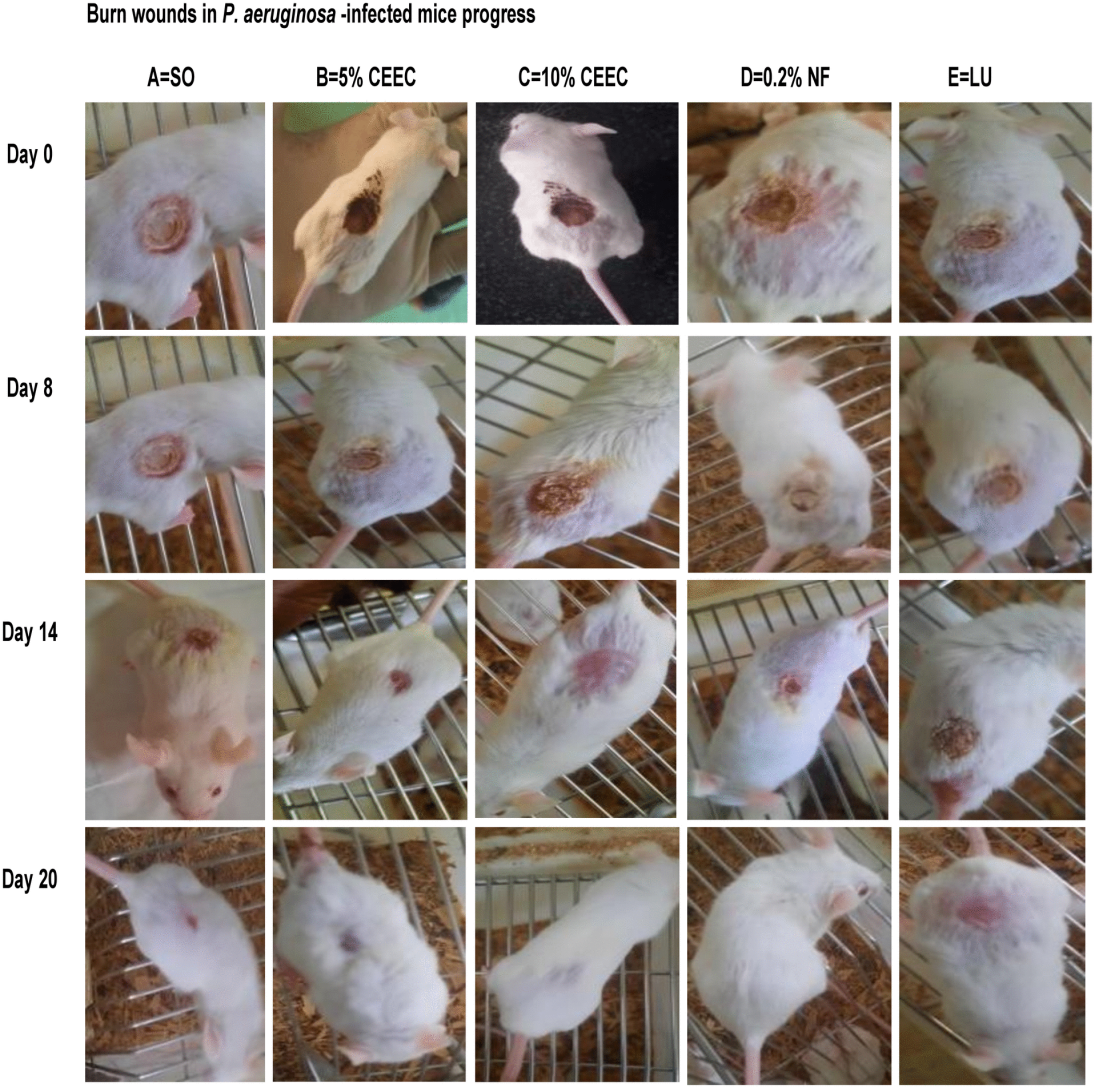
Burn wounds followed by *P. aeruginosa*-infected mice progress: SO-simple ointment, NF-nitrofurazone, CEEC-Crude Extract of *E. cymosa*, and LU- left untreated.

**Fig 4:**
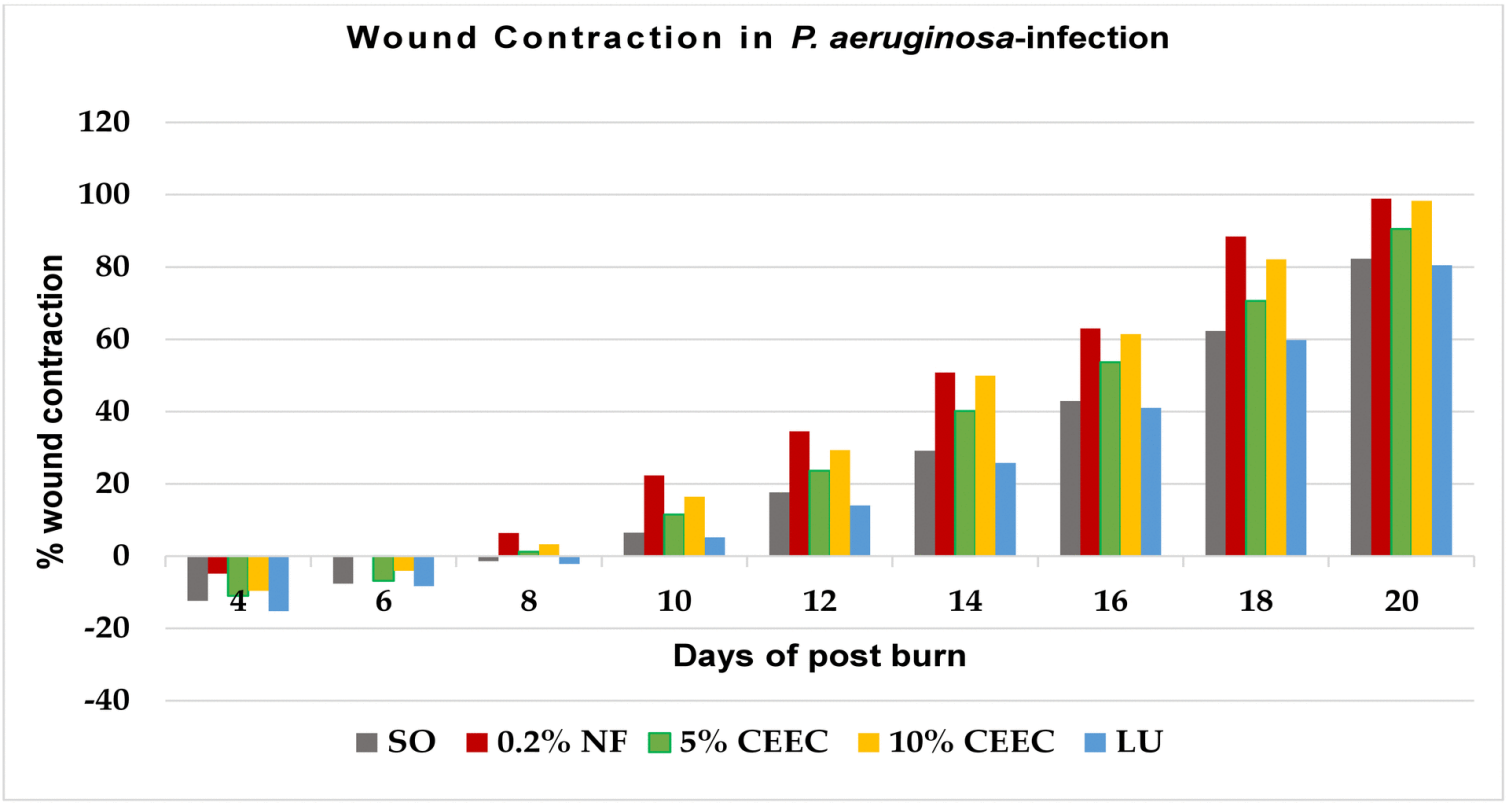
Wound contraction in *P. aeruginosa*-infected mice: SO-simple ointment, NF-nitrofurazone, CEEC-Crude Extract of *E. cymosa*, and LU- left untreated.

On the 10th day post-burn, the 10% extract-treated group showed considerable wound contraction compared to the negative control (SO) (p < 0.05) and untreated groups (p < 0.01). Compared to the 5% (w/w) extract-treated group, the 10% extract-treated group showed considerable wound contraction (p < 0.01) from day 16 onward. The 5% extract-treated group demonstrated considerable wound contraction (p < 0.05) on the 14th day post-burn compared to the negative control and untreated groups. There was no discernible difference between the 10% crude extract ointment formulation and the positive control (0.2% NF). On the 16th day, the 10% ointment and 0.2% NF ointment showed the highest rate of wound contraction (p < 0.001) compared to the untreated groups (Table 5). On the 18th day, it was higher than the SO-treated negative control (Figs 3 and 4) (S2 Table).

**Table 5:** Effects of 80% methanol extract of the leaves of *E. cymosa* on *P. aeruginosa-infected* wounds in mice.

	Wound area (mm^2^) ± SEM (% contraction) *P. aeruginosa-*infection				
Days	SO ^a^	0.2% NF ^b^	5% CEEC ^c^	10% CEEC ^d^	LU ^e^
**4**	31.77 ± 0.75 (−12.42)	29.64 ± 1.23 (−4.89)	31.36 ± 0.48 (−10.98)	30.98 ± 0.78 (−9.62)	32.57 ± 0.75 (−15.27)
**6**	30.42 ± 1.26 (−7.65)	28.29 ± 1.01 (−0.12)	30.20 ± 0.87 (−6.87)	29.42 ± 0.76 (−4.11)	30.61 ± 1.15 (−8.31)
**8**	28.67 ± 0.88 (−1.43)	26.45 ± 1.01 (6.42)	27.93 ± 1.08 (1.18)	27.34 ± 0.58 (3.26)	28.86 ± 1.03 (−2.14)
**10**	26.43 ± 0.81 (6.47)	21.95 ± 1.27 (22.31) ^a*e**^	25.01 ± 1.04 (11.49)	23.61 ± 0.88 (16.47) ^a*e*^	26.79 ± 0.68 (5.20)
**12**	23.27 ± 1.00 (17.64)	18.51 ± 1.50 (34.51) ^a*e*^	21.60 ± 0.97 (23.58)	19.97 ± 0.79 (29.33) ^a*e**^	24.30 ± 0.86 (14.02)
**14**	20.01 ± 1.18 (29.20)	13.90 ± 0.85 (50.82) ^a**e**^	16.92 ± 0.54 (40.13) ^a*e*^	14.14 ± 0.67 (49.96) ^a**e**^	20.97 ± 1.20 (25.80)
**16**	16.12 ± 1.17 (42.96)	10.44 ± 0.67 (63.04) ^a**c**e***^	13.10 ± 0.66 (53.64) ^a*e*^	10.90 ± 0.59 (61.44) ^a**c**e***^	16.66 ± 0.93 (41.04)
**18**	10.66 ± 0.59 (62.27)	3.27 ± 0.13 (88.42) ^a***c***e***^	8.30 ± 0.67 (70.64) ^a*e**^	5.06 ± 0.57 (82.11) ^a***c**e***^	11.36 ± 0.53 (59.80)
**20**	4.99 ± 0.19 (82.33)	0.31 ± 0.19 (98.89) ^a***c**e***^	2.67 ± 0.47 (90.56) ^a*e**^	0.47 ± 0.19 (98.33) ^a***c**e***^	5.51 ± 0.49 (80.49)

Note: Values are expressed as the mean ± SEM (n = 5); n = 5 mice in each group. a compared with the negative control (SO), b compared with the positive control (NF), c compared with 5% CEEC, d compared with 10% CEEC, and e compared with the untreated negative control. *P < 0.05; **P < 0.01; ***P < 0.001. Abbreviations: CEEC, crude extract of Ehretia cymosa; SO, simple ointment base (negative control); NF, nitrofurazone; LU, left untreated.

#### Epithelialization in *S. aureus* and *P. aeruginosa*-infected mice.

The epithelialization period was shorter in the *E. cymosa* ointment preparation- and nitrofurazone-treated groups than in the negative control group. The 10% and 5% extract ointments reduced the epithelialization period (17.4% and 17.6%, respectively). Compared with the negative control, SO (P < 0.05) and the untreated group (P < 0.01) (Table 6), both the 5% and 10% extract ointment groups showed significant differences in epithelialization time.

**Table 6:** Effects of the 80% methanol extract of the leaves of *E. cymosa* on the epithelialization period in the wound-infected model.

Groups	Epithelialization Period (Mean ± SEM), in Days	
	*S. aureus*	*P. aeruginosa*.
SO ^a^	19.20 ± 0.37	19.80 ± 0.20
0.2% NF ^b^	17.20 ± 0.37 ^a**e**^	18.40 ± 0.49 ^a**e***^
5% CEEC ^c^	17.60 ± 0.40 ^a*e**^	19.40 ± 0.40
10% CEEC ^d^	17.40 ± 0.40 ^a*e**^	18.80 ± 0.49 ^e*^
LU ^e^	19.60 ± 0.40	20.00 ± 0.00

Note: Values are expressed as the mean ± SEM (n = 5); n = 5 mice in each group: a compared with the negative control (SO), b compared with the positive control (NF), c compared with 5% CEEC, d compared with 10% CEEC, and e compared with LU. *P < 0.05; **P < 0.01; ***P < 0.001. Abbreviations: CEEC, crude extract of *Ehretia cymosa*; SO, simple ointment base (negative control); NF, nitrofurazone; LU, left untreated.

The 10% extract ointment (18.80%) and the 5% extract ointment (19.40%) reduced the epithelialization period. The standard treatment (positive control) resulted in a faster rate of epithelialization than the negative control (SO) (P < 0.01) and left untreated (LU) (P < 0.001). Moreover, the 10% extract ointment had a significant effect on the epithelialization period compared with the negative LU group (P < 0.05) (Table 6).

### Phytochemical screening

Phytochemical screening of the 80% methanol extract of *E. cymosa* leaves revealed the presence of secondary metabolites, such as alkaloids, saponins, flavonoids, terpenoids, phenols, glycosides, and tannins (Table 7).

**Table 7:** Results of the phytochemical screening of 80% methanol extracts of *E. cymosa* leaves.

Secondary Metabolites	Test type	Controls color	Test Results
Alkaloids	Wagner’s test	Redish-brown	+
Tannins	Ferric chloride test	Blue‒black	+
Saponins	Froth/foam test	Persistent foam	+
Flavonoids	Shinoda test	Intense yellow	+
Terpenoids	Salkowski test	Reddish brown at the interface	+
Phenols	Ferric chloride test	Deep blue	+
Steroids	Salkowski test	Blue‒green	+
Glycosides	Kellar-Killiani test	Brown ring at the interface	+

Prescence (+) or absence (-).

## Discussion

The in vitro antibacterial activity test of the 80% methanol extract of *E. cymosa* leaves revealed zones of inhibition greater than 7 mm in diameter at all the tested concentrations (100, 200, and 300 mg/mL), indicating measurable antibacterial activity. The extract demonstrated notable effectiveness against *S. aureus, P. aeruginosa*, and *E. coli.* Compared with the negative controls, the in vivo model demonstrated better effectiveness against *S. aureus* than against *P. aeruginosa*, as evidenced by faster wound contraction and a shorter epithelialization time. These findings suggest that this plant is a promising candidate for further purification and investigation of its bioactive compounds in the treatment of infectious diseases.

The results of the present study revealed that *S. aureus* was more susceptible than the other tested strains, with a 17 mm zone of inhibition at 100 mg/mL, followed by *P. aeruginosa* at 11 mm. Previous studies classified antibacterial activity based on the zone of inhibition (ZOI) as strong activity: ZOI ≥ 15 mm or 20 mm; moderate activity: ZOI 10–14 mm; and weak activity: ZOI < 10 mm or 2–6 mm [69–71]. Hence, the plant extract has strong activity against *S. aureus,* moderate activity against *P. aeruginosa* and *E. coli,* and weak activity against *S. pyogenes and K. pneumoniae* at 100 mg/mL (Table 2). The effect also increased significantly in a dose-dependent manner.

*S. aureus* generally appears to be more sensitive to some antibiotics than *P. aeruginosa* is because *P. aeruginosa* has a tough outer membrane (gram-negative structure) that blocks entry and uses efflux pumps to expel drugs, whereas *S. aureus* (gram-positive) lacks this outer shield, making it inherently more accessible to antibiotics [72–74]. Apparently, in mixed infections, *S. aureus* often seems more susceptible to antibiotics than *P. aeruginosa;* however, *P. aeruginosa* produces compounds that inhibit *S. aureus* metabolism, help *S. aureus* form protective biofilms, and can trigger *S. aureus* to become dormant (small colony variants), increasing the difficulty of killing with many drugs [74–80].

In a previous study conducted against four microbial isolates (*S. aureus, P. aeruginosa, E. coli,* and *B. subtilis*), the results indicated that the 70% ethanol extract of the whole plant material *E. cymosa* exhibited inhibitory activity and that *E. coli* was the most susceptible, with an MIC of 0.0108 μg/mL, whereas *P. aeruginosa* was the least susceptible, with an MIC of 0.1744 μg/mL [17]. In another study, the antibacterial activity of *E. cymosa* was investigated against *S. aureus and P. aeruginosa* at a concentration of 25 mg/mL; the zones of inhibition observed by the ethyl acetate extract against *P. aeruginosa* and *S. aureus* were found to be 16 mm and 13 mm at 25 mg/mL, respectively [81]. In the present study, larger doses of the crude extract were used to observe a dose-dependent effect. In addition, crude extracts contain complex combinations that can have both antagonistic (decreased) and synergistic (increased) effects, whereas pure drugs provide predictable, unique results [82]. Owing to differences in chemical complexity, mechanisms of action, and standardization, it is challenging to compare the effects of a pure standard drug with those of a crude plant extract [83,84]. However, these findings might support some of the uses reported by traditional healers for infection treatment. The antibacterial effect is important in treating bacterial infections in humans.

In this study, the MIC of *E. cymosa* ranged from 6.25 to 75 mg/mL against standard bacterial strains; however, the MBC was 200 mg/mL against *E. coli* and *P. aeruginosa,* and no bactericidal activity was observed against *S. aureus, S. pyogenes, or K. pneumoniae* at the tested doses (Table 3). Antibacterial activity can be classified based on MIC: highly potent (<0.5 mg/mL/μg/mL), moderately potent (0.5 to 1.5 mg/mL), and weak/low (>1.5 mg/mL, often up to 10 mg/mL or higher) [85,86]. Hence, the *E. cymosa* extract showed weak antibacterial activity, with relatively potent activity against *P. aeruginosa* (6.25 mg/mL) compared with other strains.

Knowledge of the MIC and MBC allows the most appropriate antimicrobial therapy to be developed, thereby decreasing the use of broad-spectrum antibiotics and the development of resistance [86,87]. The MBC/MIC ratio indicates whether an antibiotic is primarily bactericidal or bacteriostatic [87]. A low ratio (typically ≤4) suggests a bactericidal effect, which is important for severe infections needing rapid bacterial elimination [88]. A high ratio (> [4]) indicates a primarily bacteriostatic effect, where the antibiotic inhibits growth and relies on the host’s immune system to clear the infection [89]. In line with these findings, *E. cymosa* leaf extract might be bactericidal for *E. coli* and bacteriostatic for the other tested strains. In addition, it appears to have an MIC = 6.25 mg/mL against *P. aeruginosa* but has a high MBC = 200 mg/mL (Table 3), which can indeed suggest that higher concentrations of the drug are needed to kill the bacteria, which may indicate that the extract is bacteriostatic rather than bactericidal, or that it has the potential for increased toxicity [90,91].

The absence of MBC alongside a strong zone of inhibition in an agar diffusion assay typically indicates that the tested agent is bacteriostatic rather than bactericidal. The agar diffusion method primarily measures the susceptibility and growth-inhibiting power of an antimicrobial agent as it diffuses through the agar, but it does not differentiate between inhibiting bacterial growth and killing the bacteria [92]. Here are the specific scientific reasons researchers can use to explain this discrepancy: The zone of inhibition confirms that the agent successfully halts the multiplication of *S. aureus*. However, to determine an MBC, the agent must kill 99.9% of the initial bacterial inoculum. If the antibiotic merely briefly inhibits growth, the bacteria will return and regenerate when subcultured into fresh broth or onto agar without the antimicrobial agent, implying that no MBC can be formed [93]. Besides, broth dilution assays may exhibit a high tolerance or enter a dormant “persister/tolerance” state. The agent may inhibit bacteria at the MIC without completely eradicating them, requiring extremely high concentrations to achieve a bactericidal effect [94]. It might also be attributed to diffusion rate or solubility: Agar diffusion effectiveness depends heavily on the physicochemical properties of the antimicrobial agent. Hydrophilic or low-molecular-weight compounds diffuse very efficiently through the agar gel, forming large zones of inhibition. However, these same compounds might lack the high chemical potency or concentration required to irreversibly kill the bacteria once they are suspended in liquid broth (where the MIC is evaluated) [41]. As a result, alternate assays to confirm whether the drug is largely static or cidal, as well as ways to overcome solubility or agar-binding constraints in their MBC assays, must be carried out.

**Fig 5:**
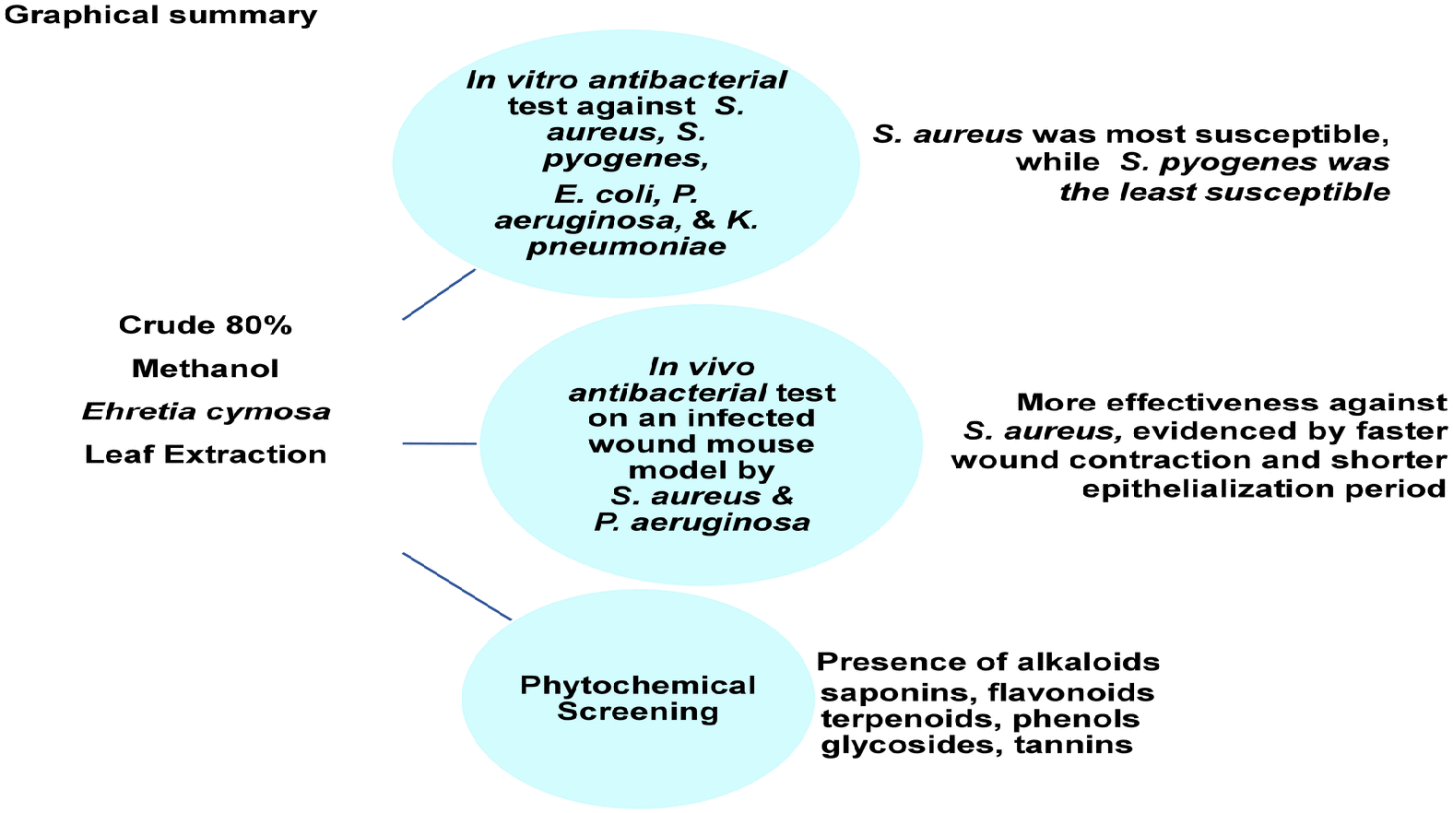
Summary of antibacterial activities and phytochemical screening of 80% methanol crude *E. cymosa* leaf extract.

To further investigate the antibacterial activity of this strain and its traditional claims, an in vivo model—burn followed by infection—was employed. An ointment containing a crude extract of *E. cymosa* leaves had a statistically significant effect on wounds infected with *S. aureus* and *P. aeruginosa* in mice. Wound infiltration, blister formation, and edema cleared up faster in the extract-treated groups than in the negative controls (Figs 1 and 3). The extract ointment resulted in a faster wound contraction rate in the groups treated with 10% extract ointment than in those treated with 5% extract ointment and the negative control groups (Tables 4 and 5), as well as a shorter duration of epithelialization than the negative controls did (Table 6). The plant also showed improved activity against *S. aureus* than against *P. aeruginosa,* as shown by the in vitro susceptibility test results (Fig 5).

According to research, removing invading organisms from infected wounds promotes healing [95]. In this study, because the groups treated with simple ointment and the untreated group showed a slow rate of wound area contraction and did not re-epithelialize during the follow-up period, the extract’s antibacterial effect against common wound-infecting microorganisms may support faster wound healing in this trial. Infection is a substantial barrier to wound healing since it can disrupt the healing process and, in severe cases, lead to death [95–98].

Phytochemical screening, which is a rapid, cost-effective process that provides scientific validation for traditional remedies and aids in the development of potent treatments against diseases and microbes, is crucial for identifying bioactive compounds in plants [84,99,100]. Accordingly, in the present study, phytochemical screening of the *E. cymosa* leaf extract revealed the presence of terpenoids, flavonoids, alkaloids, saponins, tannins, phenols, steroids, and glycosides (Table 7). Studies have indicated that flavonoids, phenols, terpenoids, and tannins are secondary metabolites with high antibacterial potential [16,17,81,101,102]. Flavonoids possessing ortho-hydroxyl groups on the B-ring interact with the peptidoglycan of Gram-positive bacteria through hydrogen bonding, leading to cell wall instability and, ultimately, lysis [103,104]. It can also depolarize the cell membrane, dissipate the proton motive force (PMF), and disrupt ATP synthesis, which halts bacterial growth [105]. In addition, flavonoids such as rutin can interfere with quorum sensing, reducing the expression of virulence factors and biofilm formation by pathogens [102]. Phenolic compounds also attack bacterial phospholipid membranes, increasing permeability and causing leakage of the cytoplasm and ions [106,107].

### Strengths and limitations of the study

This study employed both in vitro and in vivo models to explore the antibacterial activity of *E. cymosa*. However, the use of a crude extract limited the results, and the in vivo study lacked a quantitative assessment of the bacterial load (colony-forming units) before and after treatment, as well as microbiological confirmation that the extract-treated wounds had a reduced bacterial burden. This gap limits the ability to determine whether the observed effects are truly antibacterial or wound-healing.

## Conclusions

The present study demonstrated that the 80% methanol extract of *E. cymosa* leaves has antibacterial activity in both in vitro and in vivo. The effect was concentration-dependent against the tested pathogens. The activity against *P. aeruginosa* started at the lowest concentration; however, better dose-dependent growth inhibition was observed in the in vitro test against *S. aureus*. In the in vivo model, faster wound contraction and a shorter epithelialization period were observed for *S. aureus* than for *P. aeruginosa*. These findings support its potential as a source of natural antimicrobial agents. Further isolation and characterization of active compounds, as well as wound-healing mechanisms, are recommended.

## Supporting information

S1 Table Wound contraction in *S. aureus* infection. SO-simple ointment, NF-nitrofurazone, CEEC-Crude Extract E. cymosa, and LU- left untreated.
(DOCX)

S2 Table Wound contraction in *P. aeruginosa* infection. SO-simple ointment, NF-nitrofurazone, CEEC-Crude Extract E. cymosa, and LU- left untreated.
(DOCX)

## References

[1] Cook MA, Wright GD. The past, present, and future of antibiotics. Sci Transl Med. 2022;14(657):eabo7793. doi: 10.1126/scitranslmed.abo7793 35947678

[2] Mohr KI. History of antibiotics research. 2016. 10.1007/82_2016_499 27738915

[3] Roose-Amsaleg C, Laverman AM. Do antibiotics have environmental side-effects? Impact of synthetic antibiotics on biogeochemical processes. Environ Sci Pollut Res Int. 2016;23(5):4000–12. doi: 10.1007/s11356-015-4943-3 26150293

[4] Singh R, Sripada L, Singh R. Side effects of antibiotics during bacterial infection: mitochondria, the main target in host cell. Mitochondrion. 2014;16:50–4. doi: 10.1016/j.mito.2013.10.005 24246912

[5] AlSheikh HMA, Sultan I, Kumar V, Rather IA, Al-Sheikh H, Tasleem Jan A, et al. Plant-based phytochemicals as possible alternative to antibiotics in combating bacterial drug resistance. Antibiotics (Basel). 2020;9(8):480. doi: 10.3390/antibiotics9080480 32759771 PMC7460449

[6] Dutt B, Kumar S, Garg KC. Mapping of plant-based medicine research in China and India. Res Eval. 2009;18(1):51–9. doi: 10.3152/095820209x393154

[7] van Wyk AS, Prinsloo G. Health, safety and quality concerns of plant-based traditional medicines and herbal remedies. South African J Botany. 2020;133:54–62. doi: 10.1016/j.sajb.2020.06.031

[8] Majeed M. Evidence-based medicinal plant products for the health care of world population. Ann Phytomed. 2017;VI(I):1–4. doi: 10.21276/ap.2017.6.1.1

[9] Wijesekera ROB. Plant-derived medicines and their role in global health. The medicinal plant industry. Routledge; 2017. 1–18. doi: 10.1201/9780203736395-1

[10] Wanga L, Nyamboki DK. Medicinal plants used in the management of skin disorders in kenya: a review. Pharmacogn Rev. 2023;69–103. doi: 10.5530/097627870276

[11] Christensen SB. Drugs that changed society: history and current status of the early antibiotics: salvarsan, sulfonamides, and β-lactams. Molecules. 2021;26(19):6057. doi: 10.3390/molecules26196057 34641601 PMC8512414

[12] Albahri G, Badran A, Hijazi A, Daou A, Baydoun E, Nasser M, et al. The therapeutic wound healing bioactivities of various medicinal plants. Life (Basel). 2023;13(2):317. doi: 10.3390/life13020317 36836674 PMC9960863

[13] Shedoeva A, Leavesley D, Upton Z, Fan C. Wound healing and the use of medicinal plants. Evid Based Complement Alternat Med. 2019;2019:2684108. doi: 10.1155/2019/2684108 31662773 PMC6778887

[14] Comino-Sanz IM, López-Franco MD, Castro B, Pancorbo-Hidalgo PL. The role of antioxidants on wound healing: a review of the current evidence. J Clin Med. 2021;10(16):3558. doi: 10.3390/jcm10163558 34441854 PMC8397081

[15] Jiang S, Wang M, Kaur A, Jiang L, Cai Y, Luo J, et al. Ehretia genus: a comprehensive review of its botany, ethnomedicinal values, phytochemistry, pharmacology, toxicology and clinical studies. Frontiers in Pharmacology. 2025;16. doi: 10.3389/fphar.2025.1526359 PMC11885236 40061956

[16] Ashagrie G, Abebe A, Umer S. Analgesic and anti-inflammatory activities of 80% methanol extract and solvent fractions of ehretia cymosa thonn (Boraginaceae) leaves in rodents. J Exp Pharmacol. 2023;15:63–79. doi: 10.2147/JEP.S396769 36864852 PMC9970881

[17] Sarkodie J, Squire S, Kretchy I, Domozoro C, Ahiagbe K, Twumasi M. The antihyperglycemic, antioxidant and antimicrobial activities of Ehretia cymosa. J Pharm Phytochem. 2015;4(3):105–11.

[18] Ogundajo A, Ashafa AT. Phytochemical compositions and in vitro assessments of antioxidant and antidiabetic potentials of fractions from ehretia cymosa thonn. Pharmacogn Mag. 2017;13(Suppl 3):S470–80. doi: 10.4103/pm.pm_118_17 29142401 PMC5669084

[19] Council NR, Research IfLA. Guidance for the description of animal research in scientific publications. National Academies Press; 2011. 22379656

[20] Woodward R. The organisation for economic co-operation and development (OECD). Routledge; 2009.

[21] Council NR, Earth D o, Studies L, Research IfLA. Guidance for the description of animal research in scientific publications. 2011. 22379656

[22] Chen J, Hao D, Mei K, Li X, Li T, Ma C, et al. Microbiology spectrum. 2021;9(3):e01318-21. doi: 10.1128/spectrum.01318-21 PMC8672897 34908502

[23] Levison ME, Levison JH. Pharmacokinetics and pharmacodynamics of antibacterial agents. Infect Dis Clin North Am. 2009;23(4):791–815, vii. doi: 10.1016/j.idc.2009.06.008 19909885 PMC3675903

[24] Pharmacopoeia B, Department of health and social security Scottish home and health department, Office of the British Pharmacopoeia Commission. UK. 1988.

[25] Worku S, Abebe T, Alemu A, Seyoum B, Swedberg G, Abdissa A, et al. Bacterial profile of surgical site infection and antimicrobial resistance patterns in Ethiopia: a multicentre prospective cross-sectional study. Ann Clin Microbiol Antimicrob. 2023;22(1):96. doi: 10.1186/s12941-023-00643-6 37936207 PMC10631106

[26] Kulayta K, Zerdo Z, Seid M, Dubale A, Manilal A, Kebede T, et al. Biofilm formation and antibiogram profile of bacteria from infected wounds in a general hospital in southern Ethiopia. Sci Rep. 2024;14(1):26359. doi: 10.1038/s41598-024-78283-9 39487302 PMC11530625

[27] Sisay M, Worku T, Edessa D. Microbial epidemiology and antimicrobial resistance patterns of wound infection in Ethiopia: a meta-analysis of laboratory-based cross-sectional studies. BMC Pharmacol Toxicol. 2019;20(1):35. doi: 10.1186/s40360-019-0315-9 31146791 PMC6543595

[28] Shimekaw M, Tigabu A, Tessema B. Bacterial profile, antimicrobial susceptibility pattern, and associated risk factors among patients with wound infections at debre markos referral Hospital, Northwest, Ethiopia. Int J Low Extrem Wounds. 2022;21(2):182–92. doi: 10.1177/1534734620933731 32594808

[29] Bandy A, Wani FA, Mohammed AH, Dar UF, Mallick A, Dar MR, et al. Bacteriological profile of wound infections and antimicrobial resistance in selected gram-negative bacteria. Afr Health Sci. 2022;22(4):576–86. doi: 10.4314/ahs.v22i4.63 37092080 PMC10117509

[30] Puca V, Marulli RZ, Grande R, Vitale I, Niro A, Molinaro G, et al. Microbial species isolated from infected wounds and antimicrobial resistance analysis: data emerging from a three-years retrospective study. Antibiotics (Basel). 2021;10(10):1162. doi: 10.3390/antibiotics10101162 34680743 PMC8532735

[31] Geremew T, Yalemtsehay M, Eyasu M, Runner RTM, Gomotsang B-M, Samuel OY. Antibacterial activity of crude extracts and pure compounds isolated from Vernonia galamensis leaves. Afr J Pharm Pharmacol. 2018;12(11):136–41. doi: 10.5897/ajpp2018.4888

[32] Ismael HM. In vitro evaluation of pomegranate (Punica granatum) peel extract against candida krusei as an alternative agent to antifungal medicines. Zanco Journal of Pure and Applied Sciences. 2022;34(1):69–79.

[33] Bubonja-Šonje M, Knežević S, Abram M. Challenges to antimicrobial susceptibility testing of plant-derived polyphenolic compounds. Arh Hig Rada Toksikol. 2020;71(4):300–11. doi: 10.2478/aiht-2020-71-3396 33410777 PMC7968511

[34] Balouiri M, Sadiki M, Ibnsouda SK. Methods for in vitro evaluating antimicrobial activity: a review. J Pharm Anal. 2016;6(2):71–9. doi: 10.1016/j.jpha.2015.11.005 29403965 PMC5762448

[35] Mostafa AA, Al-Askar AA, Almaary KS, Dawoud TM, Sholkamy EN, Bakri MM. Antimicrobial activity of some plant extracts against bacterial strains causing food poisoning diseases. Saudi J Biol Sci. 2018;25(2):361–6. doi: 10.1016/j.sjbs.2017.02.004 29472791 PMC5815983

[36] Yemata G, Desta B, Fetene M. In vitro antibacterial activity of traditionally used medicinal plants against Xanthomonas campestris pv. musacearum in Ethiopia. Biodiversitas. 2019;20(2):555–61. doi: 10.13057/biodiv/d200235

[37] Hudzicki J, Jones J. Teaching clinical chemistry on a shoestring budget. Clinical Laboratory Science. 2016;29(2).

[38] Cazedey ECL, Salgado HRN. A novel and rapid microbiological assay for ciprofloxacin hydrochloride. J Pharm Anal. 2013;3(5):382–6. doi: 10.1016/j.jpha.2013.03.007 29403843 PMC5761008

[39] Khwaza V, Mlala S, Aderibigbe BA. Advancements in synthetic strategies and biological effects of ciprofloxacin derivatives: a review. Int J Mol Sci. 2024;25(9):4919. doi: 10.3390/ijms25094919 38732134 PMC11084713

[40] Alrifai AAA. Comparative study of the antibacterial activity of selected ciprofloxacin tablet brands available in Iraq. Toxicol Inter. 2025;32(3):635–42.

[41] Hulankova R. Methods for determination of antimicrobial activity of essential oils in vitro-a review. Plants (Basel). 2024;13(19):2784. doi: 10.3390/plants13192784 39409654 PMC11478843

[42] Eloff JN. Avoiding pitfalls in determining antimicrobial activity of plant extracts and publishing the results. BMC Complement Altern Med. 2019;19(1):106. doi: 10.1186/s12906-019-2519-3 31113428 PMC6530048

[43] Mustapha A, Ndahi N, Paul B, Fugu M. Synthesis, characterization and antimicrobial studies of metal (II) complexes of ciprofloxacin. J Chem Pharm Res. 2014;6(4):588–93.

[44] Zhang J, Qu G, Ma J, Wu G, Lu X, Tan Y. Raman spectroscopic and theoretical investigation of polarity regulation and hydrogen bonding in DMSO binary systems. Spectrochim Acta A Mol Biomol Spectrosc. 2026;355:127667. doi: 10.1016/j.saa.2026.127667 41806767

[45] Andrews JM. Determination of minimum inhibitory concentrations. J Antimicrob Chemother. 2001;48 Suppl 1:5–16. doi: 10.1093/jac/48.suppl_1.5 11420333

[46] Clinical ILS. Performance standards for antimicrobial susceptibility testing. Wayne, PA: Clinical and Laboratory Standards Institute; 2020.

[47] Govekar A, Sonal SG. Determination of minimum inhibitory concentration by broth dilution method-a review. BTRA Scan. 2022;51(2):1–6.

[48] Rodríguez-Melcón C, Alonso-Calleja C, García-Fernández C, Carballo J, Capita R. Minimum inhibitory concentration (MIC) and minimum bactericidal concentration (MBC) for twelve antimicrobials (biocides and antibiotics) in eight strains of listeria monocytogenes. Biology (Basel). 2021;11(1):46. doi: 10.3390/biology11010046 35053044 PMC8773323

[49] Barnes V L, Heithoff DM, Mahan SP, House JK, Mahan MJ. Antimicrobial susceptibility testing to evaluate minimum inhibitory concentration values of clinically relevant antibiotics. STAR Protoc. 2023;4(3):102512. doi: 10.1016/j.xpro.2023.102512 37566547 PMC10448204

[50] Kadeřábková N, Mahmood AJS, Mavridou DAI. Antibiotic susceptibility testing using minimum inhibitory concentration (MIC) assays. NPJ Antimicrob Resist. 2024;2(1):37. doi: 10.1038/s44259-024-00051-6 39843555 PMC11721449

[51] Rowan MP, Cancio LC, Elster EA, Burmeister DM, Rose LF, Natesan S, et al. Burn wound healing and treatment: review and advancements. Crit Care. 2015;19:243. doi: 10.1186/s13054-015-0961-2 26067660 PMC4464872

[52] Charan J, Kantharia ND. How to calculate sample size in animal studies?. J Pharmacol Pharmacother. 2013;4(4):303–6. doi: 10.4103/0976-500X.119726 24250214 PMC3826013

[53] Hong Y, Lin X, Cui X, Zhou L, Al-Rasheid KAS, Li J. Comparative evaluation of genotoxicity induced by nitrofurazone in two ciliated protozoa by detecting DNA strand breaks and DNA–protein crosslinks. Ecological Indicators. 2015;54:153–60. doi: 10.1016/j.ecolind.2015.02.030

[54] Menegat TA, Oliveira AF de, Majewski MGC, Blanes L, Juliano Y, Novo NF, et al. Experimental models of scald burns. A scope review. Acta Cir Bras. 2019;34(10):e201901007. doi: 10.1590/s0102-865020190100000007 31826150 PMC6907881

[55] Wiggins-Dohlvik K, Tharakan B. A rat burn injury model for studying changes in microvascular permeability. Methods Mol Biol. 2018;1717:93–100. doi: 10.1007/978-1-4939-7526-6_8 29468586

[56] Abdullahi A, Amini-Nik S, Jeschke MG. Animal models in burn research. Cell Mol Life Sci. 2014;71(17):3241–55. doi: 10.1007/s00018-014-1612-5 24714880 PMC4134422

[57] Hao D, Nourbakhsh M. Recent advances in experimental burn models. Biology (Basel). 2021;10(6):526. doi: 10.3390/biology10060526 34204763 PMC8231482

[58] Chang S-J, Sartika D, Fan G-Y, Cherng J-H, Wang Y-W. Animal models of burn wound management. Animal Models in Medicine and Biology. IntechOpen; 2020. doi: 10.5772/intechopen.89188

[59] Shukla SK, Sharma AK, Shaw P, Kalonia A, Yashavarddhan MH, Singh S. Creation of rapid and reproducible burn in animal model with a newly developed burn device. Burns. 2020;46(5):1142–9. doi: 10.1016/j.burns.2019.12.005 32169381

[60] Gouma E, Simos Y, Verginadis I, Lykoudis E, Evangelou A, Karkabounas S. A simple procedure for estimation of total body surface area and determination of a new value of Meeh’s constant in rats. Lab Anim. 2012;46(1):40–5. doi: 10.1258/la.2011.011021 22008848

[61] Du Sert NP, Hurst V, Ahluwalia A, Alam S, Avey MT, Baker M. The ARRIVE guidelines 2.0: updated guidelines for reporting animal research. 2020. 10.1371/journal.pbio.3000410 PMC7360023 32663219

[62] Karp NA, Pearl EJ, Stringer EJ, Barkus C, Ulrichsen JC, Percie du Sert N. A qualitative study of the barriers to using blinding in in vivo experiments and suggestions for improvement. PLoS Biol. 2022;20(11):e3001873. doi: 10.1371/journal.pbio.3001873 36395326 PMC9714947

[63] Dilnesa A, Mekonon A, Abebe A. Phytochemical screening and antioxidant activity investigations on the crude extracts of Brucea antidysenterica leaves. Int J Res Dev. 2016;1:131–44.

[64] Shaikh JR, Patil M. Qualitative tests for preliminary phytochemical screening: an overview. Int J Chem Stud. 2020;8(2):603–8. doi: 10.22271/chemi.2020.v8.i2i.8834

[65] Palanisamy P, Basalingappa KM. Phytochemical analysis and antioxidant properties of leaf extracts of Carica papaya. Phytochem Anal. 2020;13(11):58–62.

[66] Khalid S, Shahzad A, Basharat N, Abubakar M, Anwar P. Phytochemical screening and analysis of selected medicinal plants in Gujrat. J Phytochem Biochem. 2018;2(1):1–3.

[67] Tessema Z, Makonnen E, Debella A, Molla Y. Evaluation of in vivo wound healing and anti-inflammatory activity of 80% methanolic extract of the leaves of B. antidysentrica JF Mill (Simaroubaceae) in mice. Asian J Complement Altern Med. 2019;7(1):1–8.

[68] Care I o LARC, Animals UoL. Guide for the care and use of laboratory animals. US Department of Health and Human Services, Public Health Service; 1986.

[69] Fankam AG. Chapter four - screening methods for antibacterial agents from plant source. In: Kuete V, editor. Advances in botanical research. 106: Academic Press; 2023. 61–79.

[70] Rodloff A, Bauer T, Ewig S, Kujath P, Müller E. Susceptible, intermediate, and resistant - the intensity of antibiotic action. Dtsch Arztebl Int. 2008;105(39):657–62. doi: 10.3238/arztebl.2008.0657 19626213 PMC2701059

[71] Yemata G, Yihune E, Kebede Y. Study on antibacterial activities of croton macrostachyus and pycnostachys abyssinica leaf extracts against some human pathogens. ScientificWorldJournal. 2025;2025:9481587. doi: 10.1155/tswj/9481587 39845692 PMC11753855

[72] Ali S, Assafi M. Prevalence and antibiogram of Pseudomonas aeruginosa and Staphylococcus aureus clinical isolates from burns and wounds in Duhok City, Iraq. J Infect Dev Ctries. 2024;18(1):82–92. doi: 10.3855/jidc.18193 38377094

[73] Breijyeh Z, Jubeh B, Karaman R. Resistance of gram-negative bacteria to current antibacterial agents and approaches to resolve it. Molecules. 2020;25(6):Epub 2020-03-16. doi: 10.3390/molecules25061340 32187986 PMC7144564

[74] Piatek M, O’Beirne C, Beato Z, Tacke M, Kavanagh K. Pseudomonas aeruginosa and Staphylococcus aureus display differential proteomic responses to the silver(i) compound, SBC3. Antibiotics (Basel). 2023;12(2):348. doi: 10.3390/antibiotics12020348 36830259 PMC9952281

[75] Radlinski L, Rowe SE, Kartchner LB, Maile R, Cairns BA, Vitko NP, et al. Pseudomonas aeruginosa exoproducts determine antibiotic efficacy against Staphylococcus aureus. PLoS Biol. 2017;15(11):e2003981. doi: 10.1371/journal.pbio.2003981 29176757 PMC5720819

[76] Biswas L, Götz F. Molecular mechanisms of Staphylococcus and Pseudomonas interactions in cystic fibrosis. Front Cell Infect Microbiol. 2022;11:824042. doi: 10.3389/fcimb.2021.824042 35071057 PMC8770549

[77] DeLeon S, Clinton A, Fowler H, Everett J, Horswill AR, Rumbaugh KP. Synergistic interactions of Pseudomonas aeruginosa and Staphylococcus aureus in an in vitro wound model. Infect Immun. 2014;82(11):4718–28. doi: 10.1128/IAI.02198-14 25156721 PMC4249327

[78] Bernardy EE, Raghuram V, Goldberg JB. Staphylococcus aureus and Pseudomonas aeruginosa isolates from the same cystic fibrosis respiratory sample coexist in coculture. Microbiol Spectr. 2022;10(4):e0097622. doi: 10.1128/spectrum.00976-22 35867391 PMC9431432

[79] Scaffo J, Lima RD, Dobrotka C, Ribeiro TAN, Pereira RFA, Sachs D. In vitro analysis of interactions between Staphylococcus aureus and Pseudomonas aeruginosa during biofilm formation. Antibiotics. 2025;14(5):504. doi: 10.3390/antibiotics14050504 40426570 PMC12108489

[80] Camus L, Briaud P, Vandenesch F, Doléans-Jordheim A, Moreau K. Mixed populations and co-infection: Pseudomonas aeruginosa and Staphylococcus aureus. Advances in Experimental Medicine and Biology. 2022. 397–424. doi: 10.1007/978-3-031-08491-1_15 36258081

[81] Yadessa M, Hailemichael T, Aman D, Teshome A. Antibacterial triterpenoid from the leaves extract of Ehretia cymosa. Ethiopian J Sci Sustainable Develop. 2018;5(2):42–53.

[82] Rasoanaivo P, Wright CW, Willcox ML, Gilbert B. Whole plant extracts versus single compounds for the treatment of malaria: synergy and positive interactions. Malar J. 2011;10 Suppl 1(Suppl 1):S4. doi: 10.1186/1475-2875-10-S1-S4 21411015 PMC3059462

[83] Rasheed I, Gruber R. Crude plant extracts and their anti-inflammatory potential in oral inflammatory cell models: a systematic review of in vitro studies. Int J Mol Sci. 2025;26(23):11253. doi: 10.3390/ijms262311253 41373414 PMC12692579

[84] Vaou N, Stavropoulou E, Voidarou C, Tsigalou C, Bezirtzoglou E. Towards advances in medicinal plant antimicrobial activity: a review study on challenges and future perspectives. Microorganisms. 2021;9(10):2041. doi: 10.3390/microorganisms9102041 34683362 PMC8541629

[85] Mary OG, Zaituni MS, Faith MP, Lughano KJM, Robinson MH, John OE. Antibacterial effects of single and combined crude extracts of Synadenium glaucescens and Commiphora swynnertonii. Afr J Infect Dis. 2022;16(2 Suppl):9–16. doi: 10.21010/Ajid.v16i2S.2 36124327 PMC9480890

[86] Mogana R, Adhikari A, Tzar MN, Ramliza R, Wiart C. Antibacterial activities of the extracts, fractions and isolated compounds from Canarium patentinervium Miq. against bacterial clinical isolates. BMC Complement Med Ther. 2020;20(1):55. doi: 10.1186/s12906-020-2837-5 32059725 PMC7076860

[87] Abedini A, Colin M, Hubert J, Charpentier E, Angelis A, Bounasri H, et al. Abundant extractable metabolites from temperate tree barks: the specific antimicrobial activity of prunus avium extracts. Antibiotics (Basel). 2020;9(3):111. doi: 10.3390/antibiotics9030111 32143394 PMC7148530

[88] Ishak A, Mazonakis N, Spernovasilis N, Akinosoglou K, Tsioutis C. Bactericidal versus bacteriostatic antibacterials: clinical significance, differences and synergistic potential in clinical practice. J Antimicrob Chemother. 2025;80(1):1–17. doi: 10.1093/jac/dkae380 39471409 PMC11695898

[89] Levison ME, Levison JH. Pharmacokinetics and pharmacodynamics of antibacterial agents. Infect Dis Clin North Am. 2009;23(4):791–815, vii. doi: 10.1016/j.idc.2009.06.008 19909885 PMC3675903

[90] Pankey GA, Sabath LD. Clinical relevance of bacteriostatic versus bactericidal mechanisms of action in the treatment of Gram-positive bacterial infections. Clin Infect Dis. 2004;38(6):864–70. doi: 10.1086/381972 14999632

[91] Seukep AJ, Tamambang FM, Matieta VY, Mbuntcha HG, Bomba FDT, Kuete V, et al. Potential of methanol extracts of Nephelium lappaceum (Sapindaceae) and Hyphaene thebaica (Arecaceae) as adjuvants to enhance the efficacy of antibiotics against critical class priority bacteria. PLoS One. 2025;20(2):e0314958. doi: 10.1371/journal.pone.0314958 39937773 PMC11819497

[92] Nigussie D, Davey G, Legesse BA, Fekadu A, Makonnen E. Antibacterial activity of methanol extracts of the leaves of three medicinal plants against selected bacteria isolated from wounds of lymphoedema patients. BMC Complem Med Therapies. 2021;21(1):2. 10.1186/s12906-020-03183-0 PMC7778819 33390165

[93] Sieberi BM, Omwenga GI, Wambua RK, Samoei JC, Ngugi MP. Screening of the Dichloromethane: Methanolic extract of centella asiatica for antibacterial activities against Salmonella typhi, Escherichia coli, Shigella sonnei, Bacillus subtilis, and Staphylococcus aureus. ScientificWorldJournal. 2020;2020:6378712. doi: 10.1155/2020/6378712 32694956 PMC7350070

[94] Manandhar S, Singh A, Varma A, Pandey S, Shrivastava N. High level of persister frequency in clinical staphylococcal isolates. BMC Microbiol. 2022;22(1):109. doi: 10.1186/s12866-022-02529-7 35448965 PMC10124895

[95] Morguette AEB, Bartolomeu-Gonçalves G, Andriani GM, Bertoncini GES, Castro IM de, Spoladori LF de A, et al. The antibacterial and wound healing properties of natural products: a review on plant species with therapeutic potential against Staphylococcus aureus wound infections. Plants (Basel). 2023;12(11):2147. doi: 10.3390/plants12112147 37299127 PMC10255540

[96] Chah KF, Eze CA, Emuelosi CE, Esimone CO. Antibacterial and wound healing properties of methanolic extracts of some Nigerian medicinal plants. J Ethnopharmacol. 2006;104(1–2):164–7. doi: 10.1016/j.jep.2005.08.070 16226414

[97] Breijyeh Z, Karaman R. Antibacterial activity of medicinal plants and their role in wound healing. Futur J Pharm Sci. 2024;10(1). doi: 10.1186/s43094-024-00634-0

[98] Patel JD, Shrivastava AK, Kumar V. Evaluation of some medicinal plants used in traditional wound healing preparations for antibacterial property against some pathogenic bacteria. J Clin Immunol Immunopathol Res. 2009;1(1):012–07.

[99] Maheshwaran L, Nadarajah L, Senadeera SPNN, Ranaweera CB, Chandana AK, Pathirana RN. Phytochemical testing methodologies and principles for preliminary screening/ qualitative testing. Asian Plant Res J. 2024;12(5):11–38. doi: 10.9734/aprj/2024/v12i5267

[100] Khan SU, Khan NH, Nahar L, Sarker SD, Htar TT. Unveiling the power of phytochemicals: virtual screening of phytochemicals. Computational phytochemistry. Elsevier; 2024. 413–37.

[101] Chowdhury SK, Misra D, Mandal V. Medicinal plant-derived antimicrobials’ fight against multidrug-resistant pathogens. Medicinal and aromatic plants: healthcare and industrial applications. 2021. 391–427.

[102] Sisay M, Bussa N, Gashaw T, Mengistu G. Investigating in vitro antibacterial activities of medicinal plants having folkloric repute in ethiopian traditional medicine. J Evid Based Integr Med. 2019;24:2515690X19886276. doi: 10.1177/2515690X19886276 31707813 PMC6851602

[103] Criollo-Mendoza MS, Contreras-Angulo LA, Leyva-López N, Gutiérrez-Grijalva EP, Jiménez-Ortega LA, Heredia JB. Wound healing properties of natural products: mechanisms of action. Molecules. 2023;28(2). doi: 10.3390/molecules28020598 36677659 PMC9867334

[104] Veiko AG, Olchowik-Grabarek E, Sekowski S, Roszkowska A, Lapshina EA, Dobrzynska I, et al. Antimicrobial activity of quercetin, naringenin and catechin: flavonoids inhibit staphylococcus aureus-induced hemolysis and modify membranes of bacteria and erythrocytes. Molecules. 2023;28(3):1252. doi: 10.3390/molecules28031252 36770917 PMC9920354

[105] Liu J, Chen X, Li S, Fang K, Sheng Y, Wang X, et al. Exploring the antimicrobial mechanisms of flavonoids with phenolic hydroxyl groups: implications for novel wound dressings. Industrial Crops and Products. 2026;239:122400. doi: 10.1016/j.indcrop.2025.122400

[106] Lu J, Fu X, Liu T, Zheng Y, Chen J, Luo F. Phenolic composition, antioxidant, antibacterial and anti-inflammatory activities of leaf and stem extracts from Cryptotaenia japonica Hassk. Industrial Crops and Products. 2018;122:522–32. doi: 10.1016/j.indcrop.2018.06.026

[107] Dev SK, Choudhury PK, Srivastava R, Sharma M. Antimicrobial, anti-inflammatory and wound healing activity of polyherbal formulation. Biomed Pharmacother. 2019;111:555–67. doi: 10.1016/j.biopha.2018.12.075 30597309

