# Supplementary material for: In vitro and in vivo antibacterial activities and phytochemical screening of 80% methanol extract from Ehretia cymosa leaves
Source: PLoS One. 2026 Jul 31;21(7):e0354982. doi: 10.1371/journal.pone.0354982 (PMC13427015; doi:10.1371/journal.pone.0354982)
Supplement: S1 Table — SO-simple ointment, NF-nitrofurazone, CEEC-Crude Extract E. cymosa, and LU- left untreated. (DOCX) [file pone.0354982.s001.docx]

**Supplementary Table 1. Graph 2 Data: Wound contraction in *S. aureus* infection**

|  | **Days post-infection** | | | | | | | | |
| --- | --- | --- | --- | --- | --- | --- | --- | --- | --- |
|  | **4** | **6** | **8** | **10** | **12** | **14** | **16** | **18** | **20** |
|  | **% of wound contraction** | | | | | | | | |
| **SO** | -6.82 | -1.4 | 5.21 | 11.58 | 24.84 | 39 | 53.69 | 72.11 | 86.98 |
| **0.2% NF** | -3.45 | 1.26 | 14.1 | 34.82 | 51.87 | 65.56 | 79.42 | 91.33 | 98.89 |
| **5% CEEC** | -5.47 | 2.58 | 6.47 | 17.73 | 37.82 | 51.87 | 61.49 | 83.31 | 94.42 |
| **10% CEEC** | -0.04 | 1.26 | 15.33 | 34.35 | 52.82 | 63.96 | 77.53 | 89.73 | 97.56 |
| **LU** | -9.67 | -2.71 | 3.26 | 9.09 | 21.36 | 32.73 | 46.18 | 67.76 | 83.96 |

**SO-simple ointment, NF-nitrofurazone, CEEC-Crude Extract *E. cymosa*, and LU- left untreated**
