# Supplementary material for: In vitro and in vivo antibacterial activities and phytochemical screening of 80% methanol extract from Ehretia cymosa leaves
Source: PLoS One. 2026 Jul 31;21(7):e0354982. doi: 10.1371/journal.pone.0354982 (PMC13427015; doi:10.1371/journal.pone.0354982)
Supplement: S2 Table — SO-simple ointment, NF-nitrofurazone, CEEC-Crude Extract E. cymosa, and LU- left untreated. (DOCX) [file pone.0354982.s002.docx]

**Supplementary Table 2. Graph 4 Data: wound contraction in *P.* *aeruginosa* infection**

|  | **Days post-infection** | | | | | | | | |
| --- | --- | --- | --- | --- | --- | --- | --- | --- | --- |
|  | **4** | **6** | **8** | **10** | **12** | **14** | **16** | **18** | **20** |
|  | **% of wound contraction** | | | | | | | | |
| **SO** | -12.42 | -7.65 | -1.43 | 6.47 | 17.64 | 29.2 | 42.96 | 62.27 | 82.33 |
| **0.2% NF** | -4.89 | -0.12 | 6.42 | 22.31 | 34.51 | 50.82 | 63.04 | 88.42 | 98.89 |
| **5% CEEC** | -10.98 | -6.87 | 1.18 | 11.49 | 23.58 | 40.13 | 53.64 | 70.64 | 90.56 |
| **10% CEEC** | -9.62 | -4.11 | 3.26 | 16.47 | 29.33 | 49.96 | 61.44 | 82.11 | 98.33 |
| **LU** | -15.27 | -8.31 | -2.14 | 5.20 | 14.02 | 25.8 | 41.04 | 59.8 | 80.49 |

**SO-simple ointment, NF-nitrofurazone, CEEC-Crude Extract *E. cymosa*, and LU- left untreated**
