# Supplementary material for: In vitro and in vivo antibacterial activities and phytochemical screening of 80% methanol extract from Ehretia cymosa leaves
Source: PLoS One. 2026 Jul 31;21(7):e0354982. doi: 10.1371/journal.pone.0354982 (PMC13427015; doi:10.1371/journal.pone.0354982)

PLOS ONE

In vitro and in vivo antibacterial activities of 80% methanol crude Ehretia cymosa leaf extract (Boraginaceae)

--Manuscript Draft--

**Manuscript Number:**	PONE-D-25-54833
**Article Type:**	Research Article
**Full Title:**	In vitro and in vivo antibacterial activities of 80% methanol crude Ehretia cymosa leaf extract (Boraginaceae)
**Short Title:**	antibacterial activities Ehretia cymosa leaf
**Corresponding Author:**	Tigist Gashaw<br>Haramaya University College of Health and Medical Sciences<br>Harar, ETHIOPIA
**Keywords:**	Antibacterial; Ehretia cymosa Thonn.; 80% methanol extract; wound infection
**Abstract:**	Antibiotic resistance has emerged as one of the most urgent global health threats, undermining the effective treatment of bacterial infections. In response, scientific interest is increasingly turning toward the discovery of natural and effective antimicrobial agents derived from medicinal plants. In Ethiopia, Ehretia cymosa (E. cymosa) is traditionally used to treat wound infections, fever, gastric ulcers, dysentery, and toothache. However, there is limited scientific evidence to support these traditional claims. Hence, the present study evaluated the in vitro and in vivo antibacterial activities of E. Cymose 80% methanol leaf extract. The air-dried and powdered leaves of E. cymosa were extracted via cold maceration with 80% methanol. The resulting crude extract was tested for antibacterial activity via the disk diffusion technique against common bacterial pathogens associated with infections. Additionally, an in vivo model of burn followed by infection was established in mice. One-way analysis of variance followed by Tukey's post hoc multiple comparison tests was used to compare the means of all parameters among and within groups. The leaves of E. cymosa demonstrated significant antibacterial activity ($p < 0.01$) against the tested bacterial strains in a dose-dependent manner. The minimum inhibitory concentration ranged from 6.25 to 75 mg/ml, while the minimum bactericidal concentration against Pseudomonas aeruginosa and Escherichia coli was 200 mg/ml. Moreover, the plant extract demonstrated greater effectiveness against S. aureus bacteria than against P. aeruginosa, as evidenced by faster wound contraction and a shorter epithelialization period than those of the controls. The 80% methanol extract of E. cymosa leaves exhibited notable antibacterial activity. These findings support its traditional use and highlight its potential as a source of natural antimicrobial agents. Further research is recommended to fractionate the extract and identify the specific bioactive compounds responsible for its antibacterial effects, which could contribute to future pharmacological developments.
**Order of Authors:**	Eshetu Mekonnen
	Dumessa Edessa
	Fitsum Weldegebreal
	Abenezer Aklog
	Tigist Gashaw
**Additional Information:**	
**Question**	**Response**
**Financial Disclosure**<br><br>Enter a financial disclosure statement that describes the sources of funding for the work included in this submission. Review	Yes

Powered by Editorial Manager® and ProduXion Manager® from Aries Systems Corporation

the submission guidelines for detailed requirements. View published research articles from *PLOS ONE* for specific examples.<br><br>This statement is required for submission and **will appear in the published article** if the submission is accepted. Please make sure it is accurate.<br><br>**Funded studies**<br>Enter a statement with the following details:<br>• Initials of the authors who received each award<br>• Grant numbers awarded to each author<br>• The full name of each funder<br>• URL of each funder website<br>• Did the sponsors or funders play any role in the study design, data collection and analysis, decision to publish, or preparation of the manuscript?<br><br>Did you receive funding for this work?	
Please add funding details.<br>as follow-up to "**Financial Disclosure**<br><br>Enter a financial disclosure statement that describes the sources of funding for the work included in this submission. Review the submission guidelines for detailed requirements. View published research articles from *PLOS ONE* for specific examples.<br><br>This statement is required for submission and **will appear in the published article** if the submission is accepted. Please make sure it is accurate.	Haramaya University

**Funded studies**<br>Enter a statement with the following details:<br>• Initials of the authors who received each award<br>• Grant numbers awarded to each author<br>• The full name of each funder<br>• URL of each funder website<br>• Did the sponsors or funders play any role in the study design, data collection and analysis, decision to publish, or preparation of the manuscript?<br><br>Did you receive funding for this work?"	
Please select the country of your main research funder (please select carefully as in some cases this is used in fee calculation).<br>as follow-up to "**Financial Disclosure**<br><br>Enter a financial disclosure statement that describes the sources of funding for the work included in this submission. Review the submission guidelines for detailed requirements. View published research articles from *PLOS ONE* for specific examples.<br><br>This statement is required for submission and **will appear in the published article** if the submission is accepted. Please make sure it is accurate.<br><br>**Funded studies**<br>Enter a statement with the following details:<br>• Initials of the authors who received each award<br>• Grant numbers awarded to each author<br>• The full name of each funder<br>• URL of each funder website<br>• Did the sponsors or funders play any role in the study design, data collection and analysis, decision to publish, or preparation of the manuscript?<br><br>Did you receive funding for this work?"	ETHIOPIA - ET
**Competing Interests**	The authors have declared that no competing interests exist.

Use the instructions below to enter a competing interest statement for this submission. On behalf of all authors, disclose any competing interests that could be perceived to bias this work—acknowledging all financial support and any other relevant financial or non-financial competing interests.<br><br>This statement is required for submission and **will appear in the published article** if the submission is accepted. Please make sure it is accurate and that any funding sources listed in your Funding Information later in the submission form are also declared in your Financial Disclosure statement.<br><br>View published research articles from *PLOS ONE* for specific examples.<br><br>**NO authors have competing interests**<br><br>Enter: *The authors have declared that no competing interests exist.*<br><br>**Authors with competing interests**<br><br>Enter competing interest details beginning with this statement:<br><br>*I have read the journal's policy and the authors of this manuscript have the following competing interests: [insert competing interests here]*<br><br>* typeset	
**Ethics Statement**<br><br>Enter an ethics statement for this submission. This statement is required if the study involved:<br><br>• Human participants<br>• Human specimens or tissue<br>• Vertebrate animals or cephalopods<br>• Vertebrate embryos or tissues	Ethical approval was obtained before the study from Haramaya University, specifically from the College of Veterinary Medicine, Animal Ethical Review Committee (Reference number: CVM/412/2024). Moreover, this study adhered to the guidelines outlined in the “Guide for the Care and Use of Laboratory Animals” by the National Research Council of the National Academies. All surgery was performed under anesthesia, and all efforts were made to minimize suffering.

Powered by Editorial Manager® and ProduXion Manager® from Aries Systems Corporation

• Field research<br><br>Write "N/A" if the submission does not require an ethics statement.<br><br>General guidance is provided below. Consult the submission guidelines for detailed instructions. **Make sure that all information entered here is included in the Methods section of the manuscript.**	

Powered by Editorial Manager® and ProduXion Manager® from Aries Systems Corporation

**Format for specific study types**<br><br>**Human Subject Research (involving human participants and/or tissue)**<br>• Give the name of the institutional review board or ethics committee that approved the study<br>• Include the approval number and/or a statement indicating approval of this research<br>• Indicate the form of consent obtained (written/oral) or the reason that consent was not obtained (e.g. the data were analyzed anonymously)<br><br>**Animal Research (involving vertebrate animals, embryos or tissues)**<br>• Provide the name of the Institutional Animal Care and Use Committee (IACUC) or other relevant ethics board that reviewed the study protocol, and indicate whether they approved this research or granted a formal waiver of ethical approval<br>• Include an approval number if one was obtained<br>• If the study involved *non-human primates*, add *additional details* about animal welfare and steps taken to ameliorate suffering<br>• If anesthesia, euthanasia, or any kind of animal sacrifice is part of the study, include briefly which substances and/or methods were applied<br><br>**Field Research**<br><br>Include the following details if this study involves the collection of plant, animal, or other materials from a natural setting:<br>• Field permit number<br>• Name of the institution or relevant body that granted permission	
**Data Availability**<br><br>Authors are required to make all data underlying the findings described fully available, without restriction, and from the time of publication. PLOS allows rare exceptions to address legal and ethical concerns. See the PLOS Data Policy and FAQ for detailed information.	Yes - all data are fully available without restriction; Yes - all data are fully available without restriction

Powered by Editorial Manager® and ProduXion Manager® from Aries Systems Corporation

A Data Availability Statement describing where the data can be found is required at submission. Your answers to this question constitute the Data Availability Statement and **will be published in the article**, if accepted.<br><br>**Important:** Stating 'data available on request from the author' is not sufficient. If your data are only available upon request, select 'No' for the first question and explain your exceptional situation in the text box.<br><br>Do the authors confirm that all data underlying the findings described in their manuscript are fully available without restriction?	
**Describe where the data may be found in full sentences. If you are copying our sample text, replace any instances of XXX with the appropriate details.**<br><br>• If the data are **held or will be held in a public repository**, include URLs, accession numbers or DOIs. If this information will only be available after acceptance, indicate this by ticking the box below. For example: *All XXX files are available from the XXX database (accession number(s) XXX, XXX.).*<br>• If the data are all contained **within the manuscript and/or Supporting Information files**, enter the following: *All relevant data are within the manuscript and its Supporting Information files.*<br>• If neither of these applies but you are able to provide **details of access elsewhere**, with or without limitations, please do so. For example:<br><br>*Data cannot be shared publicly because of [XXX]. Data are available from the XXX Institutional Data Access / Ethics Committee (contact via XXX) for researchers who meet the criteria for access to confidential data.*<br><br>*The data underlying the results presented in the study are available from (include the name of the third party*	All relevant data are within the manuscript and its Supporting Information files.All relevant data are within the manuscript and its Supporting Information files.

and contact information or URL).<br>• This text is appropriate if the data are owned by a third party and authors do not have permission to share the data.<br><br>* typeset	
Additional data availability information:	;

Powered by Editorial Manager® and ProduXion Manager® from Aries Systems Corporation

Click here to access/download;Manuscript;Manuscript PLOSone.docx

In vitro and in vivo antibacterial activities of 80% methanol crude Ehretia cymosa leaf extract (Boraginaceae)

Eshetu Mekonnen[1], Dumessa Edessa[2], Fitsum Weldegebreal[3,4], Abenezer Aklog[5], Tigist Gashaw[5*]

Authors' affiliation

[1]Haramaya University, College of Health and Medical Sciences, Harar, Ethiopia

[2]Clinical Pharmacy Department, School of Pharmacy, College of Health and Medical Sciences, Haramaya University, Harar, Ethiopia

[3] School of Medical Laboratory Sciences, College of Health and Medical Sciences, Haramaya University, Ethiopia

[4]Laboratory Bacteriology Research, Department of Diagnostic Sciences, Faculty of Medicine and Health Sciences, Ghent University, Ghent, Belgium

[5]Pharmacology Department, School of Pharmacy, College of Health and Medical Sciences, Haramaya University, Harar, Ethiopia

Corresponding author: Email: yekalabel@gmail.com (TG)

Abstract

Antibiotic resistance has emerged as one of the most urgent global health threats, undermining the effective treatment of bacterial infections. In response, scientific interest is increasingly turning toward the discovery of natural and effective antimicrobial agents derived from medicinal plants. In Ethiopia, *Ehretia cymosa (E. cymosa)* is traditionally used to treat wound infections, fever, gastric ulcers, dysentery, and toothache. However, there is limited scientific evidence to support these traditional claims. Hence, the present study evaluated the in vitro and in vivo antibacterial activities of E. Cymose 80% methanol leaf extract. The air-dried and powdered leaves of *E. cymosa* were extracted via cold maceration with 80% methanol. The resulting crude extract was tested for antibacterial activity via the disk diffusion technique against common bacterial pathogens associated with infections. Additionally, an in vivo model of burn followed by infection was established in mice. One-way analysis of variance followed by Tukey's post hoc multiple comparison tests was used to compare the means of all parameters among and within groups. The leaves of *E. cymosa* demonstrated significant antibacterial activity ($p < 0.01$) against the tested bacterial strains in a dose-dependent manner. The minimum inhibitory concentration ranged from 6.25 to 75 mg/ml, while the minimum bactericidal concentration against *Pseudomonas aeruginosa* and *Escherichia coli* was 200 mg/ml. Moreover, the plant extract demonstrated greater effectiveness against *S. aureus* bacteria than against *P. aeruginosa,* as evidenced by faster wound contraction and a shorter epithelialization period than those of the controls. The 80% methanol extract of *E. cymosa* leaves exhibited notable antibacterial activity. These findings support its traditional use and highlight its potential as a source of natural antimicrobial agents. Further research is recommended to fractionate the extract and identify the specific bioactive compounds

responsible for its antibacterial effects, which could contribute to future pharmacological developments.

Keywords: Antibacterial, *Ehretia cymosa Thonn.,* 80% methanol extract, wound infection

Introduction

The discovery, development, and therapeutic application of antibiotics in the nineteenth century have significantly decreased the public health risks posed by bacterial infections (1, 2). However, the misuse and overuse of these drugs have led to a troubling rise in bacterial resistance to current chemotherapeutic agents. Additionally, antibiotics can sometimes cause adverse effects, including allergic reactions, immunosuppression, and hypersensitivity (3, 4). These challenges highlight the urgent need to intensify efforts toward developing new antibacterial agents that are effective against resistant pathogenic microorganisms (5).

One promising approach to combat the growing issue of antibiotic resistance is the systematic extraction and screening of bioactive compounds from traditional medicinal plants. This strategy aims to discover new, effective substances with strong antimicrobial properties (6, 7). The demand for plant-based treatments is growing in both developed and developing countries, driven by the perception that these remedies are natural, safe, affordable, and generally associated with fewer side effects (8, 9). Medicinal plants have shown antibacterial activity against various pathogens responsible for infections, especially wounds, including *S. aureus, P. aeruginosa,* and *Streptococcus* species (10, 11). An astounding array of antibacterial plants has been found worldwide, where larger populations reside in lower- and middle-income countries that are dependent on traditional plants (10, 12-14).

Ethiopia is home to a diverse range of plant species, with approximately 12% being endemic, making the country a valuable source of medicinal plants with potential health benefits. *Ehretia cymosa* (*E. cymosa*) is a native plant that is widely distributed across Ethiopia (Fig. 1). It is locally known as the “Game” in Amharic and “Hulaagaa, Ulaagaa, Garmi” in Afan Oromo. Experimental studies have shown that various extracts and solvent fractions of *E. cymosa* exhibit a range of biological activities, including antioxidant, antihyperglycemic, antidiarrheal, paralysis-relieving, antiepileptic, and antimicrobial effects (11). It is traditionally used to treat gastrointestinal issues, bone fractures, and skin infections in eastern China. The plant leaves are prepared as a poultice, a soft, moist mass of plant material applied directly to wounds. To make a poultice, fresh leaves are mashed with cold water and then reduced in size, or dried leaves are boiled in water and cooled. There is some evidence of its antibacterial activity against a limited number of bacterial strains. Therefore, the present study further tested it against additional bacterial strains, considering parameters such as the minimum bactericidal concentration (MBC), which helps explore the relationship between the minimum inhibitory concentration (MIC). Both in vitro and in vivo models have been used, as these methods complement each other, each providing unique and essential information for a broader understanding of a treatment or biological process. Accordingly, this study evaluated the in vitro and in vivo antibacterial activities of *E. cymosa* 80% methanol leaf extract.

Fig 1. Leaves of Ehretia cymosa Thonn.

Methods and materials

Chemicals and instruments

Chemicals

All solvents used for the extraction process were of laboratory (analytical) grade. Drugs and chemicals, including methanol absolute (LOBA Chemie Pvt. Ltd., India), and distilled water was used for the extraction of the plant material. A simple ointment base was prepared using the following ingredients: wool fat, hard paraffin, and cecostearyl alcohol (all from BDH Chemicals Ltd., England), along with white soft paraffin (Anonchem Ltd., China). The additional chemicals used included Mueller Hinton agar, normal saline, and Nitrofurazone ointment USP 0.2% (Galentic Pharm, Pvt. Ltd. Co., India), ketamine hydrochloride injection USP (Neon Laboratories Ltd., India), ciprofloxacin 5 µg standard antibiotic disc (OXOID LIMITED, Basingstoke, Hampshire, England), and EDTA.

Instruments

The following apparatuses and instruments were used during the experiment: a digital weighing balance (Mettler Toledo, Switzerland), a mini orbital shaker (Bibby Scientific Limited, UK), a rotary evaporator (Buchii model R200, Switzerland), a steam sterilizer, an incubator, a petri dish, a micropipette, a paper puncher, a water bath, a separatory funnel, an Erlenmeyer conical flask, a measuring cylinder, a falcon, a shaver with a sterile blade (Razor), a ruler, a mortar and pestle, an ointment slab, a syringe with needles, surgical and disposable gloves, surgical threads with curved needles, forceps, whatman filter paper (Number 1, Whatman Ltd., England), a permanent marker, and graph paper.

Collection, authentication, and extraction of E. cymosa leaves

The plant material was collected from the eastern part of Ethiopia, identified, and authenticated by a biologist. A voucher specimen, labeled HUHE0000002255 (023551), was deposited in the Herbarium of the Biology Department, Faculty of Natural and Computational Science at Haramaya University.

Fresh leaves of *E. cymosa* were collected and thoroughly washed with tap water to remove surface dirt. The cleaned leaves were then shade-dried and ground into a fine powder. Extraction was carried out via a cold maceration technique, with 80% (v/v) methanol selected as the solvent for its high polarity, efficiency, compatibility with analytical techniques, and proven effectiveness in previous studies. Although some solvents may offer higher potency, 80% methanol provides a balanced approach in terms of safety, solubility, and extraction yield. A total of 600 grams of the powdered plant material was weighed via an electronic digital balance and soaked with 300 ml of 80% methanol in Erlenmeyer conical flasks. The flasks were then placed on mini orbital shakers and adjusted at 120 revolutions per minute (rpm), with periodic agitation for 72 hours. The mixture was first filtered through a nylon cloth, and the residue was macerated twice more to maximize the extraction yield. The filtrate was filtered again using Whatman filter paper (number 1) with a pressurized suction system. The total filtrate was concentrated via a low-pressure rotary evaporator set at 121 mbar, 45 rpm, and 40°C. The resulting concentrate was stored in a deep freezer and subsequently freeze-dried via a lyophilizer at -20°C under vacuum pressure (200 mBar), yielding a brownish crude extract.

Microbial organisms

Standard strains (American Type Culture Collections (ATCC)) of the gram-positive bacterial species *Staphylococcus aureus (ATCC 25923) and Streptococcus pyogenes (NCTC 12696),* and the gram-negative bacteria *Escherichia coli (ATCC 25922), Pseudomonas aeruginosa (ATCC27853),* and *Klebsiella pneumoniae* were obtained from Eastern Hararghe Health Research, Haramaya University, Harar.

Preparation of experimental animals

Healthy Swiss albino adult mice of both sexes weighing 20–30 g and aged 6–8 weeks were obtained from the National Veterinary Institute (NVI), Bishoftu, Ethiopia, and used throughout the experiment. The animals were housed in plastic cages under standard conditions, with a 12-hour light/dark cycle and ad libitum access to standard food and water. They were allowed to acclimate to laboratory conditions for one week before beginning any experiments. Throughout the study, the animals were handled in accordance with international guidelines for the use and care of experimental animals (15, 16). At the conclusion of the experiment, the animals were euthanized with the recommended dose of ketamine.

E. cymosa leaves Ointment formulation

A simple ointment base was prepared according to the British Pharmacopeia (17). The indicated ointment preparations, 200 g each with two strengths (5% w/w and 10% w/w) and a simple ointment without an active substance (as a control), were also formulated via a reduced formula (Table 1). To prepare an ointment, a calculated amount of hard paraffin and cetostearyl alcohol was combined and heated in a beaker in a water bath. In a separate beaker, the wool, fat, and white soft paraffin mixture was heated while stirring to ensure homogeneity. After being removed from

the water bath, the former was combined with the latter and mixed until cold. A medicated ointment with a homogeneous consistency and smooth texture was produced. To make 5% ointment, 10 g of extract was weighed and combined with 190 g of simple ointment base. Similarly, 20 g of extract was combined with 180 g of ointment base. The prepared ointments were 5% and 10% crude extract, and a nonmedicated ointment base was used as a negative control. This preparation took place at five-day intervals until all the experiments were completed.

Table 1: Master and reduced formulas used for formulating a simple ointment

Ingredients	Master Formula	Reduced Formula
Wool fat	50 g	10 g
Hard paraffin	50 g	10 g
Ceto-stearyl alcohol	50 g	10 g
White soft Paraffin	850 g	170 g
Qs	1000 g	200 g

In vitro antibacterial activity testing

In vitro, *the* antibacterial activity of the crude extract was evaluated against all the aforementioned bacterial strains. In vivo antibacterial activity was tested using *P. aeruginosa* and *S. aureus.*

Agar disc diffusion

The agar disc diffusion method was used to determine the antibacterial activity (zone of inhibition) of the plant extract *E. cymosa* (18, 19). The five bacterial strains were activated on their selective media**,** MacConkey agar for *K. pneumoniae* and mannitol–salt agar for *S. aureus*, and were incubated at 37°C for 24 hours**.** Muller Hinton agar (MHA) was prepared according to the manufacturer's recommendation (39 g to be diluted in 1000 ml of distilled water). The powder

media was added to a flask containing the measured volume of distilled water and placed on a hot plate with occasional shaking until it boiled. The media were subsequently sterilized at 121°C for 15 minutes via an autoclave. The medium was cooled to 45–50°C inside a water bath. For fastidious *Streptococcus* species (*S. pyogenes*) that require enriched media, 5% sterile sheep blood was added to the MHA medium. After that, 20 ml of media was poured into prelabeled sterile Petri dishes (90 mm) aseptically and allowed to solidify (20-22). A small number of bacterial colonies of each strain from the agar plate were transferred with a sterile inoculating loop to nutrient broth until the turbidity was adjusted to a density of 10^8 cells ml^{-1} of the McFarland 0.5 turbidity standard (23). The culture was subsequently spread evenly (with 60° rotations of Petri dishes to achieve uniform bacterial growth) onto MHA via sterile cotton swabs following aseptic conditions in a safety cabinet, and the medium was allowed to dry for 5 minutes at room temperature.

The external surface of each plate was divided into *five parts* and labeled with a permanent marker, as each contained five paper discs: three discs containing extracts at different concentrations, one from the positive control, and the remainder from the negative control. Paper disks were punched out from a sheet of absorbent filter paper (Whatman No. 1; 6 mm in diameter) by an ordinary office two-hole puncher. These were dispensed in batches in screw-capped bottles and sterilized at 121°C for 1 hr. A test sample was prepared at three concentrations, 300 mg/ml, 200 mg/ml, and 100 mg/ml, using 10% DMSO as the solvent. The concentrations were selected on the basis of a combination of factors, including prior toxicity studies that established the extract's safety up to 2000 mg/kg and the need to assess dose-dependent efficacy within a safe range. Previous research at 25 mg/ml revealed initial antibacterial potential, but these higher concentrations aimed to explore whether increased doses yield stronger antibacterial effects. Testing across this range allows us to capture dose–response relationships that could identify an optimal therapeutic

concentration. Additionally, 10% DMSO was used as a solvent because of its effective solubilizing properties and low cytotoxicity, ensuring reliable in vitro results.

The sterilized filter paper discs soaked in different beakers containing the dissolved solvent extracts of different concentrations and the negative control (DMSO) were removed with sterilized forceps, air-dried, and placed on plates with the different test organisms. A 5µg ciprofloxacin disc was used as a positive control. The plates were then left at room temperature for two hours to allow diffusion of the test samples and incubated at 37°C for 24 hours. After an incubation period, the plates were observed for the inhibition zone (zones showing no bacterial growth). Inhibition zones were measured in millimeters (mm) via a scale, and the tests were performed in triplicate (20, 21).

Determination of the minimum inhibitory concentration (MIC)

For the concentration that resulted in a zone of inhibition greater than or equal to 7 mm for the tested organism, the MIC was determined via the broth macro-dilution method (20). The extract, which showed activity at 300 mg/ml, was further diluted to a 1:2 ratio to obtain 150 mg/ml, 125 mg/ml, and 75 mg/ml (1:4). The concentration that showed activity at 100 mg/ml was diluted to 50 mg/ml (1:2), 25 mg (1:4), 12.5 mg/ml (1:8) and 6.25 mg/ml (1:16). Future studies could explore lower concentrations to refine the MIC. Broth media was prepared according to the manufacturer's recommendation. Inoculum preparation and standardization were performed aseptically as described in section 3.6.2. Each concentration of the extract was diluted in a 1:2 ratio with the broth media, and 1 mL of each mixture was added to a standard test tube. The standard inocula were diluted in broth media (1:150) (24, 25). One milliliter of the diluent was added to each tube, which contained different concentrations of the extract. A tube without any test sample was used as a control for bacterial growth. The tubes were covered and incubated at 37°C for 24 hours. After

incubation, bacterial growth was analyzed visually; the minimum concentration that did not result in any visible bacterial growth was taken as the MIC (20, 26).

Determination of the minimum bactericidal concentration (MBC)

MBC was determined by subculturing the extract with a value greater than or equal to the MIC value. The contents of the MIC test solutions were streaked on an agar plate via sterile cotton swabs and incubated at 37°C for 24 hours. The lowest concentration that yielded no single bacterial colony was taken as the MBC (26).

In vivo antibacterial activity in the infected wound model

The burn model followed by the infection model is crucial for understanding and improving burn wound treatment because it closely replicates the clinical scenario of burn injuries, which are highly susceptible to infection. This model allows researchers to study the complex interplay between burn injury, infection, and the body's healing response, ultimately leading to the development of more effective therapies (27).

The animals were randomly grouped into ten groups, five mice in each group. There were five interventions with two infecting bacterial strains in each category: the control (no treatment), negative control (receiving placebo ointment), positive control (0.2% nitrofurazone ointment), 5% *E. cymosa* ointment-treated, and 10% *E. cymosa* ointment-treated groups. The mice were burned and infected with *P. aeruginosa* and *S. aureus* at a sublethal dose of 1×10^7 CFU/ml/mouse in each intervention category. First, the mice were anesthetized, shaved, and cleaned. A metal mold with the dimensions selected to provide the desired size of burn in an animal of known weight. The upper limit of burn size permitted by this approach is approximately 30% of the TBSA, which was calculated via Meeh's formula (28, 29), and the mean burn area for each group was 28.26 mm^2. To make the wound, the metal mold was heated via a Bunsen burner. The temperature of the

heated metal was determined to be 100°C. Then, the metal mold was pressed on the back of the mouse; to avoid using too heavy a force to damage the mouse skin, 10 seconds later, the metal mold was picked up, and cold water was sprayed for cooling.

To avoid hypothermia and shock, the mice were placed on an electric heating pad following the burn. Such an injury is not fatal, but it results in a third-degree (full-thickness) burn. Then, fluid replacement therapy in the form of a subcutaneous injection of 0.8 ml of 0.9% NaCl solution was initiated. Mice were infected with *P. aeruginosa* and *S. aureus* at sublethal doses of 1 × 107 CFU/ml/mouse from a preprepared McFarland 0.5 turbidity standard, according to their groups. After recovery, the mice were returned to their cages and fed regularly. Starting 72 hours after wounding (day 3), the animals were treated with the corresponding ointment as specified in the grouping and dosing section, except for the last group of animals, which were left untreated and used as benchmarks (untreated negative control). Untreated wounds were exposed to open air, and the animals were housed in separate cages. The progress of healing lesions, restoring normal skin structure with topical wound therapy, and growing hair on repaired skin was documented.

Meeh's Formula

$$TBSA = kW^{2/3} \quad \text{.........} \quad (1)$$

where TBSA is the total body surface area, k is Meeh's constant (9.83), and W is the weight of the mice in g (30).

Statistical analysis

The data are presented as the mean ± standard error of the mean (M ± SEM). One-way ANOVA followed by Tukey's post hoc multiple comparison tests was used to compare the means of all parameters among and within groups. A p-value < 0.05 was considered statistically significant. SPSS version 27 software was used to perform the statistical analysis.

Ethical clearance

Ethical approval was obtained before the study from Haramaya University, specifically from the College of Veterinary Medicine, Animal Ethical Review Committee (Reference number: CVM/412/2024). Moreover, this study adhered to the guidelines outlined in the "Guide for the Care and Use of Laboratory Animals" by the National Research Council of the National Academies. All surgeries were performed under anesthesia, and all efforts were made to minimize suffering.

Results

In vitro *antibacterial activity*

Bacterial growth inhibition

Using the agar disc diffusion method, the 80% methanol extract of *E. cymosa* leaves demonstrated statistically significant antibacterial activity compared with the negative control (DMSO) against five standard bacterial strains in a concentration-dependent manner. The zones of inhibition ranged from 7.17 mm (against *S. pyogens*) to 22.5 mm (against *S. aureus)* in diameter. The extract had the greatest growth inhibitory effect on the gram-positive bacterium *S. aureus*, followed by the gram-negative bacteria *P. aeruginosa, E. coli, and K. pneumoniae*. Among the tested strains, *S. pyogenes* was the least sensitive to the extract across all concentrations. The positive control (ciprofloxacin) consistently showed significant differences (ranging from $p < 0.05$ to $p<0.001$) compared with all extract concentrations and the negative control against all bacterial strains, as presented in Table 2.

Table 2: Zone of inhibition of the 80% methanol extracts of *E. cymosa* leaves at various concentrations and the standard ciprofloxacin against the tested bacterial strains

Concentration (mg/ml)	Zone of inhibition (mm) mean ± SEM				
	Gram +Ve bacteria		Gram –Ve bacteria		
	S. aureus	***S. pyogens***	***P. aeruginosa***	***E. coli***	***K. pneumoniae***
100 mg/ml	17 ± 0.58^{e***}	7.17± 0.17^{e***}	11.67± 0.67^{e***}	11 ± 2.08^{e**}	8.67± 0.73^{e***}
200 mg/ml	21.17 ± 1.26 b*e***	9.00± 0.58 b*e***	15.00 ± 0.58 b*e***	14.00 ± 3.00^{e**}	11.17± 0.93^{e***}
300 mg/ml	22.5 ± 0.87 *b**e****	11.33± 0.88 b**e***	16.67 ± 0.67 *b*** e***	15.67 ± 3.18^{e**}	12.33± 0.88 b*e***
5 µg (Cip)	26.33 ± 0.88 be***cd*	25.00± 0.33 bcde***	25.00 ± 1 bce***d**	35.00 ± 2.08 bcd**e***	21.00± 0.58 bce***d**
DMSO	NA	NA	NA	NA	NA

Note: Values are expressed as the means ± SEM (n = 3). Analysis was performed with one-way analysis of variance followed by Tukey's post hoc test. **Abbreviations:** +Ve, positive; –Ve, negative; Cip, ciprofloxacin; DMSO, dimethyl sulfide; NA, no activity/inhibition at the tested dose(s); *a* compared with the positive control (ciprofloxacin); *b* compared with 100 mg/ml; *c* compared with 200 mg/ml; *d* compared with 300 mg/ml; and e compared with the negative control (DMSO). *P < 0.05; **P < 0.01; ***P <0.001.

Minimum inhibitory concentration and minimum bactericidal concentration

To quantitatively assess the antibacterial activity of the 80% methanol extract of *E. cymosa* leaves, the MIC and MBC cutoff values were determined for each concentration. These values were obtained for bacterial strains that inhibited growth in the agar disc diffusion assay. The lowest MIC (6.25 mg/ml) was recorded for *P. aeruginosa*. The lowest MBC (200 mg/ml) was observed against both *P. aeruginosa* and *E. coli.* No MBC values were observed for *S. aureus, K. pneumoniae*, or *S. pyogenes* within the tested concentration range (Tables 3 and 4).

Table 3: MICs of the 80% methanol extract of the leaves of *E. cymosa* in broth macro dilution method

Bacterial strains	MIC	
	Tested Concentrations of *E.cymosa* extract in mg/ml	
	(150, 125, 100, 75, 50, 25, 12.5, and 6.25)	
Gram +Ve	*S. aureus*	50.00 ± 0.00
	S. pyogen	75.00 ± 0.00
Gram –Ve	*P. aeruginosa*	6.25.00 ± 0.00
	E. coli	50.00 ± 0.00
	K. pneumonia	75.00 ± 0.00

Note: Values are expressed as the mean ± SEM (n = 3); analysis was performed with one-way analysis of variance followed by Tukey's post hoc test. Abbreviations: MIC, minimum inhibitory concentration

Table 4: MBC of the 80% methanol extract of the leaves of *E. cymosa* in broth macro dilution method

Bacterial strains	MBC	
	Tested Concentrations of *E.cymosa* extract in mg/ml	
	(300, 200, 150,125, 100, 75, 50, 25, 12.5, and 6.25)	
Gram +Ve	*S. aureus*	
	S. pyogen	
Gram –Ve	*P. aeruginosa*	200.00 ± 0.00
	E. coli	200.00 ± 0.00
	K. pneumonia	

Note: Values are expressed as the mean ± SEM (n = 3); analysis was performed with one-way analysis of variance followed by Tukey's post hoc test. Abbreviations: MBC, minimum bactericidal concentration.

In vivo *antibacterial activities*

Wound Contraction in S. aureus-infected Mice

For the first few days, the contraction process was negative, which might be due to infection and damage to the tissues by the burn. Compared with the negative SO-treated, untreated, and 5%

crude extract ointment preparation-treated groups, the 10% (w/w) crude extract ointment-treated group presented significant ($p < 0.01$, $p < 0.01$, and $p < 0.05$) wound contraction starting from the 8th day post-burn wounding and *S. aureus* infection. Compared with the negative control group and the SO-treated group, the 5% (w/w) extract ointment-treated group presented significant ($p < 0.05$) wound contraction starting from the 10th and 12th days post-burn. In addition, the groups treated with the standard drug (0.2% NF) were significantly different from the negative SO ($p < 0.05$) and untreated groups ($p < 0.01$) on the 8th day post-wounding. Additionally, $P < 0.01$ was significant starting on the 10th day after wounding and infection compared with the 5% (w/w) crude extract ointment-treated group. Compared with the left untreated group, the SO-treated group showed significant ($P < 0.05$) wound contraction on days 18 and 20 post-wounding and *S. aureus* infection. The maximum rate of wound contraction for both the 10% (w/w) crude extract ointment of the leaves of *E. cymosa* and the 0.2% nitrofurazone (w/w) ointment was observed from the 10th day onward ($p < 0.001$) compared with the negative SO-treated control and the left untreated groups (Table 5) (Fig. 2).

Fig 2. Burn wounds followed by the healing of *S. aureus*-infected mice: CEEC-Crude Extract E. Cymosa, NF-nitrofurazone, SO-simple ointment, and LU- left untreated

Table 5: Effects of 80% methanol extract of the leaves of *E. cymosa* on *S. aureus-infected* wounds in mice

Days	Wound area (mm^2) ± SEM (% contraction) *S. aureus-infection*				
	**SO**	**5% CEEC**	**10% CEEC**	**0.2% NF**	LU
4	30.19±0.62 (-6.82)	29.81± 0.73 (-5.47)	28.27±0.5958 (-0.04)	29.24±0.8645 (-3.45)	30.99±1.01 (-9.67)
6	28.66±0.71 (-1.4)	27.53 ±0.75 (2.58)	27.91±0.76 (1.26)	27.91±0.76 (1.26)	29.03±0.47 (-2.71)
8	26.79±0.62 (5.21)	26.43±0.81 (6.47)	23.93±0.42 (15.33) $^{a** c*e**}$	24.285±0.66 (14.1) ^{a*e**}	27.34±0.58 (3.26)
10	24.99±1.48 (11.58)	23.25±0.64 (17.73) ^{e*}	18.55±0.46 (34.35) ^{a***c***e***}	18.42±0.76 (34.82) ^{a***c**e***}	25.69±0.44 (9.09)
12	21.24±0.52 (24.84)	17.57±1.22 (37.82) ^{a*e**}	13.33±0.32 (52.82) ^{a***c**e***}	13.60±0.49 (51.87) ^{a***c*e***}	22.23±0.41 (21.36)
14	17.24±0.88 (39)	13.60±0.49 (51.87) ^{a**e***}	10.19±0.36 (63.96) ^{a***c***e***}	9.73±0.27 (65.56) ^{a***c***e***}	19.01±0.38 (32.73)
16	13.09±0.50 (53.69) ^{e*}	10.88±0.48 (61.49) ^{a*e***}	6.35±0.33 (77.53) ^{a***c***e***}	5.82±0.21 (79.42) ^{a***c***e***}	15.21±0.44 (46.18)
18	7.88±0.57 (72.11)	4.72±0.42 (83.31) ^{a**e***}	2.90±0.15 (89.73) ^{a***c**e***}	2.45±0.21 (91.33) ^{a***c**e***}	9.11±0.57 (67.76)
20	3.68±0.26 (86.98) ^{e*}	1.58±0.22 (94.42) ^{a***e***}	0.69±0.31 (97.56) ^{a***c*e***}	0.31±0.19 (98.89) ^{a***c**e***}	4.53±0.24 (83.96)

Note: Values are expressed as the mean ± SEM (n = 5); n = 5 mice in each group. Analysis was performed with one-way analysis of variance followed by Tukey's post hoc test: *a* compared with the negative control (SO), *b* compared with the positive control (NF), *c* compared with 5% CEEC, d compared with 10% CEEC, and e compared with the untreated negative control. *P < 0.05; **P < 0.01; ***P < 0.001. Abbreviations: CEEC, crude extract of *Ehretia cymosa*; SO, simple ointment base (negative control); NF, nitrofurazone; LU, left untreated.

Wound Contraction in P. aeruginosa-infected Mice

Compared with the negative control (SO)-treated and untreated groups, the 10% (w/w) crude extract ointment-treated group showed significant ($p < 0.05$) wound contraction starting from the 10th day post-burn wounding and *P. aeruginosa* infection. Compared with the 5% (w/w) extract-treated group, the 10% extract-treated group exhibited significant ($p < 0.01$) wound contraction from day 16 onward. However, the 5% (w/w) extract-treated group showed significant ($p < 0.05$) wound contraction starting from the 14th day post-wounding and infection compared with the negative, untreated control, and SO-treated groups. Compared with the negative control SO-treated and untreated groups, the group treated with the standard drug (0.2% NF) presented significant ($p < 0.05$, $p < 0.01$) wound contraction beginning from the 10th day after wounding and infection. Additionally, significant differences ($p < 0.01$) were detected from the 16th day onward compared with the 5% (w/w) crude extract ointment-treated group. Compared with the untreated control group, the SO-treated groups presented no significant difference in wound contraction post-wounding or *P. aeruginosa* infection. There was no significant difference between the 10% crude extract ointment preparation and the standard drug. The maximum rate of wound contraction for the 10% (w/w) ointment and the 0.2% nitrofurazone (w/w) ointment was observed from the 16th day onward ($p < 0.001$) compared with the untreated groups, whereas from day 18 onward compared with the SO-treated negative control (Table 6) and (Fig. 3).

Fig 3. Burn wounds followed by the healing of *P. aeruginosa*-infected mice: CEEC-Crude Extract E. Cymosa, NF-nitrofurazone, SO-simple ointment, and LU- left untreated

Table 6: Effects of 80% methanol extract of the leaves of *E. cymosa* on *P. aeruginosa-infected* wounds in mice

Days	Wound area (mm^2) ± SEM (% contraction) *P. aeruginosa-infection*				
	**SO**	**5% CEEC**	**10% CEEC**	**0.2% NF**	LU
4	31.77±0.75 (-12.42)	31.36± 0.48 (-10.98)	30.98±0.78 (-9.62)	29.64±1.23 (-4.89)	32.57±0.75 (-15.27)
6	30.42±1.26 (-7.65)	30.20±0.87 (-6.87)	29.42±0.76 (-4.11)	28.29±1.01 (-0.12)	30.61±1.15 (-8.31)
8	28.67±0.88 (-1.43)	27.93±1.08 (1.18)	27.34±0.58 (3.26)	26.45±1.01 (6.42)	28.86±1.03 (-2.14)
10	26.43±0.81 (6.47)	25.01±1.04 (11.49)	23.61±0.88 (16.47) ^{a*e*}	21.95±1.27 (22.31) ^{a*e**}	26.79±0.68 (5.20)
12	23.27±1.00 (17.64)	21.60±0.97 (23.58)	19.97±0.79 (29.33) ^{a*e**}	18.51±1.50 (34.51) ^{a*e*}	24.30±0.86 (14.02)
14	20.01±1.18 (29.20)	16.92±0.54 (40.13) ^{a*e*}	14.14±0.67 (49.96) ^{a**e**}	13.90±0.85 (50.82) ^{a**e**}	20.97±1.20 (25.80)
16	16.12±1.17 (42.96)	13.10±0.66 (53.64) ^{a*e*}	10.90±0.59 (61.44) ^{a**c**e***}	10.44±0.67 (63.04) ^{a**c**e***}	16.66±0.928 (41.04)
18	10.66±0.59 (62.27)	8.30±0.67 (70.64) ^{a*e**}	5.06±0.57 (82.11) ^{a***c**e***}	3.27±0.13 (88.42) ^{a***c***e***}	11.36±0.53 (59.8)
20	4.99±0.19 (82.33)	2.67±0.47 (90.56) ^{a*e**}	0.47±0.19 (98.33) ^{a***c**e***}	0.31±0.19 (98.89) ^{a***c**e***}	5.51±0.49 (80.49)

Note: Values are expressed as the mean ± SEM (n = 5); n = 5 mice in each group. Analysis was performed with one-way analysis of variance followed by Tukey's post hoc test: *a* compared with the negative control (SO), *b* compared with the positive control (NF), *c* compared with 5% CEEC, d compared with 10% CEEC, and e compared with the untreated negative control. *P < 0.05; **P < 0.01; ***P < 0.001. Abbreviations: CEEC, crude extract of *Ehretia cymosa*; SO, simple ointment base (negative control); NF, nitrofurazone; LU, left untreated

Epithelialization in S. aureus-infected mice

The epithelialization period was shorter in the *E. cymosa* ointment preparation- and nitrofurazone-treated groups than in the negative control group. The epithelialization period for *S. aureus*-

infected mice decreased in a dose-dependent manner from 19.61±0.4 in the untreated negative control group to 17.2±0.37 in the 0.2% nitrofurazone ointment-treated group (Table 7). The 10% and 5% extract ointments reduced the epithelialization period in a dose-dependent manner (17.4% and 17.6%, respectively). Additionally, both the 5% and 10% (w/w) extract ointment groups presented significant (P < 0.05) differences in epithelialization time compared with the negative control group, with P<0.01 compared with the untreated (LU) groups.

Table 7: Effects of the 80% methanol extract of the leaves of *E. cymosa* on the epithelialization period in the wound-infected model

Groups	Epithelialization Period (Mean ± SEM), in Days	
	S. aureus	*P. aeruginosa.*
LU	19.60 ± 0.40	20.00 ± 0.00
SO	19.20 ± 0.37	19.80 ± 0.20
5%CEEC	17.60 ± 0.40^{a*e**}	19.40 ± 0.40
10%CEEC	17.40 ± 0.40 ^{a*e**}	18.80 ± 0.49^{e*}
0.2% NF	17.20 ± 0.37 ^{a**e**}	18.40 ± 0.49 ^{a**e***}

Note: Values are expressed as the mean ± SEM (n = 5); n = 5 mice in each group. Analysis was performed with one-way analysis of variance followed by Tukey's post hoc test: *a* compared with the negative control (SO), *b* compared with the positive control (NF), *c* compared with 5% CEEC, d compared with 10% CEEC, and e compared with LU. *P < 0.05; **P < 0.01; ***P < 0.001. Abbreviations: CEEC, crude extract of *Ehretia cymosa*; SO, simple ointment base (negative control); NF, nitrofurazone; LU, left untreated.

Epithelialization in P. aeruginosa-infected mice

The epithelialization period for *P. aeruginosa-infected* mice was reduced in a dose-related manner from 20.0 ± 0.00 for the untreated negative control to 18.40 ± 0.49 for the 0.2% nitrofurazone ointment-treated groups. The 10% extract ointment (18.80) and 5% extract ointment (19.40) reduced the period of epithelialization in a dose-dependent manner. Compared with the negative control group, the standard drug-treated group presented a faster rate of epithelialization (*P* < 0.01),

and $p < 0.001$ compared with the LU control group. Moreover, the 10% (w/w) extract ointment had a significant ($P < 0.05$) effect on the epithelialization period compared with the negative LU group. A significant ($p < 0.01$) difference in response to the 5% ointment formulation was observed in burn, followed by *P. aeruginosa*-infected mice. The same scenario was used for the 10% ointment preparation, with a significance level of $p < 0.01$ (Table 7).

Discussion

In the in vitro antibacterial activity tests, the 80% methanol extract of E. *cymosa* leaves resulted in zones of inhibition larger than 7 mm in diameter at all the tested concentrations (100, 200, and 300 mg/ml), indicating measurable antibacterial effects. The extract demonstrated notable effectiveness against *S. aureus*, *P. aeruginosa*, and *E. coli*. The in vivo model showed greater effectiveness against *S. aureus* than against *P. aeruginosa*, as evidenced by faster wound contraction and a shorter epithelialization period than that of the controls. These findings suggest that this plant is a promising candidate for further purification and investigation of its bioactive compounds for treating infectious diseases.

The results of the present study revealed that *S. aureus* was more susceptible than the other tested strains, with a 17 mm zone of inhibition at 100 mg/ml, followed by *P. aeruginosa* at 11 mm (Table 2). In a previous study conducted against four microbial isolates (*S. aureus, P. aeruginosa*, *E. coli*, and *B. subtilis*), the results indicated that the 70% ethanol extract of the whole plant material *E. cymosa* exhibited inhibitory activity and that *E. coli* was the most susceptible, with an MIC of 0.0108 µg/ml, whereas *P. aeruginosa* was the least susceptible, with an MIC of 0.1744 µg/ml (31). In another study, the antibacterial activity of *E. cymosa* was investigated against *S. aureus and P. aeruginosa* at a concentration of 25 mg/ml; the zones of inhibition observed by the ethyl acetate extract against *P. aeruginosa* and *S. aureus* were found to be 16 mm and 13 mm at 25 mg/ml,

respectively (32). This finding might support some of the uses reported by traditional healers in the treatment of wound infections. The antibacterial effect is important in the sole treatment of bacterial infections in humans.

E. cymose presented relatively high minimum inhibitory concentration (MIC) results across all standard bacterial strains tested. The MIC cutoff points ranged from 6.25 to 75 mg/ml; however, the minimum bactericidal concentration (MBC) value was 200 mg/ml against *E. coli* and *P. aeruginosa,* and no bactericidal activity was observed against *S. aureus, S. pyogenes, or K. pneumoniae* when the tested doses were considered. Knowledge of the MIC and MBC allowed the most appropriate choice of antimicrobial therapy to be made, decreasing the necessity of broad-spectrum antibiotics and decreasing the potential for the development of resistance (33, 34). The MBC/MIC ratio indicates whether an antibiotic is primarily bactericidal or bacteriostatic (34). A low ratio (typically ≤4) suggests a bactericidal effect, which is important for severe infections needing rapid bacterial elimination (35). A high ratio (>4) indicates a primarily bacteriostatic effect, where the antibiotic inhibits growth and relies on the host's immune system to clear the infection (36). In line with these findings, *E. cymose* leaf extract might be bactericidal for *E. coli* and bacteriostatic for the other tested strains. In addition, it appears to be most potent (MIC = 6.25 mg/ml) against *P. aeruginosa* but has a high MBC>300 mg/ml (Table 2). A very high MBC value compared with the MIC could also indicate potential toxicity, as a high concentration might be effective (37).

To further strengthen the antibacterial activity and its traditional claims, an in vivo model—burn followed by infection—was employed. An ointment containing a crude extract of *E. cymosa* leaves had a statistically significant effect on wound healing in mice infected with *S. aureus* and *P. aeruginosa*. The infiltration, blister development, and edema disappeared in all treated groups after

a shorter period of time than the negative controls (Figs 2 and 3). The extract ointment resulted in a faster wound contraction rate in the groups treated with 10% extract ointment than in those treated with 5% extract ointment and the negative control groups (Tables 5 and 6), as well as a shorter duration of epithelialization than the negative controls did (Table 7). In addition, the plant showed a better activity against *S. aureus* than against *P. aeruginosa,* supporting the in vitro susceptibility test results.

In this investigation, the antibacterial efficacy of the extract against common wound-infecting microorganisms was corroborated by faster wound healing. Research has shown that removing invading organisms from infected wounds provides a conducive environment for healing (38). As evidenced in this study, groups treated with simple ointment presented a slow rate of wound area contraction and did not re-epithelialize during the follow-up period. Infection is a major obstacle in wound healing, as it can disrupt the process and, in severe cases, lead to death (38-41).

Previous studies have reported the presence of flavonoids, phenols, and alkaloids as the main components in different solvents and parts of *E. cymosa* (31, 32, 42). Several studies have indicated that flavonoids, terpenoids, and tannins are secondary metabolites with high antibacterial potential (43), whereas saponins have little or no activity (44). Flavonoids and tannins have been shown to exhibit activity against *E. coli, P. aeruginosa, K. pneumoniae*, and *P. mirabilis* (44). In addition, flavonoids have been shown to inhibit various bacterial virulence factors, such as quorum-sensing signal receptors, enzymes, and toxins. These molecular effects are supported by in vitro studies demonstrating the inhibition of biofilm formation and bacterial attachment to host ligands (44). By optimizing these compounds through structural modifications (structure–activity relationships), it may be possible to create effective antimicrobial agents.

Conclusions

The present study demonstrated that the 80% methanol extract of *E. cymosa* leaves has significant antibacterial activity in both in vitro and in vivo models. The effect was concentration-dependent against the tested pathogens. It was more potent in gram-negative bacteria in an in vitro test. However, greater effectiveness against *S. aureus* than against *P. aeruginosa* was demonstrated by faster wound contraction and a shorter epithelialization period in the in vivo model. These findings support the traditional use of the plant for wound healing. Further pharmacological research and identification of the active compounds are recommended.

Acknowledgments

The authors would like to acknowledge the laboratory assistance of Haramaya University in the process of undertaking this study.

Abbreviations

ATCC= American Type Culture Collection, CEEC=Crude extract of *Ehretia cymose*, CFU=Colony-Forming Units, CONs=Coagulase Negative Staphylococci, DMSO =Di-Methyl Sulfoxide, LU- Left Untreated**,** MBC=Minimum Bactericidal Concentration, MHA=Muller Hinton Agar, MIC=Minimum inhibitory concentration, MMP=Matrix Metalloproteinase, NF-Nitrofurazone, OECD=Organization for Economic Cooperation and Development, PDGF=Platelet-Derived Growth Factor, ROS=Reactive Oxygen Species, SO=Simple Ointment, SPSS=Statistical Package for Social Science

Author contributions

All authors made a significant contribution to the work reported, that is, in the conception, study design, execution, acquisition of data, analysis, and interpretation, or all these areas; they took part

in drafting, revising, or critically reviewing the manuscript. All the authors have read and approved the final manuscript.

References

1. Cook MA, Wright GD. The past, present, and future of antibiotics. Science translational medicine. 2022;14(657):eabo7793.
2. Mohr KI. History of antibiotics research. How to overcome the antibiotic crisis: facts, challenges, technologies and future perspectives. 2016:237-72.
3. Roose-Amsaleg C, Laverman AM. Do antibiotics have environmental side-effects? Impact of synthetic antibiotics on biogeochemical processes. Environmental Science and Pollution Research. 2016;23:4000-12.
4. Singh R, Sripada L, Singh R. Side effects of antibiotics during bacterial infection: mitochondria, the main target in host cell. Mitochondrion. 2014;16:50-4.
5. AlSheikh HMA, Sultan I, Kumar V, Rather IA, Al-Sheikh H, Tasleem Jan A, et al. Plant-based phytochemicals as possible alternative to antibiotics in combating bacterial drug resistance. Antibiotics. 2020;9(8):480.
6. Dutt B, Kumar S, Garg K. Mapping of plant-based medicine research in China and India. Research Evaluation. 2009;18(1):51-9.
7. van Wyk AS, Prinsloo G. Health, safety and quality concerns of plant-based traditional medicines and herbal remedies. South African Journal of Botany. 2020;133:54-62.
8. Majeed M. Evidence-based medicinal plant products for the health care of world population. Annals of Phytomedicine. 2017;6(1):1-4.
9. Wijesekera R. Plant-derived medicines and their role in global health. The medicinal plant Industry: Routledge; 2017. p. 1-18.
10. Wanga L, Nyamboki DK. Medicinal Plants Used in the Management of Skin Disorders in Kenya: A Review. Pharmacognosy Reviews. 2023;17(33).
11. Christensen SB. Drugs That Changed Society: History and Current Status of the Early Antibiotics: Salvarsan, Sulfonamides, and β-Lactams. Molecules. 2021;26(19):6057. PubMed PMID: doi:10.3390/molecules26196057.
12. Albahri G, Badran A, Hijazi A, Daou A, Baydoun E, Nasser M, et al. The therapeutic wound healing bioactivities of various medicinal plants. Life. 2023;13(2):317.
13. Shedoeva A, Leavesley D, Upton Z, Fan C. Wound healing and the use of medicinal plants. Evidence-Based Complementary and Alternative Medicine. 2019;2019.
14. Comino-Sanz IM, López-Franco MD, Castro B, Pancorbo-Hidalgo PL. The role of antioxidants on wound healing: A review of the current evidence. Journal of Clinical Medicine. 2021;10(16):3558.
15. Woodward R. The organisation for economic co-operation and development (OECD): Routledge; 2009.
16. Council NR, Earth Do, Studies L, Research IfLA. Guidance for the description of animal research in scientific publications. 2011.
17. Pharmacopoeia B. Department of health and social security Scottish home and health department. Office of the British Pharmacopoeia Commission, UK. 1988;2:713.

18. Tafesse G, Mekonnen Y, Makonnen E, Majinda RR, Bojase-Moleta G, Yeboah SO. Antibacterial activity of crude extracts and pure compounds isolated from Vernonia galamensis leaves. African Journal of Pharmacy and Pharmacology. 2018;12(11):136-41.
19. Ismael HM. In Vitro Evaluation of Pomegranate (Punica granatum) Peel Extract against Candida krusei as an alternative agent to antifungal medicines. Zanco Journal of Pure and Applied Sciences. 2022;34(1):69-79.
20. Balouiri M, Sadiki M, Ibnsouda SK. Methods for in vitro evaluating antimicrobial activity: A review. Journal of pharmaceutical analysis. 2016;6(2):71-9.
21. Mostafa AA, Al-Askar AA, Almaary KS, Dawoud TM, Sholkamy EN, Bakri MM. Antimicrobial activity of some plant extracts against bacterial strains causing food poisoning diseases. Saudi journal of biological sciences. 2018;25(2):361-6.
22. Yemata G, DESTA B, Fetene M. In vitro antibacterial activity of traditionally used medicinal plants against Xanthomonas campestris pv. musacearum in Ethiopia. Biodiversitas Journal of Biological Diversity. 2019;20(2):555-61.
23. Hudzicki J, Jones J. Teaching Clinical Chemistry on a Shoestring Budget. Clinical Laboratory Science. 2016;29(2).
24. Andrews JM. Determination of minimum inhibitory concentrations. Journal of antimicrobial Chemotherapy. 2001;48(suppl_1):5-16.
25. Clinical, Institute LS. Performance standards for antimicrobial susceptibility testing. Clinical and Laboratory Standards Institute Wayne, PA; 2020.
26. Rodríguez-Melcón C, Alonso-Calleja C, García-Fernández C, Carballo J, Capita R. Minimum inhibitory concentration (MIC) and minimum bactericidal concentration (MBC) for twelve antimicrobials (biocides and antibiotics) in eight strains of Listeria monocytogenes. Biology. 2021;11(1):46.
27. Rowan MP, Cancio LC, Elster EA, Burmeister DM, Rose LF, Natesan S, et al. Burn wound healing and treatment: review and advancements. Crit Care. 2015;19:243. Epub 20150612. doi: 10.1186/s13054-015-0961-2. PubMed PMID: 26067660; PubMed Central PMCID: PMC4464872.
28. Menegat TA, Oliveira AFd, Majewski MGC, Blanes L, Juliano Y, Novo NF, et al. Experimental models of scald burns. A scope review. Acta Cirúrgica Brasileira. 2019;34:e201901007.
29. Wiggins-Dohlvik K, Tharakan B. A rat burn injury model for studying changes in microvascular permeability. Traumatic and Ischemic Injury: Methods and Protocols. 2018:93-100.
30. Gouma E, Simos Y, Verginadis I, Lykoudis E, Evangelou A, Karkabounas S. A simple procedure for estimation of total body surface area and determination of a new value of Meeh's constant in rats. Laboratory animals. 2012;46(1):40-5.
31. Sarkodie J, Squire S, Kretchy I, Domozoro C, Ahiagbe K, Twumasi M, et al. The antihyperglycemic, antioxidant and antimicrobial activities of Ehretia cymosa. Journal of Pharmacognosy and Phytochemistry. 2015;4(3):105-11.
32. Yadessa M, Hailemichael T, Aman D, Teshome A. Antibacterial Triterpenoid from the Leaves Extract of Ehretia Cymosa. Ethiopian Journal of Science and Sustainable Development. 2018;5(2):42-53.
33. Mogana R, Adhikari A, Tzar MN, Ramliza R, Wiart C. Antibacterial activities of the extracts, fractions and isolated compounds from Canarium patentinervium Miq. against bacterial clinical isolates. BMC Complementary Medicine and Therapies. 2020;20(1):55. doi: 10.1186/s12906-020-2837-5.

34. Abedini A, Colin M, Hubert J, Charpentier E, Angelis A, Bounasri H, et al. Abundant Extractable Metabolites from Temperate Tree Barks: The Specific Antimicrobial Activity of Prunus Avium Extracts. Antibiotics. 2020;9(3):111. PubMed PMID: doi:10.3390/antibiotics9030111.
35. Ishak A, Mazonakis N, Spernovasilis N, Akinosoglou K, Tsioutis C. Bactericidal versus bacteriostatic antibacterials: clinical significance, differences and synergistic potential in clinical practice. J Antimicrob Chemother. 2025;80(1):1-17. doi: 10.1093/jac/dkae380. PubMed PMID: 39471409; PubMed Central PMCID: PMC11695898.
36. Levison ME, Levison JH. Pharmacokinetics and pharmacodynamics of antibacterial agents. Infect Dis Clin North Am. 2009;23(4):791-815, vii. doi: 10.1016/j.idc.2009.06.008. PubMed PMID: 19909885; PubMed Central PMCID: PMC3675903.
37. Pankey GA, Sabath LD. Clinical Relevance of Bacteriostatic versus Bactericidal Mechanisms of Action in the Treatment of Gram-Positive Bacterial Infections. Clinical Infectious Diseases. 2004;38(6):864-70. doi: 10.1086/381972.
38. Morguette AEB, Bartolomeu-Gonçalves G, Andriani GM, Bertoncini GES, Castro IMd, Spoladori LFdA, et al. The Antibacterial and Wound Healing Properties of Natural Products: A Review on Plant Species with Therapeutic Potential against Staphylococcus aureus Wound Infections. Plants. 2023;12(11):2147.
39. Chah K, Eze C, Emuelosi C, Esimone C. Antibacterial and wound healing properties of methanolic extracts of some Nigerian medicinal plants. Journal of ethnopharmacology. 2006;104(1-2):164-7.
40. Breijyeh Z, Karaman R. Antibacterial activity of medicinal plants and their role in wound healing. Future Journal of Pharmaceutical Sciences. 2024;10(1):68.
41. Patel JD, Shrivastava AK, Kumar V. Evaluation of some medicinal plants used in traditional wound healing preparations for antibacterial property against some pathogenic bacteria. Journal of Clinical Immunology and Immunopathology Research. 2009;1(1):012-07.
42. Ashagrie G, Abebe A, Umer S. Analgesic and Anti-Inflammatory Activities of 80% Methanol Extract and Solvent Fractions of Ehretia cymosa Thonn (Boraginaceae) Leaves in Rodents. Journal of Experimental Pharmacology. 2023:63-79.
43. Chowdhury SK, Misra D, Mandal V. Medicinal Plant-Derived Antimicrobials' Fight Against Multidrug-Resistant Pathogens. Medicinal and Aromatic Plants: Healthcare and Industrial Applications. 2021:391-427.
44. Sisay M, Bussa N, Gashaw T, Mengistu G. Investigating in vitro antibacterial activities of medicinal plants having folkloric repute in Ethiopian traditional medicine. Journal of evidence-based integrative medicine. 2019;24:2515690X19886276.

Click here to access/download;Figure;Fig.1tiff.tif

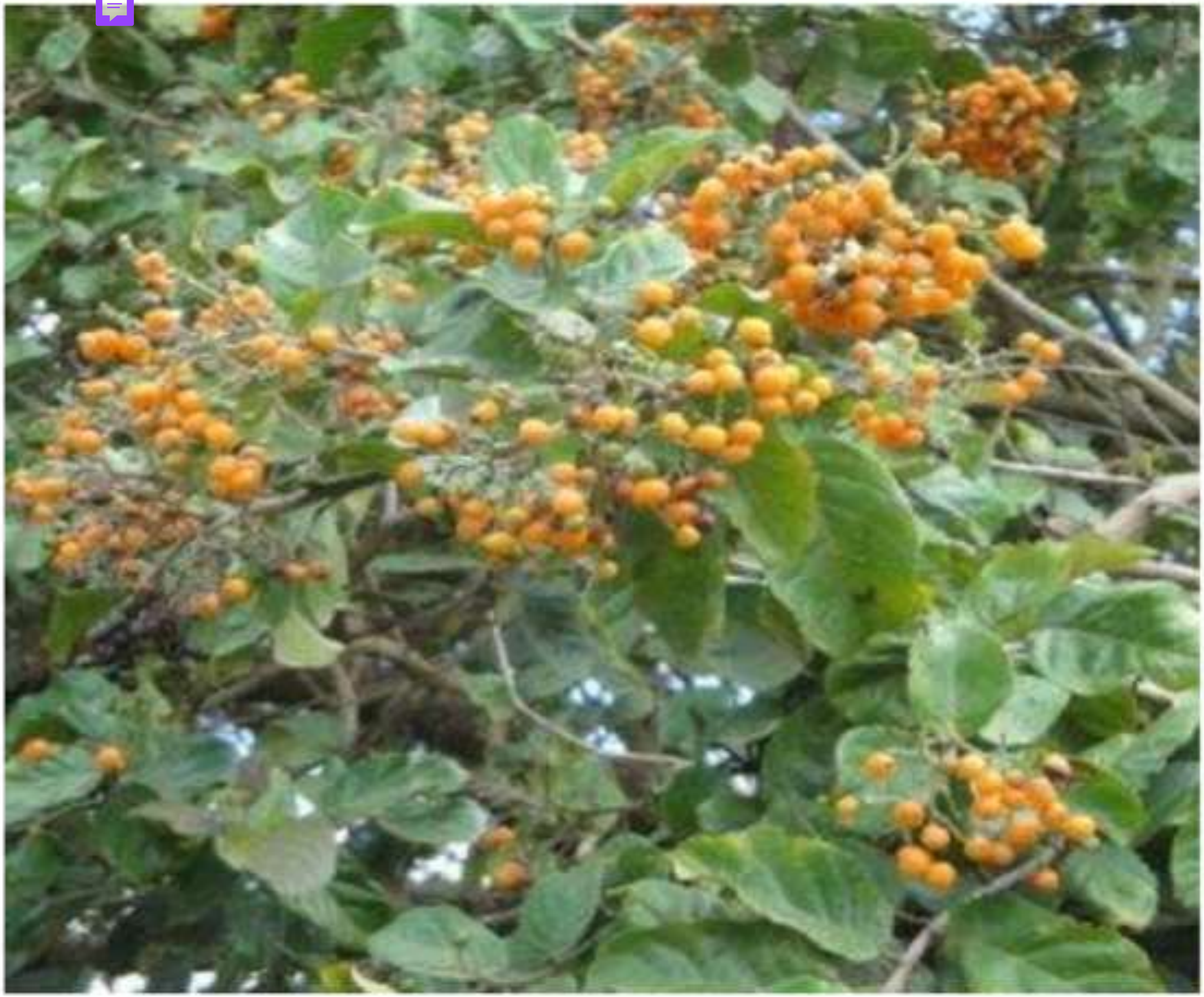

Click here to access/download;Figure;Fig.2 tiff.tif

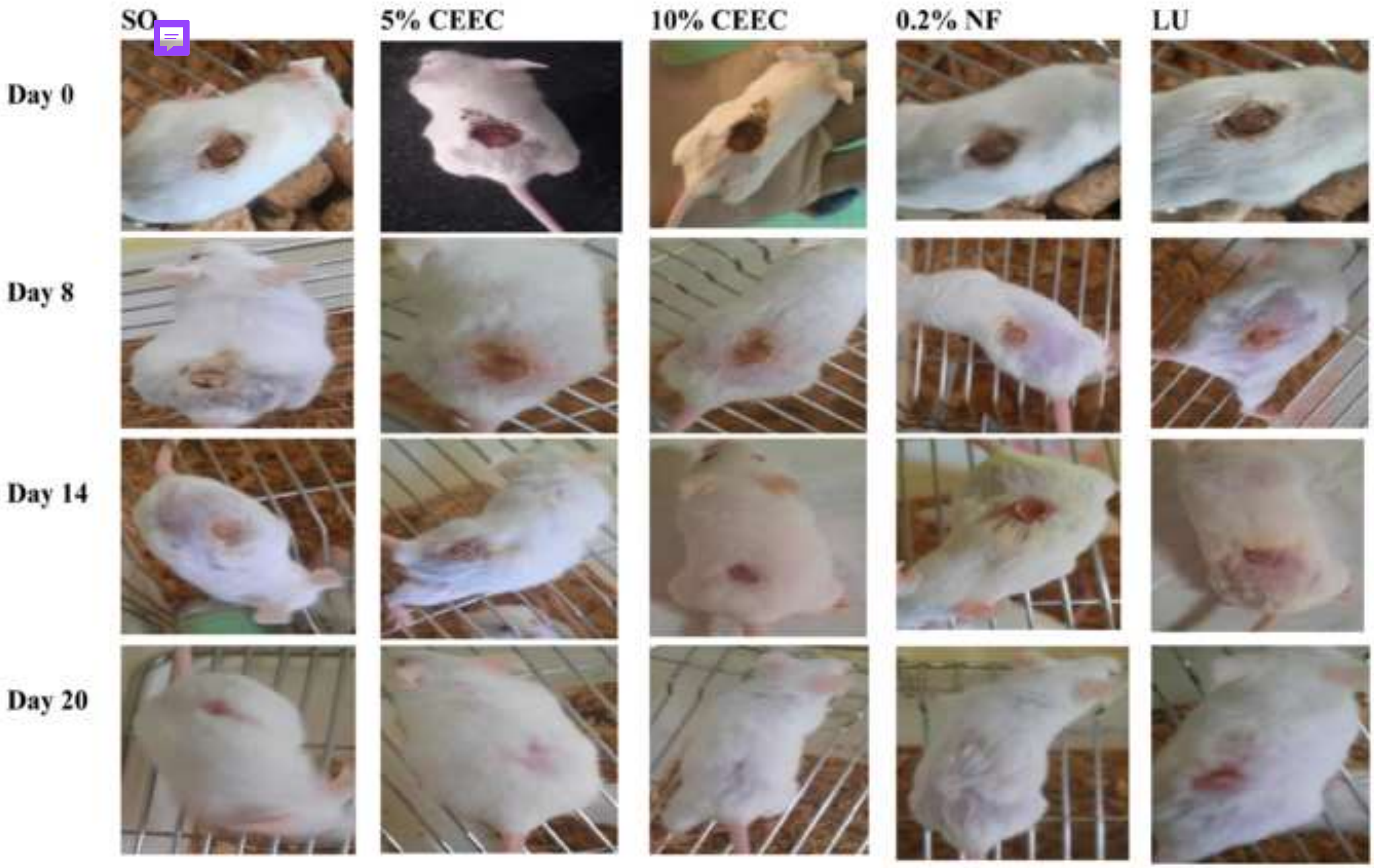

Click here to access/download;Figure;Fig.3 tiff.tif

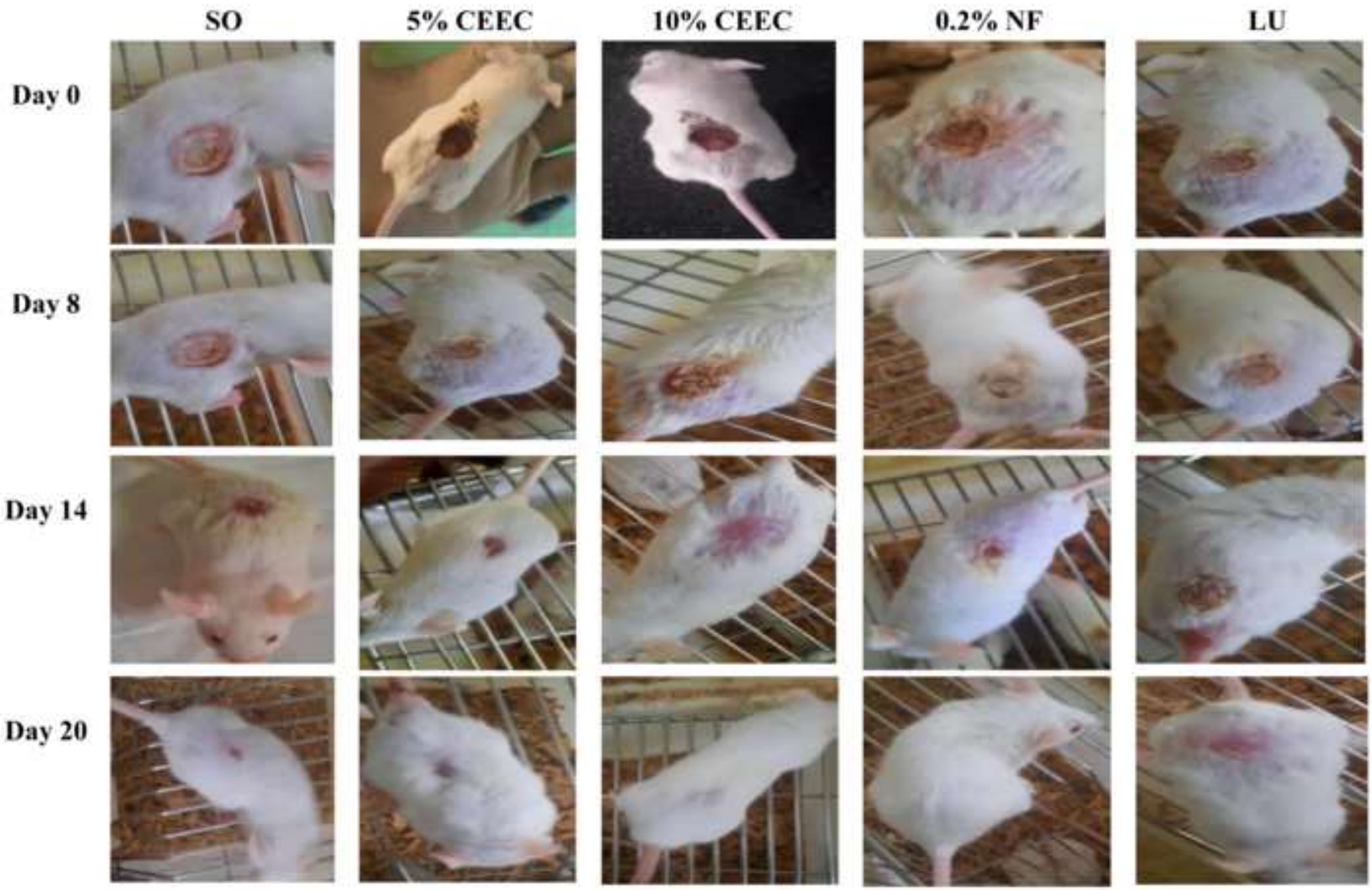

Supplement: Attachment — Submitted filename: Plose One comment.pdf [file pone.0354982.s007.pdf]
