# Supplementary material for: In vitro and in vivo antibacterial activities and phytochemical screening of 80% methanol extract from Ehretia cymosa leaves
Source: PLoS One. 2026 Jul 31;21(7):e0354982. doi: 10.1371/journal.pone.0354982 (PMC13427015; doi:10.1371/journal.pone.0354982)
Supplement: Attachment — Submitted filename: Review Report-Daniel.docx [file pone.0354982.s003.docx]

**Review Report**

**MS No.:**	PONE-D-25-54833

**Title:**	*In vitro* and *in vivo* antibacterial activities of 80% methanol crude *Ehretia cymosa* leaf extract (Boraginaceae)

**Date:**	Dec. 12, 2025

The manuscript investigates the antibacterial activity of *Ehretia cymosa* leaf extract through both *in vitro* assays and an *in vivo* infected-wound model, an important area of study given rising antimicrobial resistance and growing interest in medicinal plants. The work is generally well organized, provides adequate methodological detail, and has several strengths, including the combined use of laboratory and animal models, clear descriptions of plant processing and animal handling, and strong relevance to Ethiopian traditional medicine. However, substantial revisions are needed, particularly regarding experimental design, statistical analysis, clarity of writing, and interpretation of the findings, before the manuscript can be considered suitable for publication.

Thank you!

**I. Major Comments**

**1. Lack of Phytochemical Characterization**

The study uses a crude methanolic extract but does not include any qualitative or quantitative phytochemical analysis. This omission limits interpretation, as the antibacterial activity can vary greatly among different constituents, and it also makes comparison with previous studies difficult. I recommend that the authors include at least a preliminary phytochemical screening (e.g., phenolics, flavonoids, tannins, alkaloids, and saponins). Ideally, they should also report the total phenolic and flavonoid contents.

**2. Choice of Concentrations in Agar Diffusion**

The manuscript employs very high extract concentrations (100–300 mg/mL), which may lead to non-specific inhibition simply due to the large mass of extract used, and may also introduce diffusion limitations that complicate comparisons with ciprofloxacin. The authors should justify the need for such high concentrations and discuss the inherent limitations of comparing crude plant extracts with purified antibiotics.

**3. Missing MIC and MBC Clarity**

The reported MIC values are relatively high (6.25–75 mg/mL), and bactericidal activity is observed only for two organisms at very high concentrations (200 mg/mL). However, the manuscript does not compare these MIC values to standard interpretive criteria, nor does it explain the absence of MBC values for *S. aureus, S. pyogenes,* and *K. pneumoniae*. The authors should provide a clearer interpretation of whether these results indicate strong, moderate, or weak antibacterial activity

**4. Burn Model Description Needs Clarification**

The burn-creation procedure raises methodological concerns. The stated temperature of 100 °C is considerably lower than the typical 150–300 °C used in standardized rodent burn models, while the reported contact duration of 10 seconds is unusually long and may result in excessive tissue damage. The authors should provide appropriate citations supporting this method and justify the selected temperature and exposure time.

**5. Absence of Microbial Load Quantification**

The in vivo study lacks quantitative assessment of bacterial load before and after treatment, and it does not include microbiological confirmation that extract-treated wounds exhibit a reduced bacterial burden. This gap limits the ability to conclude whether the observed effects are truly antibacterial rather than solely wound-healing. The authors should include CFU quantification if feasible, or acknowledge this omission as a limitation.

**6. Randomization, Blinding, and Sample Size**

The manuscript does not clarify whether treatment group allocation was randomized, whether wound measurements were performed in a blinded manner, or how the chosen sample size (n = 5) was statistically justified. The authors should add statements addressing randomization, blinding, and any power estimation used to support the selected group size.

**7. Statistical Analysis Issues**

The tables use multiple superscript letters (a, b, c, d, e) that are not always clearly defined or applied consistently, and several results refer to “significant differences” without reporting the corresponding p-values. Moreover, effect sizes and confidence intervals are not provided. The authors should improve statistical transparency and refine the presentation of the results.

**8. Writing and Clarity Problems**

Several sections of the manuscript contain grammatical errors, repetition, and overly long sentences that affect clarity.

Examples (from Abstract and Introduction):

- “E. Cymose 80% methanol leaf extract” → *E. cymosa* (typo)
- “There is some evidence of its antibacterial activity…” but no citation support.
- In addition, many paragraphs in the Introduction are excessively long and combine unrelated ideas.

Comprehensive language editing is required throughout the manuscript.

**9. Interpretation and Overstatement**

The manuscript’s conclusion that the extract exhibits “significant antibacterial activity” appears overstated. The MIC values are very high, indicating low potency; several important pathogens showed no bactericidal response; and the observed in vivo improvements may be attributable to wound-healing effects rather than direct antibacterial action. The authors should revise the conclusions to present a more cautious and accurate interpretation of the findings.

**II. Minor Comments**

1. Include Latin binomial in italics throughout (*Ehretia cymosa*).
2. Clarify units (mg/ml vs mg/mL).
3. Avoid unnecessary repetition in Methods.
4. Replace low-quality figures with high-resolution ones.
5. Ensure all abbreviations are defined upon first use.

**Recommendation**

**Major Revision**

The study has scientific merit but requires substantial methodological, analytical, and editorial improvements.
