# Supplementary material for: In vitro and in vivo antibacterial activities and phytochemical screening of 80% methanol extract from Ehretia cymosa leaves
Source: PLoS One. 2026 Jul 31;21(7):e0354982. doi: 10.1371/journal.pone.0354982 (PMC13427015; doi:10.1371/journal.pone.0354982)
Supplement: Attachment — Submitted filename: Response to Reviewer.docx [file pone.0354982.s008.docx]

**MS No. PONE-D-25-54833**

**Title: *In vitro* and *in vivo* antibacterial activities of 80% methanol crude**

***Ehretia cymosa* leaf extract (Boraginaceae)**

**Dear Editor-in-Chief,**

Thank you for allowing us to edit our work. We appreciate the time and effort you invested in reading and evaluating our manuscript, as well as the insightful and helpful guidance you provided regarding our work. In response to the editor and reviewers' remarks, we revised the article and added clarifications to some of the suggestions and comments that required clarification. The highlighted modified document shows the changes made to the manuscript. In addition, we addressed each concern expressed by the editor and reviewers in detail. Under each suggestion, we addressed these explanations and justifications point by point. Please take the time to read our responses to the issues presented.

**Reviewer comment 1**

**Dear reviewer, thank you for all the insightful and constructive comments. The changes made in the manuscript are highlighted. Most of the comments have used for the manuscript's improvement.**

**# 1. The Introduction:** Add a short paragraph comparing your work to recent related studies (2020–2024) to emphasize relevance and current contribution.

**Response:** Dear reviewer, we have addressed this in the introduction sections. **Page 3**

**Extraction yield:** The manuscript describes the extraction method but does not report the yield (% w/w). Include this value for reproducibility.

**Response:** Dear reviewer, we have addressed this in the results sections. **Page 16**

“A total of 600g of course leaves powder of E cymosa extracted using 80% methanol, 104.17 g of dark brown paste obtained. The yield was calculated to be 17.36% (w/w). ‘’

**#2. Justification of doses:** Clarify why 5% and 10% ointment concentrations were selected and how they relate to MIC/MBC data.

**Response:** Dear reviewer, thank you for your insightful comment. We have included this justification in the method section,” **dosing in animal model**,” as follows

**In deciding the dose for in vivo administration, we tried to consider using a dose above MIC/MBC.** Since in vitro tests are performed under controlled conditions, it does not consider the range of physiological environments found in the animal body. In vivo dosing requires accounting for skin permeability, protein binding, and local metabolism to ensure the drug reaches the infection site in concentrations exceeding the MBC or maintains levels above the MIC to account for pharmacokinetic/pharmacodynamic factors. A common starting point, as proposed by the literature, was a concentration of **4x to 10x the MIC** in the target skin layer. But there were challenges: for example, the unit of measurement for the dose in the MIC/MBC determination is mg/mL (w/v), whereas we prepared an ointment (w/w). So, making direct dose calculations was challenging.

**Page 7**

**#3.** **Controls:** The use of ciprofloxacin and nitrofurazone is appropriate, but explicitly mention the rationale for their selection (e.g., broad-spectrum antibiotic and standard topical antibacterial agent).

**Response:** Dear reviewer, we have addressed this in the method section. **Page 10 & 12**

**#4.** **Sample size and power:** Include the number of replicates (n) for each assay and justify animal group sizes (e.g., n=5) according to statistical power or ethical guidelines.

**Response:** Dear reviewer, we have included how the animal group sizes were calculated according to ethical guidelines. We have used the “Resource Method” in exploratory and hypothesis-generating studies. This method is particularly useful when there’s limited or no prior information about the effect size or variability of the outcome in the study. Since this study was conducted on crude extract and in vivo for the first time, we used this general method. **Page 11**

**#5.** Ensure all p-values and post-hoc comparisons are clearly stated. Some tables (e.g., Tables 2, 5, 6) have multiple superscripts and comparisons that are difficult to interpret. Simplify with consistent notation and include legends explaining all comparisons.

**Response:** Thank you, we have made consistency, clarity, and explained the table. That was a helpful and supportive comment.

**#6.** Specify the software version and statistical tests (SPSS 27, one-way ANOVA with Tukey’s post-hoc) in the Methods section only once, avoiding repetition in Results.

**Response:** Dear reviewer, we have corrected as per your recommendation.

**#7.** Consider including effect sizes or confidence intervals where possible.

**Response:** Dear reviewer, we have presented the effect size in mean ± SD to appreciate the difference in the effect size across the groups. That is the most frequent presentation of the outcome in the literature, to focus on mean measurement with p-values for limiting overcrowding

**#8.** Figures 2 and 3 (burn wound healing images): Add clear labels (A–E) for each treatment group, include a scale bar, and improve resolution.

**Response:** Thank you, dear reviewer. We have added the label, used a scale bar, and the NAAS TOOL from PLOS for fitting the journal requirement, increasing resolutions

**#9.** Table formatting: Ensure uniform decimal places and consistent units (e.g., mg/mL).

**Response:** Dear reviewer, we have corrected as per your recommendation in each section and across the document.

**#10.**	MIC/MBC tables: Include reference antibiotic data for comparison (e.g., ciprofloxacin MICs).

**Response:** Dear reviewer, we have the standard comparator data for the zone of inhibition. Unfortunately, focusing on the plant extract, we were unable to retrieve the data on MIC/MBC for the comparator.

**#11.** **Graphical summary:** Adding a schematic diagram summarizing experimental design or main findings would greatly improve readability.

**Response:** Dear reviewer, we have added one document with a graphical summary or abstract. Please see figure 5

**#12.** The wound contraction results are promising, but interpretation should avoid overclaiming. Rephrase statements like “demonstrated greater effectiveness than controls” to “demonstrated significantly faster wound contraction compared to control groups.”

**Response:** Dear reviewer, we have corrected as per your recommendation in the discussion section. **Page 25**

**#13.** Discuss possible mechanisms (e.g., presence of flavonoids, phenolics) more critically and cite specific references linking these compounds to antibacterial and wound-healing effects.

**Response:** Dear reviewer, we have added paragraphs in the discussion sections with the proposed mechanism of secondary metabolites. Please refer to the revised manuscript. **Page 28**

**#14.** Provide a visual quantitative comparison (e.g., line graph of % wound contraction over time).

**Response:** Dear reviewer, we have added two bar graphs for infected wound models of *S. aureus* and *P. aeruginosa*. The line graph did not show the difference, so a bar graph was used to improve visual accuracy. Please see figures 3 and 4.

**#15.** Relating MIC/MBC data to previously published values.

**Response:** Dear reviewer, we have added to the discussion section. **Page** **26-27**

**#16.** Explaining why *S. aureus* was more susceptible than *P. aeruginosa* (e.g., cell wall differences).

**Response:** Dear reviewer, we have added to the discussion section. **Page 26**

*“S. aureus* generally appears more sensitive to some antibiotics than *P. aeruginosa* because *P.* *aeruginosa* has a tough outer membrane (Gram-negative structure) that blocks entry and uses efflux pumps to expel drugs, whereas *S. aureus* (Gram-positive) lacks this outer shield, making it inherently more accessible to antibiotics. “

**#17.** Adding a Limitations paragraph — e.g., crude extract used instead of purified compounds, lack of toxicity profiling, small sample size, absence of histopathology.

**Response:** Dear reviewer, we have added a limitation at the end of the discussion section and recommend the missing parameters for further investigations. **Page 29**

**Reviewer comment 2**

**#1-** The resolution of the figures should be adjusted, as it is very poor.

**Response:** Thank you, dear reviewer. We have added the label, used a scale bar, and the NAAS TOOL from PLOS for fitting the journal requirement, increasing resolutions

**#2-** The wound should be presented in a figure

**Response:** Dear reviewer, we have included a figure demonstrating the starting day and the date of significant progress made in figures 2 and 3.

**#3-** Where is the characterization of the prepared ointment?

**Response:** Dear reviewer, thank you for your important comment. The primary objective was focused on determining the bioactivities to generate scientific evidence and cross-check the traditional claim. And we just look at the physical properties of the ointment, such as a homogeneous consistency and smooth texture. Your comment is another insightful study area. We will take this biopharmaceutical recommendation in further studies of this plant.

**#4-** Where is the characterization of the plant extract?

**Response:** Dear reviewer, thank you for your important comment. We have included the qualitative phytochemical screening of the plant extract in method, result and discussion sections. Please visit the revised version of the manuscript.

**#5-** Numerous English errors in the manuscript should be revised.

**Response:** Thank you, we have accepted and revised the manuscript accordingly.

**#6-** The instruments section should be removed, and each instrument should be clarified in its position in the manuscript

**Response:** Thank you, we have accepted and revised the manuscript accordingly.

**#7-** A graphical abstract should be added

**Response:** Dear reviewer, we have added one document with a graphical summary or abstract. Please see figure 5

**#8-** More recent references should be cited

**Response:** Dear reviewer, we have accepted and revised as per your recommendation. However, some of the literature retained its originality.

**#9-** Where is the histological and immunohistochemical analysis of wound tissues? **Response:** Dear Reviewer, that is great insight. This study's objective focused on antibacterial activities. We will take the recommendations and conduct further studies on the wound healing activities of the plant, considering the remaining physiological parameters. Thankyou.

**Reviewer comment 3**

MS No.: PONE-D-25-54833

Title: In vitro and in vivo antibacterial activities of 80% methanol crude Ehretia cymosa leaf extract (Boraginaceae)

Date: Dec. 12, 2025

The manuscript investigates the antibacterial activity of Ehretia cymosa leaf extract through both in vitro assays and an in vivo infected-wound model, an important area of study given the rising antimicrobial resistance and growing interest in medicinal plants. The work is generally well organized, provides adequate methodological detail, and has several strengths, including the combined use of laboratory and animal models, clear descriptions of plant processing and animal handling, and strong relevance to Ethiopian traditional medicine. However, substantial revisions are needed, particularly regarding experimental design, statistical analysis, clarity of writing, and interpretation of the findings, before the manuscript can be considered suitable for publication.

Thank you!

**I. Major Comments**

**1. Lack of Phytochemical Characterization**

The study uses a crude methanolic extract but does not include any qualitative or quantitative phytochemical analysis. This omission limits interpretation, as the antibacterial activity can vary greatly among different constituents, and it also makes comparison with previous studies difficult. I recommend that the authors include at least a preliminary phytochemical screening (e.g., phenolics, flavonoids, tannins, alkaloids, and saponins). Ideally, they should also report the total phenolic and flavonoid contents.

**Response:** Dear reviewer, we have included the qualitative phytochemical screening and addressed the concern raised. Thank you for your insightful comment. Please visit the revised version of the manuscript.

**2. Choice of Concentrations in Agar Diffusion**

The manuscript employs very high extract concentrations (100–300 mg/mL), which may lead to non-specific inhibition simply due to the large mass of extract used, and may also introduce diffusion limitations that complicate comparisons with ciprofloxacin. The authors should justify the need for such high concentrations and discuss the inherent limitations of comparing crude plant extracts with purified antibiotics.

**Response:** Dear reviewer, you're right. Because of differences in chemical complexity, mechanisms of action, and standardization, it is difficult to compare the in vitro effects of a pure standard drug with those of a crude plant extract. Crude extracts contain complex combinations that can have both antagonistic (decreased) and synergistic (increased) effects, whereas pure drugs provide a predictable, unique result. We have addressed the justification and the limitations in the discussion, limitations, conclusion, and recommendation sections. **Pages 26, 28, 29**

**3. Missing MIC and MBC Clarity**

The reported MIC values are relatively high (6.25–75 mg/mL), and bactericidal activity is observed only for two organisms at very high concentrations (200 mg/mL). However, the manuscript does not compare these MIC values to standard interpretive criteria, nor does it explain the absence of MBC values for S. aureus, S. pyogenes, and K. pneumoniae. The authors should provide a clearer interpretation of whether these results indicate **strong, moderate, or weak antibacterial activity**

**Response:** Dear reviewer, Thank you for your insightful comment.

We have used the zone of inhibition to compare and classify the antibacterial activities using previous literature. However, to use the MIC, since it was a crude extract, we already tested at larger doses categorized as weak antibacterial activities (MIC Value: <0.5"mg/mL/μ"g/mL"- Highly potent: MIC Value: 0.5"mg/mL" to 1.5"mg/mL" Moderate potency, MIC Value: $>1.5\text{mg/mL}$ (often up to $10\text{mg/mL}$or higher) Weak/Low Antibacterial Activity. We placed both interpretations in the discussion section while elaborating the results. **Pages** 25-26

**4. Burn Model Description Needs Clarification**

The burn-creation procedure raises methodological concerns. The stated temperature of 100 °C is considerably lower than the typical 150–300 °C used in standardized rodent burn models, while the reported contact duration of 10 seconds is unusually long and may result in excessive tissue damage. The authors should provide appropriate citations supporting this method and justify the selected temperature and exposure time.

**Response:** Dear reviewer, Thank you for your insightful comment. We have a reference for using this procedure where the determination is based on animal type, total body surface burned, and time. 100 ^o^C and 8-12 seconds is acceptable. **Page 12**

**5. Absence of Microbial Load Quantification**

The in vivo study lacks a quantitative assessment of bacterial load before and after treatment, and it does not include microbiological confirmation that extract-treated wounds exhibit a reduced bacterial burden. This gap limits the ability to conclude whether the observed effects are truly antibacterial rather than solely wound-healing. The authors should include CFU quantification if feasible, or acknowledge this omission as a limitation.

**Response:** Dear reviewer, you're right. We have acknowledged this in the limitation sections. **Page 29**

**6. Randomization, Blinding, and Sample Size**

The manuscript does not clarify whether treatment group allocation was randomized, whether wound measurements were performed in a blinded manner, or how the chosen sample size (n = 5) was statistically justified. The authors should add statements addressing randomization, blinding, and any power estimation used to support the selected group size.

**Response:** Dear reviewer, thank you for your insightful comment. We have elaborated and addressed the comment in the method sections. **Page 11, 12, 13**

**7. Statistical Analysis Issues**

The tables use multiple superscript letters (a, b, c, d, e) that are not always clearly defined or applied consistently, and several results refer to “significant differences” without reporting the corresponding p-values. Moreover, effect sizes and confidence intervals are not provided. The authors should improve statistical transparency and refine the presentation of the results.

**Response:** Dear reviewer, we have revised the sections entirely to increase consistency and clarity in presenting and narrating the results. Please see the revised version.

**8. Writing and Clarity Problems**

Several sections of the manuscript contain grammatical errors, repetition, and overly long sentences that affect clarity. Examples (from Abstract and Introduction):

• “E. Cymose 80% methanol leaf extract” → E. cymosa (typo)

• “There is some evidence of its antibacterial activity…” but no citation support.

• In addition, many paragraphs in the Introduction are excessively long and combine unrelated ideas. Comprehensive language editing is required throughout the manuscript.

**Response:** Dear reviewer, we have revised all sections to increase clarity. Thank you, your comments have been a great input for improvements of the manuscript.

**9. Interpretation and Overstatement**

The manuscript’s conclusion that the extract exhibits “significant antibacterial activity” appears overstated. The MIC values are very high, indicating low potency; several important pathogens showed no bactericidal response; and the observed in vivo improvements may be attributable to wound-healing effects rather than direct antibacterial action. The authors should revise the conclusions to present a more cautious and accurate interpretation of the findings.

**Response:** Dear reviewer, we have revised the conclusion. **Page 29**

**II. Minor Comments**

1.	Include Latin binomial in italics throughout (Ehretia cymosa).

**Response:** Dear reviewer, we have revised accordingly.

2.	Clarify units (mg/ml vs mg/mL).

**Response:** Dear reviewer, we have made consistency.

3.	Avoid unnecessary repetition in Methods.

**Response:** Dear reviewer, we have revised accordingly.

4.	Replace low-quality figures with high-resolution ones.

**Response:** Dear reviewer, we have revised accordingly.

5.	Ensure all abbreviations are defined upon first use.

**Response:** Dear reviewer, we have revised accordingly.

**Recommendation**

Major Revision

The study has scientific merit but requires substantial methodological, analytical, and editorial improvements.

**Reviewer comment 4**

I was hardly following the narrations of tables in the results section. Narration was not well written. Please describe the main findings explicitly, and mention the significance level when comparing the tests.

**Response:** Dear reviewer, we have revised accordingly. Thank you, your comments have been a great input for improvements of the manuscript.
