# Supplementary material for: In vitro and in vivo antibacterial activities and phytochemical screening of 80% methanol extract from Ehretia cymosa leaves
Source: PLoS One. 2026 Jul 31;21(7):e0354982. doi: 10.1371/journal.pone.0354982 (PMC13427015; doi:10.1371/journal.pone.0354982)
Supplement: Attachment — Submitted filename: Review Report-PONE-D-25-54833_R1.docx [file pone.0354982.s006.docx]

**Reviewer Report for PONE-D-25-54833_R1**

The manuscript titled “ In vitro and in vivo antibacterial activities with phytochemical screening of 80% methanol crude *Ehretia cymosa* leaf extract (Boraginaceae)” investigates the biological activity and preliminary phytochemical screening of the leaf extract. The topic is scientifically relevant, particularly within the fields of natural product chemistry, pharmacology, and antimicrobial research.

While the study presents experimental work and some potentially useful findings, the manuscript requires **substantial revision** before it can meet the standards of clarity and scientific rigor expected for publication in PLoS One. Several aspects of the manuscript—including the clarity of the research objective, statistical analysis, presentation of results, and integration of findings with existing literature—require improvement.

**Comments**

**1. Title**

The title generally reflects the topic of the study; however, it could be improved for greater clarity. Therefore, the title may be revised as: “*In Vitro* and *In Vivo* Antibacterial Activities and Phytochemical Screening of 80% Methanol Extract of *Ehretia cymosa* Leaves.”

**2. Abstract**

The abstract provides a general overview of the study; however, it lacks sufficient clarity. In particular, the last two sentences should be revised and improved to enhance their clarity and readability: “The 80% methanol extract of *E. cymosa* leaves showed moderate antibacterial activity. These findings may partially support its traditional medicinal use and suggest that the plant could serve as a potential source of natural antimicrobial agents and other bioactive secondary metabolites. However, further studies are recommended, including the isolation and characterization of active compounds, microbiological investigations to quantify bacterial load, and pharmacological evaluation using wound-healing models.”

**3. Introduction**

The introduction provides some background information; however, it needs a clearer and stronger statement of the research gap. The manuscript states that “Experimental studies have shown that crude extracts and solvent fractions of *E. cymosa* exhibit a range of biological activities, including antioxidant, antihyperglycemic, antidiarrheal, paralysis-relieving, antiepileptic, and antimicrobial effects (11).” Since the antimicrobial activity of the plant has already been reported, the rationale for investigating its antibacterial activity again is not clearly justified. Therefore, the authors should explicitly explain the specific research gap and clarify how the present study differs from or adds to the existing knowledge.

**4. Materials and Methods**

- The Methods section has been significantly improved, but some sentences still lack clarity and could benefit from language refinement.

**5. Results**

- The statistical reporting in the tables (e.g., Tables 2 and 5) is quite complex due to the use of superscript letters and asterisks, which makes it difficult to understand. Please consider simplifying the presentation so that the results can be interpreted more easily.
- In Table 2, 5 μg (Cip) is reported. However, 5 μg represents only the amount, not the concentration. Please report Cip in terms of concentration instead.
- Tables 2 and 3 could be merged to improve clarity and the flow of data.
- In Tables 3 and 4, it is not necessary to report the test concentration since it has already been described in the Methods section.

**6. Discussion**

- The discussion interprets the results but is somewhat limited and does not adequately compare the findings with previous studies.
- In some sections, the discussion overinterprets the results, with conclusions appearing stronger than what the data actually support.

I therefore recommend: **Major Revision**
