# Supplementary material for: In vitro and in vivo antibacterial activities and phytochemical screening of 80% methanol extract from Ehretia cymosa leaves
Source: PLoS One. 2026 Jul 31;21(7):e0354982. doi: 10.1371/journal.pone.0354982 (PMC13427015; doi:10.1371/journal.pone.0354982)
Supplement: Attachment — Submitted filename: Response to Reviewer V3.docx [file pone.0354982.s009.docx]

**MS No. PONE-D-25-54833**

**Title: *In vitro* and *in vivo* antibacterial activities of 80% methanol crude**

***Ehretia cymosa* leaf extract**

**Dear Editor-in-Chief,**

Thank you for allowing us to edit our work. We appreciate the time and effort you invested in reading and evaluating our manuscript, as well as the insightful and helpful guidance you provided regarding our work. In response to the editor and reviewers' remarks, we revised the article and added clarifications to some of the suggestions and comments that required clarification. The highlighted modified document shows the changes made to the manuscript. In addition, we addressed each concern expressed by the editor and reviewers in detail. Under each suggestion, we addressed these explanations and justifications point by point. Please take the time to read our responses to the issues presented.

**Reviewer comment 1**

**Reviewer #1:** The authors have satisfactorily addressed the reviewers’ comments and revised the manuscript accordingly. The revisions have improved the clarity of the methodology, statistical presentation, figures, and discussion. The manuscript is now clearer, and the interpretation of the findings is more balanced. Overall, the study presents useful data on the antibacterial activity of *Ehretia cymosa* leaf extract.

**Response:** Dear reviewer, thank you for all the insightful and constructive comments.

**Reviewer comment 3**

**Reviewer #3:** Reviewer Report for PONE-D-25-54833_R1
The manuscript titled “In vitro and in vivo antibacterial activities with phytochemical screening of 80% methanol crude Ehretia cymosa leaf extract (Boraginaceae)” investigates the biological activity and preliminary phytochemical screening of the leaf extract. The topic is scientifically relevant, particularly within the fields of natural product chemistry, pharmacology, and antimicrobial research.
While the study presents experimental work and some potentially useful findings, the manuscript requires substantial revision before it can meet the standards of clarity and scientific rigor expected for publication in PLoS One. Several aspects of the manuscript—including the clarity of the research objective, statistical analysis, presentation of results, and integration of findings with existing literature—require improvement.

**Response:** Dear reviewer, thank you for all the insightful and constructive comments. The changes made in the manuscript are highlighted. Most of the comments have been used for the manuscript's improvement.

**# 1. Title**
The title generally reflects the topic of the study; however, it could be improved for greater clarity. Therefore, the title may be revised as: “In Vitro and In Vivo Antibacterial Activities and Phytochemical Screening of 80% Methanol Extract of Ehretia cymosa Leaves.”

**Response:** Dear reviewer, thank you. We have changed the title as per your recommendation.

**#2. Abstract**
The abstract provides a general overview of the study; however, it lacks sufficient clarity. In particular, the last two sentences should be revised and improved to enhance their clarity and readability: “The 80% methanol extract of E. cymosa leaves showed moderate antibacterial activity. These findings may partially support its traditional medicinal use and suggest that the plant could serve as a potential source of natural antimicrobial agents and other bioactive secondary metabolites. However, further studies are recommended, including the isolation and characterization of active compounds, microbiological investigations to quantify bacterial load, and pharmacological evaluation using wound-healing models.”

**Response:** Dear reviewer, we have rewritten the section. Thank you for your insightful comment. Please visit the revised version of the manuscript.

**3. Introduction**
The introduction provides some background information; however, it needs a clearer and stronger statement of the research gap. The manuscript states that “Experimental studies have shown that crude extracts and solvent fractions of E. cymosa exhibit a range of biological activities, including antioxidant, antihyperglycemic, antidiarrheal, paralysis-relieving, antiepileptic, and antimicrobial effects (11).” Since the plant's antimicrobial activity has already been reported, the rationale for investigating its antibacterial activity again is unclear. Therefore, the authors should explicitly explain the specific research gap and clarify how the present study differs from or adds to the existing knowledge.

**Response:** Dear reviewer, we have revised the introduction in the last section based on your recommendation. Thank you for your insightful comment. Please visit the revised version of the manuscript.

**4. Materials and Methods**
⎯ The Methods section has been significantly improved, but some sentences still lack clarity and could benefit from language refinement.

**Response:** Dear reviewer, we have made the edition as per your recommendation.

**5. Results**
⎯ The statistical reporting in the tables (e.g., Tables 2 and 5) is quite complex due to the use of superscript letters and asterisks, which makes it difficult to understand. Please consider simplifying the presentation so that the results can be interpreted more easily.

**Response:** Dear reviewer, we have tried to simplify by focusing on the most pertinent comparisons and results. However, the usual way of presenting the comparison groups and the level of significance in such a way like other literature.

⎯ In Table 2, 5 μg (Cip) is reported. However, 5 μg represents only the amount, not the concentration. Please report Cip in terms of concentration instead.

**Response:** Dear reviewer, we have revised as per your recommendation. Please visit the revised version of the manuscript.

⎯ Tables 2 and 3 could be merged to improve clarity and data flow.

**Response:** Dear reviewer, we have revised as per your recommendation.

⎯ In Tables 3 and 4, it is not necessary to report the test concentration since it has already been described in the Methods section.

**Response:** Dear reviewer, we have revised as per your recommendation.

**6. Discussion**
⎯ The discussion interprets the results but is somewhat limited and does not adequately compare the findings with previous studies.

**Response:** Dear reviewer, studies are limited on the E. cymosa plant activity; we have included previous results
⎯ In some sections, the discussion overinterprets the results, with conclusions appearing stronger than what the data actually support.

**Response:** Dear reviewer, we have revised as per your recommendation.

**I therefore recommend: Major Revision**

**Reviewer comment 4**

**#** The plant extract was previously evaluated for antibacterial activity, so this study appears to be a duplication of effort.

**Response:** Dear reviewer, we have revised as per your recommendation.

**#** Though there was a study conducted on antibacterial activity, we took a claim of the extract with the highest activity, the methanol extract, and tested for its activity in vitro and in vivo. We have also wanted to see the antibacterial activity in wound healing because it is the most traditional use of the plant in the study area. We are also further testing the plant for other wound healing models. Scientifically, it is recommended from the perspective of replicability and reproducibility as crucial pathways to attaining confidence in scientific knowledge, although not the only ones. Multiple channels of evidence from a variety of studies provide a robust means for gaining confidence in scientific knowledge over time.

**#** The narration of tables in the results section didn't follow standard English or scientific article writing standards.

**Response:** Dear reviewer, we have revised as per your recommendation.

**#** Additionally, the conclusion is not in line with the data presented in the manuscript.

**Response:** Dear reviewer, we have revised as per your recommendation.
