# Supplementary material for: In vitro and in vivo antibacterial activities and phytochemical screening of 80% methanol extract from Ehretia cymosa leaves
Source: PLoS One. 2026 Jul 31;21(7):e0354982. doi: 10.1371/journal.pone.0354982 (PMC13427015; doi:10.1371/journal.pone.0354982)
Supplement: Attachment — Submitted filename: Response to Reviewer V4.docx [file pone.0354982.s010.docx]

**MS No. PONE-D-25-54833 R3**

**Title: *In vitro and in vivo antibacterial activities and phytochemical screening of 80% methanol extract from Ehretia cymosa leaves.***

**Dear Editor-in-Chief,**

Thank you for allowing us to edit our work. We appreciate the time and effort you invested in reading and evaluating our manuscript, as well as the insightful and helpful guidance you provided regarding our work. In response to the editor and reviewers' remarks from the **attached PDF file**, we revised the article and added clarifications to some of the suggestions and comments that required clarification. The highlighted modified document shows the changes made to the manuscript. In addition, we addressed each concern expressed by the editor and reviewers in detail. Under each suggestion, we addressed these explanations and justifications **point by point**. Please take the time to read our responses to the issues presented.

**Reviewer comments**

**Abstract section:**

1. How would you determine the MIC and MBC? Difficult to do so; broth media were suitable for this purpose

**Response:** Dear reviewer, thank you for all the insightful and constructive comments. We have addressed it in the abstract and detailed it in the methods section under in vitro antibacterial testing. Pages 10- 11

**Introduction section:**

2. The demand for plant-based treatments is growing in both developed and developing countries, driven by the **perception** that these remedies are natural, safe, affordable, and generally associated with fewer side effects. Is it? If so, how did you conclude that drugs of natural origin are safe and less toxic?

**Response:** **Dear reviewer, you are right**. The community's practices over the years form the basis of our scientific investigations. We have revised the sentence as “The demand for plant-based treatments is growing in both developed and developing countries, driven by the **practice** that these remedies are natural, safe, affordable, and generally associated with fewer side effects.”

**Method section:**

3. Full description required for plant name in the headings or subheadings

**Response:** Dear reviewer, we have made the edits as per your recommendation.

4. Correcting Editorials and Abbreviations

**Response:** Dear reviewer, we have made the edits as per your recommendation.

5. In vitro antibacterial activity testing: on what basis have these bacteria been selected for evaluation?

**Response:** Dear reviewer, Common skin infectious/ wound infectious agents have been considered based on local and international literature

6. How do you sterilize sheep blood practically?

**Response:** Dear reviewer, we also revised accordingly.

In microbiology and research, sheep blood cannot be heat-sterilized, as high temperatures will destroy (hemolyze) red blood cells and coagulate proteins. Instead, it must be collected using strict aseptic techniques. Blood will be collected from a sheep in good health, without any antibiotic treatment or vaccine in the past 30 days. A trained veterinarian will collect the blood following aseptic technique into sterile EDTA tubes.

7. For antibacterial activities, the doses selected are too high to conclude whether the extract is active or inactive. How did you compare these doses with the dose of the positive control (ciprofloxacin is 5 micrograms)

**Response:** Dear reviewer, we justified and acknowledged the challenge in the discussion section. Page 26

Comparing the pharmacological activity of crude plant extracts to standard drugs is challenging due to the chemical variability of extracts versus the exact/predictable composition of a purified medication.

The fundamental challenges in this comparison include:

- **Complexity & Synergy:** Standard drugs use a single active compound, whereas crude extracts contain hundreds of diverse secondary metabolites. These compounds can cause **synergistic** or **antagonistic** interactions, making it difficult to tell which mechanism predominates to predict the dose
- **Batch-to-Batch Variability:** Extract composition fluctuates significantly based on environmental conditions, soil quality, harvesting time, and geographical location. Standard drugs maintain identical chemical profiles and exact concentrations across every batch.
- **Lack of Standardization:** Standard drugs have precisely defined dosages and thresholds. Because crude extracts have unknown concentrations of their active ingredients, calculating standard dose-responses and comparing efficacy can be unreliable.

Therefore, because we used a crude extract, we applied a general recommendation to calculate a safe sub-therapeutic dose (such as a starting dose in animal studies) based on acute toxicity, multiplying by a safety factor (usually 1/10 to 1/100). We considered one lower dose and another above the middle. Based on the results of the acute oral toxicity test, three dose levels were selected: the middle dose of the plant extract was one-tenth of the maximum dose determined in the acute oral toxicity study (200mg); the low dose of the plant extract was half of the middle dose (100mg); and the high dose of the plant extract could be twice the middle dose (400mg).

The in vivo dose must take into account skin permeability, protein binding, and local metabolism to ensure that the drug reaches the infection site. At concentrations more than the minimum bactericidal concentration (MBC), or maintains values above the minimum inhibitory concentration MIC while accounting for pharmacokinetic/

pharmacodynamic characteristics. According to studies, the target skin layer should begin at 4x to 10x MIC when determining a dose for in vivo delivery; thus, we planned to employ a dose greater than MIC or MBC obtained in vitro. Because in vitro tests are conducted under controlled conditions, they do not account for the wide range of physiological events that occur in the animal body.

8. How could you mix the polar extract with the very hydrophobic ointment base to make a uniform texture? Unless the researchers use surfactants, it is impossible to make a uniform-texture ointment. With a non-uniform-texture ointment, the wound area of mice could not be exposed to the extract. This could not generate reliable data.

**Response:** Dear reviewer, thank you for all the insightful and constructive comments. Table 1: Page 8

We have prepared the ointment based on the British Pharmacopoeia. These four ingredients are commonly combined to create the British Pharmacopoeia (B.P.) Simple Ointment is used as a base for medicated topical creams and ointments.

Together, they form an emollient, occlusive barrier that traps moisture in the skin. Their specific functions in the formulation are:

- **Wool Fat (Lanolin):** Acts as an emollient and natural emulsifier. It helps the base absorb water and promotes the penetration of active medications into the skin.

An emulsifier is actually a specific type of surfactant. While all emulsifiers are surfactants, not all surfactants are emulsifiers. The core difference lies in their primary function: surfactants are designed to clean and reduce surface tension, whereas emulsifiers are designed specifically to stabilize mixtures of oil and water. To successfully mix a polar extract with a hydrophobic ointment base, we used a **water-in-oil (w/o) emulsifier** (e.g., wool fat) to create a uniform, stable texture and avoid phase separation through **levigation /wetting**

- **Hard Paraffin:** Acts as a stiffening and hardening agent. It raises the melting point of the mixture so the ointment maintains a stable, usable consistency at room temperature.
- **Ceto-stearyl Alcohol:** Functions as a stabilizing and thickening agent. It improves the texture of the product, preventing the formula from separating or melting in warm weather.
- **White Soft Paraffin (Vaseline/Petrolatum):** Serves as the main carrier and a heavy occlusive emollient. It provides a greasy, protective layer that prevents moisture loss from the skin's surface.

9. Explain whether free diffusion of the extract and controls took place or not with respect to the polarity of the media, extract, and controls.

**Response:** Dear reviewer, thank you for all the insightful and constructive comments. We have revised the method section based on your comment. **Page 10-11**

**Diffusion Rate vs. Solubility:**

Agar diffusion effectiveness depends heavily on the physicochemical properties of the antimicrobial agent. Hydrophilic or low-molecular-weight compounds diffuse very efficiently through the agar gel, forming large zones of inhibition. However, these same compounds might lack the high chemical potency or concentration required to irreversibly kill the bacteria once they are suspended in liquid broth (where the MBC is evaluated).

In agar diffusion assays, **free diffusion depends strictly on matching polarities** between the medium, the extract, and the controls. Because agar is a highly polar, aqueous matrix, polar substances will diffuse freely, while non-polar or highly lipophilic compounds will fail to diffuse, leading to inconsistent or false-negative results.

Diffusion Analysis

**The Agar Media:** Agar is an aqueous (water-based) and hydrophilic gel, making it highly polar.

**The Extract:** If your plant or compound extract is **polar** (e.g., extracted with water or methanol), it will diffuse freely, creating a uniform concentration gradient. However, if the extract is **non-polar/lipophilic** (e.g., essential oils or hexane fractions), it will not dissolve well in the aqueous agar. Instead of diffusing radially into the gel, non-polar extracts tend to pool on the surface or evaporate, leading to irregular or no zones of inhibition.

**The Controls:**

- **Positive Controls:** Standard antibiotics are typically water-soluble (polar), allowing them to diffuse freely and radially through the agar.
- **Negative Controls:** Solvents used to dissolve the extract (DMSO) should share a similar polarity with the extract and agar to prevent precipitation and ensure the validity of the assay.

For valid comparisons in agar-diffusion testing, the extract, the solvent, the controls, and the agar must share comparable polarity.

10. This in vivo acute toxicity study could not be used as a test concentration basis for the in vitro study. Selectivity index is recommended for in vitro tests.

**Response:** Dear reviewer, we have made the edits as per your recommendation. We moved the sentence to the in vivo dose assumptions.

11. Visual analysis might not be accurate; it could be analyzed by inoculating the test tube without visible turbidity on solid media and observing bacterial growth

**Response:** Dear reviewer, we have made the edits as per your recommendation. **Page 11-12**

Yes, laboratory culturing is the fundamental step to determine both the **MIC** (Minimum Inhibitory Concentration) and **MBC** (Minimum Bactericidal Concentration). You cannot calculate these values without first growing the bacteria. Here is how the culture process is used to determine these values:

- **MIC Determination:** A standardized bacterial culture is grown in a liquid broth or on an agar plate containing varying, diluted concentrations of an antibiotic. The **MIC** is the lowest concentration that visibly *stops* the bacteria from growing (the lowest concentration that remains completely clear).
- **MBC Determination:** To find the **MBC**, scientists must subculture the samples from the clear MIC tubes/wells onto a new, drug-free agar plate. The **MBC** is the lowest antibiotic concentration that effectively *kills* ≥ 99.9% of the original bacterial population (meaning almost no colonies will grow on the new plate).

12. Researchers need to explain why these bacterial strains did not have an MBC, while the strongest activity was observed against *S. aureus* on the agar diffusion method

**Response:** Dear reviewer, we have revised the method section as per your recommendation. **Page 27-28**

The absence of a Minimum Bactericidal Concentration (MBC) alongside a strong zone of inhibition in an agar diffusion assay typically indicates that the tested agent is **bacteriostatic** rather than **bactericidal**.

The agar diffusion method primarily measures the **susceptibility** and **growth-inhibiting power** of an antimicrobial agent as it diffuses through the agar, but it does not differentiate between inhibiting bacterial growth and killing the bacteria.

Here are the specific scientific reasons researchers can use to explain this discrepancy:

- **Bacteriostatic vs. Bactericidal Activity:** The zone of inhibition confirms that the agent successfully halts the multiplication of *S. aureus*. However, to determine an MBC, the agent must kill 99.9% of the initial bacterial inoculum. When sub-cultured into fresh broth or onto agar without the antimicrobial agent, the bacteria will resurrect and regrow if the antibiotic merely momentarily inhibits growth, making it impossible to establish an MBC.
- **High Tolerance or "Persister" States:** In broth dilution assays used for MBC, *S. aureus* strains may exhibit a high tolerance or enter a dormant "persister" state. The agent may inhibit them at the Minimum Inhibitory Concentration (MIC) without completely eradicating them, requiring extremely high concentrations to achieve a bactericidal effect.
- **Diffusion Rate vs. Solubility:** Agar diffusion effectiveness depends heavily on the physicochemical properties of the antimicrobial agent. Hydrophilic or low-molecular-weight compounds diffuse very efficiently through the agar gel, forming large zones of inhibition. However, these same compounds might lack the high chemical potency or concentration required to irreversibly kill the bacteria once they are suspended in liquid broth (where the MBC is evaluated).
- **Alternative assays** to confirm whether the agent is primarily static or cidal
- **Methods to troubleshoot** solubility or agar-binding limitations in their MBC assays

**In the conclusion section:**

13. Revise and make an appropriate conclusion based on your findings (*S. aureus* is G+ve with higher activity)

**Response:** Dear reviewer, we have revised the method section as per your recommendation. **Page 30**
