# Supplementary material for: In vitro and in vivo antibacterial activities and phytochemical screening of 80% methanol extract from Ehretia cymosa leaves
Source: PLoS One. 2026 Jul 31;21(7):e0354982. doi: 10.1371/journal.pone.0354982 (PMC13427015; doi:10.1371/journal.pone.0354982)
Supplement: Peer review history

# Peer review: Decision Letter 0

**DOI:** 10.1371/journal.pone.0354982.r001

**Authors:** Jorddy Neves Cruz

22 Dec 2025

PONE-D-25-54833

In vitro and in vivo antibacterial activities of 80% methanol crude Ehretia cymosa leaf extract (Boraginaceae)

PLOS One

Dear Dr. Gashaw,

Thank you for submitting your manuscript to PLOS ONE. After careful consideration, we feel that it has merit but does not fully meet PLOS ONE’s publication criteria as it currently stands. Therefore, we invite you to submit a revised version of the manuscript that addresses the points raised during the review process.

Please submit your revised manuscript by Feb 05 2026 11:59PM. If you will need more time than this to complete your revisions, please reply to this message or contact the journal office at plosone@plos.org. When you're ready to submit your revision, log on to https://www.editorialmanager.com/pone/ and select the 'Submissions Needing Revision' folder to locate your manuscript file.

Please include the following items when submitting your revised manuscript:

- A letter that responds to each point raised by the academic editor and reviewer(s). You should upload this letter as a separate file labeled 'Response to Reviewers'.
- A marked-up copy of your manuscript that highlights changes made to the original version. You should upload this as a separate file labeled 'Revised Manuscript with Track Changes'.
- An unmarked version of your revised paper without tracked changes. You should upload this as a separate file labeled 'Manuscript'.

If you would like to make changes to your financial disclosure, please include your updated statement in your cover letter. Guidelines for resubmitting your figure files are available below the reviewer comments at the end of this letter.

If applicable, we recommend that you deposit your laboratory protocols in protocols.io to enhance the reproducibility of your results. Protocols.io assigns your protocol its own identifier (DOI) so that it can be cited independently in the future. For instructions see: https://journals.plos.org/plosone/s/submission-guidelines#loc-laboratory-protocols. Additionally, PLOS ONE offers an option for publishing peer-reviewed Lab Protocol articles, which describe protocols hosted on protocols.io. Read more information on sharing protocols at https://plos.org/protocols?utm_medium=editorial-email&utm_source=authorletters&utm_campaign=protocols.

We look forward to receiving your revised manuscript.

Kind regards,

Jorddy Neves Cruz

Academic Editor

PLOS One

Journal Requirements:

When submitting your revision, we need you to address these additional requirements.

1. Please ensure that your manuscript meets PLOS ONE's style requirements, including those for file naming. The PLOS ONE style templates can be found at

https://journals.plos.org/plosone/s/file?id=wjVg/PLOSOne_formatting_sample_main_body.pdf and https://journals.plos.org/plosone/s/file?id=ba62/PLOSOne_formatting_sample_title_authors_affiliations.pdf

2. In your Methods section, please provide additional information regarding the permits you obtained for the work. Please ensure you have included the full name of the authority that approved the field site access and, if no permits were required, a brief statement explaining why.

3. Thank you for stating the following financial disclosure:

[Haramaya University].

Please state what role the funders took in the study.  If the funders had no role, please state: “The funders had no role in study design, data collection and analysis, decision to publish, or preparation of the manuscript.”

If this statement is not correct you must amend it as needed.

Please include this amended Role of Funder statement in your cover letter; we will change the online submission form on your behalf.

4. We note that your Data Availability Statement is currently as follows: [All relevant data are within the manuscript and its Supporting Information files.]

Please confirm at this time whether or not your submission contains all raw data required to replicate the results of your study. Authors must share the “minimal data set” for their submission. PLOS defines the minimal data set to consist of the data required to replicate all study findings reported in the article, as well as related metadata and methods (https://journals.plos.org/plosone/s/data-availability#loc-minimal-data-set-definition).

For example, authors should submit the following data:

- The values behind the means, standard deviations and other measures reported;

- The values used to build graphs;

- The points extracted from images for analysis.

Authors do not need to submit their entire data set if only a portion of the data was used in the reported study.

If your submission does not contain these data, please either upload them as Supporting Information files or deposit them to a stable, public repository and provide us with the relevant URLs, DOIs, or accession numbers. For a list of recommended repositories, please see https://journals.plos.org/plosone/s/recommended-repositories.

If there are ethical or legal restrictions on sharing a de-identified data set, please explain them in detail (e.g., data contain potentially sensitive information, data are owned by a third-party organization, etc.) and who has imposed them (e.g., an ethics committee). Please also provide contact information for a data access committee, ethics committee, or other institutional body to which data requests may be sent. If data are owned by a third party, please indicate how others may request data access.

5. We note that Figure 1 in your submission may contain a copyrighted image. All PLOS content is published under the Creative Commons Attribution License (CC BY 4.0), which means that the manuscript, images, and Supporting Information files will be freely available online, and any third party is permitted to access, download, copy, distribute, and use these materials in any way, even commercially, with proper attribution. For more information, see our copyright guidelines: http://journals.plos.org/plosone/s/licenses-and-copyright.

We require you to either (1) present written permission from the copyright holder to publish these figures specifically under the CC BY 4.0 license, or (2) remove the figures from your submission:

1. You may seek permission from the original copyright holder of Figure 1 to publish the content specifically under the CC BY 4.0 license.

We recommend that you contact the original copyright holder with the Content Permission Form (http://journals.plos.org/plosone/s/file?id=7c09/content-permission-form.pdf) and the following text:

“I request permission for the open-access journal PLOS ONE to publish XXX under the Creative Commons Attribution License (CCAL) CC BY 4.0 (http://creativecommons.org/licenses/by/4.0/). Please be aware that this license allows unrestricted use and distribution, even commercially, by third parties. Please reply and provide explicit written permission to publish XXX under a CC BY license and complete the attached form.”

Please upload the completed Content Permission Form or other proof of granted permissions as an “Other” file with your submission.

In the figure caption of the copyrighted figure, please include the following text: “Reprinted from [ref] under a CC BY license, with permission from [name of publisher], original copyright [original copyright year].”

2. If you are unable to obtain permission from the original copyright holder to publish these figures under the CC BY 4.0 license or if the copyright holder’s requirements are incompatible with the CC BY 4.0 license, please either i) remove the figure or ii) supply a replacement figure that complies with the CC BY 4.0 license. Please check copyright information on all replacement figures and update the figure caption with source information. If applicable, please specify in the figure caption text when a figure is similar but not identical to the original image and is therefore for illustrative purposes only.

If the reviewer comments include a recommendation to cite specific previously published works, please review and evaluate these publications to determine whether they are relevant and should be cited. There is no requirement to cite these works unless the editor has indicated otherwise.

[Note: HTML markup is below. Please do not edit.]

Reviewers' comments:

Reviewer's Responses to Questions

**Comments to the Author**

1. Is the manuscript technically sound, and do the data support the conclusions?

The manuscript must describe a technically sound piece of scientific research with data that supports the conclusions. Experiments must have been conducted rigorously, with appropriate controls, replication, and sample sizes. The conclusions must be drawn appropriately based on the data presented.

Reviewer #1: Partly

Reviewer #2: Partly

Reviewer #3: Yes

Reviewer #4: Yes

**********

2. Has the statistical analysis been performed appropriately and rigorously?

Reviewer #1: Yes

Reviewer #2: Yes

Reviewer #3: No

Reviewer #4: Yes

**********

3. Have the authors made all data underlying the findings in their manuscript fully available?

The PLOS Data policy requires authors to make all data underlying the findings described in their manuscript fully available without restriction, with rare exception (please refer to the Data Availability Statement in the manuscript PDF file). The data should be provided as part of the manuscript or its supporting information, or deposited to a public repository. For example, in addition to summary statistics, the data points behind means, medians and variance measures should be available. If there are restrictions on publicly sharing data—e.g. participant privacy or use of data from a third party—those must be specified.

Reviewer #1: Yes

Reviewer #2: No

Reviewer #3: Yes

Reviewer #4: Yes

**********

4. Is the manuscript presented in an intelligible fashion and written in standard English?

PLOS ONE does not copyedit accepted manuscripts, so the language in submitted articles must be clear, correct, and unambiguous. Any typographical or grammatical errors should be corrected at revision, so please note any specific errors here.

Reviewer #1: Yes

Reviewer #2: No

Reviewer #3: No

Reviewer #4: No

**********

5. Review Comments to the Author

Please use the space provided to explain your answers to the questions above. You may also include additional comments for the author, including concerns about dual publication, research ethics, or publication ethics. (Please upload your review as an attachment if it exceeds 20,000 characters)

Reviewer #1: 1. The Introduction: Add a short paragraph comparing your work to recent related studies (2020–2024) to emphasize relevance and current contribution.

Extraction yield: The manuscript describes the extraction method but does not report the yield (% w/w). Include this value for reproducibility.

2. Justification of doses: Clarify why 5% and 10% ointment concentrations were selected and how they relate to MIC/MBC data.

3. Controls: The use of ciprofloxacin and nitrofurazone is appropriate but explicitly mention the rationale for their selection (e.g., broad-spectrum antibiotic and standard topical antibacterial agent).

4. Sample size and power: Include the number of replicates (n) for each assay and justify animal group sizes (e.g., n=5) according to statistical power or ethical guidelines.

5. Ensure all p-values and post-hoc comparisons are clearly stated. Some tables (e.g., Tables 2, 5, 6) have multiple superscripts and comparisons that are difficult to interpret. Simplify with consistent notation and include legends explaining all comparisons.

6. Specify the software version and statistical tests (SPSS 27, one-way ANOVA with Tukey’s post-hoc) in the Methods section only once, avoiding repetition in Results.

7. Consider including effect sizes or confidence intervals where possible.

8. Figures 2 and 3 (burn wound healing images): Add clear labels (A–E) for each treatment group, include a scale bar, and improve resolution.

9. Table formatting: Ensure uniform decimal places and consistent units (e.g., mg/mL).

10. MIC/MBC tables: Include reference antibiotic data for comparison (e.g., ciprofloxacin MICs).

11. Graphical summary: Adding a schematic diagram summarizing experimental design or main findings would greatly improve readability.

12. The wound contraction results are promising, but interpretation should avoid overclaiming. Rephrase statements like “demonstrated greater effectiveness than controls” to “demonstrated significantly faster wound contraction compared to control groups.”

13. Discuss possible mechanisms (e.g., presence of flavonoids, phenolics) more critically and cite specific references linking these compounds to antibacterial and wound-healing effects.

14. Provide visual quantitative comparison (e.g., line graph of % wound contraction over time).

15. Relating MIC/MBC data to previously published values.

16. Explaining why S. aureus was more susceptible than P. aeruginosa (e.g., cell wall differences).

17. Adding a Limitations paragraph — e.g., crude extract used instead of purified compounds, lack of toxicity profiling, small sample size, absence of histopathology.

Reviewer #2: Please consider the following comments:

1- The resolution of the figures should be adjusted as it is very poor.

2- The wound are should be presented in a figure

3- where is the characterization of the prepared ointment?

4- Where is the characterization of the plant extract?

5- There are numerous English errors in the manuscript that should be revised.

6- The instruments section should be removed and each instrument should be clarified in its position in the manuscript

7- A graphical abstract should be added

8- More recent references should be cited

9- Where is the histological and immunohistochemical analysis of wound tissues?

Reviewer #3: Review Report

MS No.: PONE-D-25-54833

Title: In vitro and in vivo antibacterial activities of 80% methanol crude Ehretia cymosa leaf extract (Boraginaceae)

Date: Dec. 12, 2025

The manuscript investigates the antibacterial activity of Ehretia cymosa leaf extract through both in vitro assays and an in vivo infected-wound model, an important area of study given rising antimicrobial resistance and growing interest in medicinal plants. The work is generally well organized, provides adequate methodological detail, and has several strengths, including the combined use of laboratory and animal models, clear descriptions of plant processing and animal handling, and strong relevance to Ethiopian traditional medicine. However, substantial revisions are needed, particularly regarding experimental design, statistical analysis, clarity of writing, and interpretation of the findings, before the manuscript can be considered suitable for publication.

Thank you!

________________________________________

I. Major Comments

1. Lack of Phytochemical Characterization

The study uses a crude methanolic extract but does not include any qualitative or quantitative phytochemical analysis. This omission limits interpretation, as the antibacterial activity can vary greatly among different constituents, and it also makes comparison with previous studies difficult. I recommend that the authors include at least a preliminary phytochemical screening (e.g., phenolics, flavonoids, tannins, alkaloids, and saponins). Ideally, they should also report the total phenolic and flavonoid contents.

2. Choice of Concentrations in Agar Diffusion

The manuscript employs very high extract concentrations (100–300 mg/mL), which may lead to non-specific inhibition simply due to the large mass of extract used, and may also introduce diffusion limitations that complicate comparisons with ciprofloxacin. The authors should justify the need for such high concentrations and discuss the inherent limitations of comparing crude plant extracts with purified antibiotics.

3. Missing MIC and MBC Clarity

The reported MIC values are relatively high (6.25–75 mg/mL), and bactericidal activity is observed only for two organisms at very high concentrations (200 mg/mL). However, the manuscript does not compare these MIC values to standard interpretive criteria, nor does it explain the absence of MBC values for S. aureus, S. pyogenes, and K. pneumoniae. The authors should provide a clearer interpretation of whether these results indicate strong, moderate, or weak antibacterial activity

4. Burn Model Description Needs Clarification

The burn-creation procedure raises methodological concerns. The stated temperature of 100 °C is considerably lower than the typical 150–300 °C used in standardized rodent burn models, while the reported contact duration of 10 seconds is unusually long and may result in excessive tissue damage. The authors should provide appropriate citations supporting this method and justify the selected temperature and exposure time.

5. Absence of Microbial Load Quantification

The in vivo study lacks quantitative assessment of bacterial load before and after treatment, and it does not include microbiological confirmation that extract-treated wounds exhibit a reduced bacterial burden. This gap limits the ability to conclude whether the observed effects are truly antibacterial rather than solely wound-healing. The authors should include CFU quantification if feasible, or acknowledge this omission as a limitation.

6. Randomization, Blinding, and Sample Size

The manuscript does not clarify whether treatment group allocation was randomized, whether wound measurements were performed in a blinded manner, or how the chosen sample size (n = 5) was statistically justified. The authors should add statements addressing randomization, blinding, and any power estimation used to support the selected group size.

7. Statistical Analysis Issues

The tables use multiple superscript letters (a, b, c, d, e) that are not always clearly defined or applied consistently, and several results refer to “significant differences” without reporting the corresponding p-values. Moreover, effect sizes and confidence intervals are not provided. The authors should improve statistical transparency and refine the presentation of the results.

8. Writing and Clarity Problems

Several sections of the manuscript contain grammatical errors, repetition, and overly long sentences that affect clarity.

Examples (from Abstract and Introduction):

• “E. Cymose 80% methanol leaf extract” → E. cymosa (typo)

• “There is some evidence of its antibacterial activity…” but no citation support.

• In addition, many paragraphs in the Introduction are excessively long and combine unrelated ideas.

Comprehensive language editing is required throughout the manuscript.

9. Interpretation and Overstatement

The manuscript’s conclusion that the extract exhibits “significant antibacterial activity” appears overstated. The MIC values are very high, indicating low potency; several important pathogens showed no bactericidal response; and the observed in vivo improvements may be attributable to wound-healing effects rather than direct antibacterial action. The authors should revise the conclusions to present a more cautious and accurate interpretation of the findings.

II. Minor Comments

1. Include Latin binomial in italics throughout (Ehretia cymosa).

2. Clarify units (mg/ml vs mg/mL).

3. Avoid unnecessary repetition in Methods.

4. Replace low-quality figures with high-resolution ones.

5. Ensure all abbreviations are defined upon first use.

Recommendation

Major Revision

The study has scientific merit but requires substantial methodological, analytical, and editorial improvements.

Reviewer #4: I was hardly following the narrations of tables in the results section. Narration was not well written. Please describe the main findings explicitly, and mention the significance level when comparing the tests.

**********

6. PLOS authors have the option to publish the peer review history of their article (what does this mean?). If published, this will include your full peer review and any attached files.

If you choose “no”, your identity will remain anonymous but your review may still be made public.

**Do you want your identity to be public for this peer review?**  For information about this choice, including consent withdrawal, please see our Privacy Policy.

Reviewer #1: No

Reviewer #2: No

Reviewer #3: No

Reviewer #4: No

**********

[NOTE: If reviewer comments were submitted as an attachment file, they will be attached to this email and accessible via the submission site. Please log into your account, locate the manuscript record, and check for the action link "View Attachments". If this link does not appear, there are no attachment files.]

To ensure your figures meet our technical requirements, please review our figure guidelines: https://journals.plos.org/plosone/s/figures

You may also use PLOS’s free figure tool, NAAS, to help you prepare publication quality figures: https://journals.plos.org/plosone/s/figures#loc-tools-for-figure-preparation.

NAAS will assess whether your figures meet our technical requirements by comparing each figure against our figure specifications.

Attachment Submitted filename: Review Report-Daniel.docx

Attachment Submitted filename: PONE-D-25-54833 comment.pdf

# Author comment: Author response to Decision Letter 1

**DOI:** 10.1371/journal.pone.0354982.r002

12 Feb 2026

MS No. PONE-D-25-54833

Title: In vitro and in vivo antibacterial activities of 80% methanol crude

Ehretia cymosa leaf extract (Boraginaceae)

Dear Editor-in-Chief,

Thank you for allowing us to edit our work. We appreciate the time and effort you invested in reading and evaluating our manuscript, as well as the insightful and helpful guidance you provided regarding our work. In response to the editor and reviewers' remarks, we revised the article and added clarifications to some of the suggestions and comments that required clarification. The highlighted modified document shows the changes made to the manuscript. In addition, we addressed each concern expressed by the editor and reviewers in detail. Under each suggestion, we addressed these explanations and justifications point by point. Please take the time to read our responses to the issues presented.

Reviewer comment 1

Dear reviewer, thank you for all the insightful and constructive comments. The changes made in the manuscript are highlighted. Most of the comments have used for the manuscript's improvement.

# 1. The Introduction: Add a short paragraph comparing your work to recent related studies (2020–2024) to emphasize relevance and current contribution.

Response: Dear reviewer, we have addressed this in the introduction sections. Page 3

Extraction yield: The manuscript describes the extraction method but does not report the yield (% w/w). Include this value for reproducibility.

Response: Dear reviewer, we have addressed this in the results sections. Page 16

“A total of 600g of course leaves powder of E cymosa extracted using 80% methanol, 104.17 g of dark brown paste obtained. The yield was calculated to be 17.36% (w/w). ‘’

#2. Justification of doses: Clarify why 5% and 10% ointment concentrations were selected and how they relate to MIC/MBC data.

Response: Dear reviewer, thank you for your insightful comment. We have included this justification in the method section,” dosing in animal model,” as follows

In deciding the dose for in vivo administration, we tried to consider using a dose above MIC/MBC. Since in vitro tests are performed under controlled conditions, it does not consider the range of physiological environments found in the animal body. In vivo dosing requires accounting for skin permeability, protein binding, and local metabolism to ensure the drug reaches the infection site in concentrations exceeding the MBC or maintains levels above the MIC to account for pharmacokinetic/pharmacodynamic factors. A common starting point, as proposed by the literature, was a concentration of 4x to 10x the MIC in the target skin layer. But there were challenges: for example, the unit of measurement for the dose in the MIC/MBC determination is mg/mL (w/v), whereas we prepared an ointment (w/w). So, making direct dose calculations was challenging.

Page 7

#3. Controls: The use of ciprofloxacin and nitrofurazone is appropriate, but explicitly mention the rationale for their selection (e.g., broad-spectrum antibiotic and standard topical antibacterial agent).

Response: Dear reviewer, we have addressed this in the method section. Page 10 & 12

#4. Sample size and power: Include the number of replicates (n) for each assay and justify animal group sizes (e.g., n=5) according to statistical power or ethical guidelines.

Response: Dear reviewer, we have included how the animal group sizes were calculated according to ethical guidelines. We have used the “Resource Method” in exploratory and hypothesis-generating studies. This method is particularly useful when there’s limited or no prior information about the effect size or variability of the outcome in the study. Since this study was conducted on crude extract and in vivo for the first time, we used this general method. Page 11

#5. Ensure all p-values and post-hoc comparisons are clearly stated. Some tables (e.g., Tables 2, 5, 6) have multiple superscripts and comparisons that are difficult to interpret. Simplify with consistent notation and include legends explaining all comparisons.

Response: Thank you, we have made consistency, clarity, and explained the table. That was a helpful and supportive comment.

#6. Specify the software version and statistical tests (SPSS 27, one-way ANOVA with Tukey’s post-hoc) in the Methods section only once, avoiding repetition in Results.

Response: Dear reviewer, we have corrected as per your recommendation.

#7. Consider including effect sizes or confidence intervals where possible.

Response: Dear reviewer, we have presented the effect size in mean ± SD to appreciate the difference in the effect size across the groups. That is the most frequent presentation of the outcome in the literature, to focus on mean measurement with p-values for limiting overcrowding

#8. Figures 2 and 3 (burn wound healing images): Add clear labels (A–E) for each treatment group, include a scale bar, and improve resolution.

Response: Thank you, dear reviewer. We have added the label, used a scale bar, and the NAAS TOOL from PLOS for fitting the journal requirement, increasing resolutions

#9. Table formatting: Ensure uniform decimal places and consistent units (e.g., mg/mL).

Response: Dear reviewer, we have corrected as per your recommendation in each section and across the document.

#10. MIC/MBC tables: Include reference antibiotic data for comparison (e.g., ciprofloxacin MICs).

Response: Dear reviewer, we have the standard comparator data for the zone of inhibition. Unfortunately, focusing on the plant extract, we were unable to retrieve the data on MIC/MBC for the comparator.

#11. Graphical summary: Adding a schematic diagram summarizing experimental design or main findings would greatly improve readability.

Response: Dear reviewer, we have added one document with a graphical summary or abstract. Please see figure 5

#12. The wound contraction results are promising, but interpretation should avoid overclaiming. Rephrase statements like “demonstrated greater effectiveness than controls” to “demonstrated significantly faster wound contraction compared to control groups.”

Response: Dear reviewer, we have corrected as per your recommendation in the discussion section. Page 25

#13. Discuss possible mechanisms (e.g., presence of flavonoids, phenolics) more critically and cite specific references linking these compounds to antibacterial and wound-healing effects.

Response: Dear reviewer, we have added paragraphs in the discussion sections with the proposed mechanism of secondary metabolites. Please refer to the revised manuscript. Page 28

#14. Provide a visual quantitative comparison (e.g., line graph of % wound contraction over time).

Response: Dear reviewer, we have added two bar graphs for infected wound models of S. aureus and P. aeruginosa. The line graph did not show the difference, so a bar graph was used to improve visual accuracy. Please see figures 3 and 4.

#15. Relating MIC/MBC data to previously published values.

Response: Dear reviewer, we have added to the discussion section. Page 26-27

#16. Explaining why S. aureus was more susceptible than P. aeruginosa (e.g., cell wall differences).

Response: Dear reviewer, we have added to the discussion section. Page 26

“S. aureus generally appears more sensitive to some antibiotics than P. aeruginosa because P. aeruginosa has a tough outer membrane (Gram-negative structure) that blocks entry and uses efflux pumps to expel drugs, whereas S. aureus (Gram-positive) lacks this outer shield, making it inherently more accessible to antibiotics. “

#17. Adding a Limitations paragraph — e.g., crude extract used instead of purified compounds, lack of toxicity profiling, small sample size, absence of histopathology.

Response: Dear reviewer, we have added a limitation at the end of the discussion section and recommend the missing parameters for further investigations. Page 29

Reviewer comment 2

#1- The resolution of the figures should be adjusted, as it is very poor.

Response: Thank you, dear reviewer. We have added the label, used a scale bar, and the NAAS TOOL from PLOS for fitting the journal requirement, increasing resolutions

#2- The wound should be presented in a figure

Response: Dear reviewer, we have included a figure demonstrating the starting day and the date of significant progress made in figures 2 and 3.

#3- Where is the characterization of the prepared ointment?

Response: Dear reviewer, thank you for your important comment. The primary objective was focused on determining the bioactivities to generate scientific evidence and cross-check the traditional claim. And we just look at the physical properties of the ointment, such as a homogeneous consistency and smooth texture. Your comment is another insightful study area. We will take this biopharmaceutical recommendation in further studies of this plant.

#4- Where is the characterization of the plant extract?

Response: Dear reviewer, thank you for your important comment. We have included the qualitative phytochemical screening of the plant extract in method, result and discussion sections. Please visit the revised version of the manuscript.

#5- Numerous English errors in the manuscript should be revised.

Response: Thank you, we have accepted and revised the manuscript accordingly.

#6- The instruments section should be removed, and each instrument should be clarified in its position in the manuscript

Response: Thank you, we have accepted and revised the manuscript accordingly.

#7- A graphical abstract should be added

Response: Dear reviewer, we have added one document with a graphical summary or abstract. Please see figure 5

#8- More recent references should be cited

Response: Dear reviewer, we have accepted and revised as per your recommendation. However, some of the literature retained its originality.

#9- Where is the histological and immunohistochemical analysis of wound tissues? Response: Dear Reviewer, that is great insight. This study's objective focused on antibacterial activities. We will take the recommendations and conduct further studies on the wound healing activities of the plant, considering the remaining physiological parameters. Thankyou.

Reviewer comment 3

MS No.: PONE-D-25-54833

Title: In vitro and in vivo antibacterial activities of 80% methanol crude Ehretia cymosa leaf extract (Boraginaceae)

Date: Dec. 12, 2025

The manuscript investigates the antibacterial activity of Ehretia cymosa leaf extract through both in vitro assays and an in vivo infected-wound model, an important area of study given the rising antimicrobial resistance and growing interest in medicinal plants. The work is generally well organized, provides adequate methodological detail, and has several strengths, including the combined use of laboratory and animal models, clear descriptions of plant processing and animal handling, and strong relevance to Ethiopian traditional medicine. However, substantial revisions are needed, particularly regarding experimental design, statistical analysis, clarity of writing, and interpretation of the findings, before the manuscript can be considered suitable for publication.

Thank you!

I. Major Comments

1. Lack of Phytochemical Characterization

The study uses a crude methanolic extract but does not include any qualitative or quantitative phytochemical analysis. This omission limits interpretation, as the antibacterial activity can vary greatly among different constituents, and it also makes comparison with previous studies difficult. I recommend that the authors include at least a preliminary phytochemical screening (e.g., phenolics, flavonoids, tannins, alkaloids, and saponins). Ideally, they should also report the total phenolic and flavonoid contents.

Response: Dear reviewer, we have included the qualitative phytochemical screening and addressed the concern raised. Thank you for your insightful comment. Please visit the revised version of the manuscript.

2. Choice of Concentrations in Agar Diffusion

The manuscript employs very high extract concentrations (100–300 mg/mL), which may lead to non-specific inhibition simply due to the large mass of extract used, and may also introduce diffusion limitations that complicate comparisons with ciprofloxacin. The authors should justify the need for such high concentrations and discuss the inherent limitations of comparing crude plant extracts with purified antibiotics.

Response: Dear reviewer, you're right. Because of differences in chemical complexity, mechanisms of action, and standardization, it is difficult to compare the in vitro effects of a pure standard drug with those of a crude plant extract. Crude extracts contain complex combinations that can have both antagonistic (decreased) and synergistic (increased) effects, whereas pure drugs provide a predictable, unique result. We have addressed the justification and the limitations in the discussion, limitations, conclusion, and recommendation sections. Pages 26, 28, 29

3. Missing MIC and MBC Clarity

The reported MIC values are relatively high (6.25–75 mg/mL), and bactericidal activity is observed only for two organisms at very high concentrations (200 mg/mL). However, the manuscript does not compare these MIC values to standard interpretive criteria, nor does it explain the absence of MBC values for S. aureus, S. pyogenes, and K. pneumoniae. The authors should provide a clearer interpretation of whether these results indicate strong, moderate, or weak antibacterial activity

Response: Dear reviewer, Thank you for your insightful comment.

We have used the zone of inhibition to compare and classify the antibacterial activities using previous literature. However, to use the MIC, since it was a crude extract, we already tested at larger doses categorized as weak antibacterial activities (MIC Value: <0.5"mg/mL/μ"g/mL"- Highly potent: MIC Value: 0.5"mg/mL" to 1.5"mg/mL" Moderate potency, MIC Value: >1.5"mg/mL" (often up to 10"mg/mL" or higher) Weak/Low Antibacterial Activity. We placed both interpretations in the discussion section while elaborating the results. Pages 25-26

4. Burn Model Description Needs Clarification

The burn-creation procedure raises methodological concerns. The stated temperature of 100 °C is considerably lower than the typical 150–300 °C used in standardized rodent burn models, while the reported contact duration of 10 seconds is unusually long and may result in excessive tissue damage. The authors should provide appropriate citations supporting this method and justify the selected temperature and exposure time.

Response: Dear reviewer, Thank you for your insightful comment. We have a reference for using this procedure where the determination is based on animal type, total body surface burned, and time. 100 oC and 8-12 seconds is acceptable. Page 12

5. Absence of Microbial Load Quantification

The in vivo study lacks a quantitative assessment of bacterial load before and after treatment, and it does not include microbiological confirmation that extract-treated wounds exhibit a reduced bacterial burden. This gap limits the ability to conclude whether the observed effects are truly antibacterial rather than solely wound-healing. The authors should include CFU quantification if feasible, or acknowledge this omission as a limitation.

Response: Dear reviewer, you're right. We have acknowledged this in the limitation sections. Page 29

6. Randomization, Blinding, and Sample Size

The manuscript does not clarify whether treatment group allocation was randomized, whether wound measurements were performed in a blinded manner, or how the chosen sample size (n = 5) was statistically justified. The authors should add statements addressing randomization, blinding, and any power estimation used to support the selected group size.

Response: Dear reviewer, thank you for your insightful comment. We have elaborated and addressed the comment in the method sections. Page 11, 12, 13

7. Statistical Analysis Issues

The tables use multiple superscript letters (a, b, c, d, e) that are not always clearly defined or applied consistently, and several results refer to “significant differences” without reporting the corresponding p-values. Moreover, effect sizes

Attachment Submitted filename: Response to Reviewer.docx

# Peer review: Decision Letter 1

**DOI:** 10.1371/journal.pone.0354982.r003

**Authors:** Jorddy Neves Cruz

29 Mar 2026

PONE-D-25-54833R1

In vitro and in vivo antibacterial activities with phytochemical screening of 80% methanol crude Ehretia cymosa leaf extract (Boraginaceae)

PLOS One

Dear Dr. Gashaw,

Thank you for submitting your manuscript to PLOS ONE. After careful consideration, we feel that it has merit but does not fully meet PLOS ONE’s publication criteria as it currently stands. Therefore, we invite you to submit a revised version of the manuscript that addresses the points raised during the review process.

==============================

Please submit your revised manuscript by May 13 2026 11:59PM. If you will need more time than this to complete your revisions, please reply to this message or contact the journal office at plosone@plos.org. When you're ready to submit your revision, log on to https://www.editorialmanager.com/pone/ and select the 'Submissions Needing Revision' folder to locate your manuscript file.

Please include the following items when submitting your revised manuscript:

- A letter that responds to each point raised by the academic editor and reviewer(s). You should upload this letter as a separate file labeled 'Response to Reviewers'.
- A marked-up copy of your manuscript that highlights changes made to the original version. You should upload this as a separate file labeled 'Revised Manuscript with Track Changes'.
- An unmarked version of your revised paper without tracked changes. You should upload this as a separate file labeled 'Manuscript'.

If you would like to make changes to your financial disclosure, please include your updated statement in your cover letter. Guidelines for resubmitting your figure files are available below the reviewer comments at the end of this letter.

If applicable, we recommend that you deposit your laboratory protocols in protocols.io to enhance the reproducibility of your results. Protocols.io assigns your protocol its own identifier (DOI) so that it can be cited independently in the future. For instructions see: https://journals.plos.org/plosone/s/submission-guidelines#loc-laboratory-protocols. Additionally, PLOS ONE offers an option for publishing peer-reviewed Lab Protocol articles, which describe protocols hosted on protocols.io. Read more information on sharing protocols at https://plos.org/protocols?utm_medium=editorial-email&utm_source=authorletters&utm_campaign=protocols.

As the corresponding author, your ORCID iD is verified in the submission system and will appear in the published article. PLOS supports the use of ORCID, and we encourage all coauthors to register for an ORCID iD and use it as well. Please encourage your coauthors to verify their ORCID iD within the submission system before final acceptance, as unverified ORCID iDs will not appear in the published article. *Only*  the individual author can complete the verification step; PLOS staff *cannot*  verify ORCID iDs on behalf of authors.

We look forward to receiving your revised manuscript.

Kind regards,

Jorddy Neves Cruz

Academic Editor

PLOS One

Journal Requirements:

If the reviewer comments include a recommendation to cite specific previously published works, please review and evaluate these publications to determine whether they are relevant and should be cited. There is no requirement to cite these works unless the editor has indicated otherwise.

[Note: HTML markup is below. Please do not edit.]

Reviewers' comments:

Reviewer's Responses to Questions

**Comments to the Author**

1. If the authors have adequately addressed your comments raised in a previous round of review and you feel that this manuscript is now acceptable for publication, you may indicate that here to bypass the “Comments to the Author” section, enter your conflict of interest statement in the “Confidential to Editor” section, and submit your "Accept" recommendation.

Reviewer #1: All comments have been addressed

Reviewer #3: All comments have been addressed

Reviewer #4: (No Response)

**********

2. Is the manuscript technically sound, and do the data support the conclusions?

The manuscript must describe a technically sound piece of scientific research with data that supports the conclusions. Experiments must have been conducted rigorously, with appropriate controls, replication, and sample sizes. The conclusions must be drawn appropriately based on the data presented.

Reviewer #1: Yes

Reviewer #3: Partly

Reviewer #4: Partly

**********

3. Has the statistical analysis been performed appropriately and rigorously?

Reviewer #1: Yes

Reviewer #3: No

Reviewer #4: Yes

**********

4. Have the authors made all data underlying the findings in their manuscript fully available?

The PLOS Data policy requires authors to make all data underlying the findings described in their manuscript fully available without restriction, with rare exception (please refer to the Data Availability Statement in the manuscript PDF file). The data should be provided as part of the manuscript or its supporting information, or deposited to a public repository. For example, in addition to summary statistics, the data points behind means, medians and variance measures should be available. If there are restrictions on publicly sharing data—e.g. participant privacy or use of data from a third party—those must be specified.

Reviewer #1: Yes

Reviewer #3: Yes

Reviewer #4: Yes

**********

5. Is the manuscript presented in an intelligible fashion and written in standard English?

PLOS ONE does not copyedit accepted manuscripts, so the language in submitted articles must be clear, correct, and unambiguous. Any typographical or grammatical errors should be corrected at revision, so please note any specific errors here.

Reviewer #1: Yes

Reviewer #3: No

Reviewer #4: No

**********

6. Review Comments to the Author

Please use the space provided to explain your answers to the questions above. You may also include additional comments for the author, including concerns about dual publication, research ethics, or publication ethics. (Please upload your review as an attachment if it exceeds 20,000 characters)

Reviewer #1: The authors have satisfactorily addressed the reviewers’ comments and revised the manuscript accordingly. The revisions have improved the clarity of the methodology, statistical presentation, figures, and discussion. The manuscript is now clearer and the interpretation of the findings is more balanced. Overall, the study presents useful data on the antibacterial activity of Ehretia cymosa leaf extract.

Reviewer #3: Reviewer Report for PONE-D-25-54833_R1

The manuscript titled “ In vitro and in vivo antibacterial activities with phytochemical screening of 80% methanol crude Ehretia cymosa leaf extract (Boraginaceae)” investigates the biological activity and preliminary phytochemical screening of the leaf extract. The topic is scientifically relevant, particularly within the fields of natural product chemistry, pharmacology, and antimicrobial research.

While the study presents experimental work and some potentially useful findings, the manuscript requires substantial revision before it can meet the standards of clarity and scientific rigor expected for publication in PLoS One. Several aspects of the manuscript—including the clarity of the research objective, statistical analysis, presentation of results, and integration of findings with existing literature—require improvement.

Comments

1. Title

The title generally reflects the topic of the study; however, it could be improved for greater clarity. Therefore, the title may be revised as: “In Vitro and In Vivo Antibacterial Activities and Phytochemical Screening of 80% Methanol Extract of Ehretia cymosa Leaves.”

2. Abstract

The abstract provides a general overview of the study; however, it lacks sufficient clarity. In particular, the last two sentences should be revised and improved to enhance their clarity and readability: “The 80% methanol extract of E. cymosa leaves showed moderate antibacterial activity. These findings may partially support its traditional medicinal use and suggest that the plant could serve as a potential source of natural antimicrobial agents and other bioactive secondary metabolites. However, further studies are recommended, including the isolation and characterization of active compounds, microbiological investigations to quantify bacterial load, and pharmacological evaluation using wound-healing models.”

3. Introduction

The introduction provides some background information; however, it needs a clearer and stronger statement of the research gap. The manuscript states that “Experimental studies have shown that crude extracts and solvent fractions of E. cymosa exhibit a range of biological activities, including antioxidant, antihyperglycemic, antidiarrheal, paralysis-relieving, antiepileptic, and antimicrobial effects (11).” Since the antimicrobial activity of the plant has already been reported, the rationale for investigating its antibacterial activity again is not clearly justified. Therefore, the authors should explicitly explain the specific research gap and clarify how the present study differs from or adds to the existing knowledge.

4. Materials and Methods

The Methods section has been significantly improved, but some sentences still lack clarity and could benefit from language refinement.

5. Results

The statistical reporting in the tables (e.g., Tables 2 and 5) is quite complex due to the use of superscript letters and asterisks, which makes it difficult to understand. Please consider simplifying the presentation so that the results can be interpreted more easily.

In Table 2, 5 μg (Cip) is reported. However, 5 μg represents only the amount, not the concentration. Please report Cip in terms of concentration instead.

Tables 2 and 3 could be merged to improve clarity and the flow of data.

In Tables 3 and 4, it is not necessary to report the test concentration since it has already been described in the Methods section.

6. Discussion

The discussion interprets the results but is somewhat limited and does not adequately compare the findings with previous studies.

In some sections, the discussion overinterprets the results, with conclusions appearing stronger than what the data actually support.

I therefore recommend: Major Revision

Reviewer #4: The plant extract was previously evaluated for antibacterial activity, so this study appears to be a duplication of effort. The narration of tables in the results section didn't follow standard English or scientific article writing standards. Additionally, the conclusion is not in line with the data presented in the manuscript.

**********

7. PLOS authors have the option to publish the peer review history of their article (what does this mean?). If published, this will include your full peer review and any attached files.

If you choose “no”, your identity will remain anonymous but your review may still be made public.

**Do you want your identity to be public for this peer review?**  For information about this choice, including consent withdrawal, please see our Privacy Policy.

Reviewer #1: **Yes:** Voravuth Somsak

Reviewer #3: No

Reviewer #4: **Yes:** Wubayehu Kahaliw (PhD, Associate Professor of Pharmacology), Department of Pharmacology, School of Pharmacy, College of Medicine and Health Sciences, University of Gondar.

**********

[NOTE: If reviewer comments were submitted as an attachment file, they will be attached to this email and accessible via the submission site. Please log into your account, locate the manuscript record, and check for the action link "View Attachments". If this link does not appear, there are no attachment files.]

To ensure your figures meet our technical requirements, please review our figure guidelines: https://journals.plos.org/plosone/s/figures

You may also use PLOS’s free figure tool, NAAS, to help you prepare publication quality figures: https://journals.plos.org/plosone/s/figures#loc-tools-for-figure-preparation.

NAAS will assess whether your figures meet our technical requirements by comparing each figure against our figure specifications.

Attachment Submitted filename: Review Report-PONE-D-25-54833_R1.docx

Attachment Submitted filename: Plose One comment.pdf

# Author comment: Author response to Decision Letter 2

**DOI:** 10.1371/journal.pone.0354982.r004

13 May 2026

MS No. PONE-D-25-54833

Title: In vitro and in vivo antibacterial activities of 80% methanol crude

Ehretia cymosa leaf extract

Dear Editor-in-Chief,

Thank you for allowing us to edit our work. We appreciate the time and effort you invested in reading and evaluating our manuscript, as well as the insightful and helpful guidance you provided regarding our work. In response to the editor and reviewers' remarks, we revised the article and added clarifications to some of the suggestions and comments that required clarification. The highlighted modified document shows the changes made to the manuscript. In addition, we addressed each concern expressed by the editor and reviewers in detail. Under each suggestion, we addressed these explanations and justifications point by point. Please take the time to read our responses to the issues presented.

Reviewer comment 1

Reviewer #1: The authors have satisfactorily addressed the reviewers’ comments and revised the manuscript accordingly. The revisions have improved the clarity of the methodology, statistical presentation, figures, and discussion. The manuscript is now clearer, and the interpretation of the findings is more balanced. Overall, the study presents useful data on the antibacterial activity of Ehretia cymosa leaf extract.

Response: Dear reviewer, thank you for all the insightful and constructive comments.

Reviewer comment 3

Reviewer #3: Reviewer Report for PONE-D-25-54833_R1

The manuscript titled “In vitro and in vivo antibacterial activities with phytochemical screening of 80% methanol crude Ehretia cymosa leaf extract (Boraginaceae)” investigates the biological activity and preliminary phytochemical screening of the leaf extract. The topic is scientifically relevant, particularly within the fields of natural product chemistry, pharmacology, and antimicrobial research.

While the study presents experimental work and some potentially useful findings, the manuscript requires substantial revision before it can meet the standards of clarity and scientific rigor expected for publication in PLoS One. Several aspects of the manuscript—including the clarity of the research objective, statistical analysis, presentation of results, and integration of findings with existing literature—require improvement.

Response: Dear reviewer, thank you for all the insightful and constructive comments. The changes made in the manuscript are highlighted. Most of the comments have been used for the manuscript's improvement.

# 1. Title

The title generally reflects the topic of the study; however, it could be improved for greater clarity. Therefore, the title may be revised as: “In Vitro and In Vivo Antibacterial Activities and Phytochemical Screening of 80% Methanol Extract of Ehretia cymosa Leaves.”

Response: Dear reviewer, thank you. We have changed the title as per your recommendation.

#2. Abstract

The abstract provides a general overview of the study; however, it lacks sufficient clarity. In particular, the last two sentences should be revised and improved to enhance their clarity and readability: “The 80% methanol extract of E. cymosa leaves showed moderate antibacterial activity. These findings may partially support its traditional medicinal use and suggest that the plant could serve as a potential source of natural antimicrobial agents and other bioactive secondary metabolites. However, further studies are recommended, including the isolation and characterization of active compounds, microbiological investigations to quantify bacterial load, and pharmacological evaluation using wound-healing models.”

Response: Dear reviewer, we have rewritten the section. Thank you for your insightful comment. Please visit the revised version of the manuscript.

3. Introduction

The introduction provides some background information; however, it needs a clearer and stronger statement of the research gap. The manuscript states that “Experimental studies have shown that crude extracts and solvent fractions of E. cymosa exhibit a range of biological activities, including antioxidant, antihyperglycemic, antidiarrheal, paralysis-relieving, antiepileptic, and antimicrobial effects (11).” Since the plant's antimicrobial activity has already been reported, the rationale for investigating its antibacterial activity again is unclear. Therefore, the authors should explicitly explain the specific research gap and clarify how the present study differs from or adds to the existing knowledge.

Response: Dear reviewer, we have revised the introduction in the last section based on your recommendation. Thank you for your insightful comment. Please visit the revised version of the manuscript.

4. Materials and Methods

The Methods section has been significantly improved, but some sentences still lack clarity and could benefit from language refinement.

Response: Dear reviewer, we have made the edition as per your recommendation.

5. Results

The statistical reporting in the tables (e.g., Tables 2 and 5) is quite complex due to the use of superscript letters and asterisks, which makes it difficult to understand. Please consider simplifying the presentation so that the results can be interpreted more easily.

Response: Dear reviewer, we have tried to simplify by focusing on the most pertinent comparisons and results. However, the usual way of presenting the comparison groups and the level of significance in such a way like other literature.

In Table 2, 5 μg (Cip) is reported. However, 5 μg represents only the amount, not the concentration. Please report Cip in terms of concentration instead.

Response: Dear reviewer, we have revised as per your recommendation. Please visit the revised version of the manuscript.

Tables 2 and 3 could be merged to improve clarity and data flow.

Response: Dear reviewer, we have revised as per your recommendation.

In Tables 3 and 4, it is not necessary to report the test concentration since it has already been described in the Methods section.

Response: Dear reviewer, we have revised as per your recommendation.

6. Discussion

The discussion interprets the results but is somewhat limited and does not adequately compare the findings with previous studies.

Response: Dear reviewer, studies are limited on the E. cymosa plant activity; we have included previous results

In some sections, the discussion overinterprets the results, with conclusions appearing stronger than what the data actually support.

Response: Dear reviewer, we have revised as per your recommendation.

I therefore recommend: Major Revision

Reviewer comment 4

# The plant extract was previously evaluated for antibacterial activity, so this study appears to be a duplication of effort.

Response: Dear reviewer, we have revised as per your recommendation.

# Though there was a study conducted on antibacterial activity, we took a claim of the extract with the highest activity, the methanol extract, and tested for its activity in vitro and in vivo. We have also wanted to see the antibacterial activity in wound healing because it is the most traditional use of the plant in the study area. We are also further testing the plant for other wound healing models. Scientifically, it is recommended from the perspective of replicability and reproducibility as crucial pathways to attaining confidence in scientific knowledge, although not the only ones. Multiple channels of evidence from a variety of studies provide a robust means for gaining confidence in scientific knowledge over time.

# The narration of tables in the results section didn't follow standard English or scientific article writing standards.

Response: Dear reviewer, we have revised as per your recommendation.

# Additionally, the conclusion is not in line with the data presented in the manuscript.

Response: Dear reviewer, we have revised as per your recommendation.

Attachment Submitted filename: Response to Reviewer V3.docx

# Editor report: Decision Letter 2

**DOI:** 10.1371/journal.pone.0354982.r005

**Authors:** Jorddy Neves Cruz

26 May 2026

PONE-D-25-54833R2

In Vitro and In Vivo Antibacterial Activities and Phytochemical Screening of 80% Methanol Extract from Ehretia cymosa Leaves

PLOS One

Dear Dr. Gashaw,

Thank you for submitting your manuscript to PLOS ONE. After careful consideration, we feel that it has merit but does not fully meet PLOS ONE’s publication criteria as it currently stands. Therefore, we invite you to submit a revised version of the manuscript that addresses the points raised during the review process.

**ACADEMIC EDITOR:**

- Dear Authors, In this new round of manuscript revision, please make all requested changes carefully and accurately..

-->Please submit your revised manuscript by Jul 10 2026 11:59PM. If you will need more time than this to complete your revisions, please reply to this message or contact the journal office at plosone@plos.org. When you're ready to submit your revision, log on to https://www.editorialmanager.com/pone/ and select the 'Submissions Needing Revision' folder to locate your manuscript file.

Please include the following items when submitting your revised manuscript:

- A letter that responds to each point raised by the academic editor and reviewer(s). You should upload this letter as a separate file labeled 'Response to Reviewers'.
- A marked-up copy of your manuscript that highlights changes made to the original version. You should upload this as a separate file labeled 'Revised Manuscript with Track Changes'.
- An unmarked version of your revised paper without tracked changes. You should upload this as a separate file labeled 'Manuscript'.

If you would like to make changes to your financial disclosure, please include your updated statement in your cover letter. Guidelines for resubmitting your figure files are available below the reviewer comments at the end of this letter.

If applicable, we recommend that you deposit your laboratory protocols in protocols.io to enhance the reproducibility of your results. Protocols.io assigns your protocol its own identifier (DOI) so that it can be cited independently in the future. For instructions see: https://journals.plos.org/plosone/s/submission-guidelines#loc-laboratory-protocols. Additionally, PLOS ONE offers an option for publishing peer-reviewed Lab Protocol articles, which describe protocols hosted on protocols.io. Read more information on sharing protocols at https://plos.org/protocols?utm_medium=editorial-email&utm_source=authorletters&utm_campaign=protocols.

As the corresponding author, your ORCID iD is verified in the submission system and will appear in the published article. PLOS supports the use of ORCID, and we encourage all coauthors to register for an ORCID iD and use it as well. Please encourage your coauthors to verify their ORCID iD within the submission system before final acceptance, as unverified ORCID iDs will not appear in the published article. *Only*  the individual author can complete the verification step; PLOS staff *cannot*  verify ORCID iDs on behalf of authors.

We look forward to receiving your revised manuscript.

Kind regards,

Jorddy Neves Cruz

Academic Editor

PLOS One

Journal Requirements:

If the reviewer comments include a recommendation to cite specific previously published works, please review and evaluate these publications to determine whether they are relevant and should be cited. There is no requirement to cite these works unless the editor has indicated otherwise.

[Note: HTML markup is below. Please do not edit.]

[NOTE: If reviewer comments were submitted as an attachment file, they will be attached to this email and accessible via the submission site. Please log into your account, locate the manuscript record, and check for the action link "View Attachments". If this link does not appear, there are no attachment files.]

To ensure your figures meet our technical requirements, please review our figure guidelines: https://journals.plos.org/plosone/s/figures

You may also use PLOS’s free figure tool, NAAS, to help you prepare publication quality figures: https://journals.plos.org/plosone/s/figures#loc-tools-for-figure-preparation.

NAAS will assess whether your figures meet our technical requirements by comparing each figure against our figure specifications.

# Author comment: Author response to Decision Letter 3

**DOI:** 10.1371/journal.pone.0354982.r006

2 Jul 2026

MS No. PONE-D-25-54833 R3

Title: In vitro and in vivo antibacterial activities and phytochemical screening of 80% methanol extract from Ehretia cymosa leaves.

Dear Editor-in-Chief,

Thank you for allowing us to edit our work. We appreciate the time and effort you invested in reading and evaluating our manuscript, as well as the insightful and helpful guidance you provided regarding our work. In response to the editor and reviewers' remarks from the attached PDF file, we revised the article and added clarifications to some of the suggestions and comments that required clarification. The highlighted modified document shows the changes made to the manuscript. In addition, we addressed each concern expressed by the editor and reviewers in detail. Under each suggestion, we addressed these explanations and justifications point by point. Please take the time to read our responses to the issues presented.

Reviewer comments

Abstract section:

1. How would you determine the MIC and MBC? Difficult to do so; broth media were suitable for this purpose

Response: Dear reviewer, thank you for all the insightful and constructive comments. We have addressed it in the abstract and detailed it in the methods section under in vitro antibacterial testing. Pages 10- 11

Introduction section:

2. The demand for plant-based treatments is growing in both developed and developing countries, driven by the perception that these remedies are natural, safe, affordable, and generally associated with fewer side effects. Is it? If so, how did you conclude that drugs of natural origin are safe and less toxic?

Response: Dear reviewer, you are right. The community's practices over the years form the basis of our scientific investigations. We have revised the sentence as “The demand for plant-based treatments is growing in both developed and developing countries, driven by the practice that these remedies are natural, safe, affordable, and generally associated with fewer side effects.”

Method section:

3. Full description required for plant name in the headings or subheadings

Response: Dear reviewer, we have made the edits as per your recommendation.

4. Correcting Editorials and Abbreviations

Response: Dear reviewer, we have made the edits as per your recommendation.

5. In vitro antibacterial activity testing: on what basis have these bacteria been selected for evaluation?

Response: Dear reviewer, Common skin infectious/ wound infectious agents have been considered based on local and international literature

6. How do you sterilize sheep blood practically?

Response: Dear reviewer, we also revised accordingly.

In microbiology and research, sheep blood cannot be heat-sterilized, as high temperatures will destroy (hemolyze) red blood cells and coagulate proteins. Instead, it must be collected using strict aseptic techniques. Blood will be collected from a sheep in good health, without any antibiotic treatment or vaccine in the past 30 days. A trained veterinarian will collect the blood following aseptic technique into sterile EDTA tubes.

7. For antibacterial activities, the doses selected are too high to conclude whether the extract is active or inactive. How did you compare these doses with the dose of the positive control (ciprofloxacin is 5 micrograms)

Response: Dear reviewer, we justified and acknowledged the challenge in the discussion section. Page 26

Comparing the pharmacological activity of crude plant extracts to standard drugs is challenging due to the chemical variability of extracts versus the exact/predictable composition of a purified medication.

The fundamental challenges in this comparison include:

• Complexity & Synergy: Standard drugs use a single active compound, whereas crude extracts contain hundreds of diverse secondary metabolites. These compounds can cause synergistic or antagonistic interactions, making it difficult to tell which mechanism predominates to predict the dose

• Batch-to-Batch Variability: Extract composition fluctuates significantly based on environmental conditions, soil quality, harvesting time, and geographical location. Standard drugs maintain identical chemical profiles and exact concentrations across every batch.

• Lack of Standardization: Standard drugs have precisely defined dosages and thresholds. Because crude extracts have unknown concentrations of their active ingredients, calculating standard dose-responses and comparing efficacy can be unreliable.

Therefore, because we used a crude extract, we applied a general recommendation to calculate a safe sub-therapeutic dose (such as a starting dose in animal studies) based on acute toxicity, multiplying by a safety factor (usually 1/10 to 1/100). We considered one lower dose and another above the middle. Based on the results of the acute oral toxicity test, three dose levels were selected: the middle dose of the plant extract was one-tenth of the maximum dose determined in the acute oral toxicity study (200mg); the low dose of the plant extract was half of the middle dose (100mg); and the high dose of the plant extract could be twice the middle dose (400mg).

The in vivo dose must take into account skin permeability, protein binding, and local metabolism to ensure that the drug reaches the infection site. At concentrations more than the minimum bactericidal concentration (MBC), or maintains values above the minimum inhibitory concentration MIC while accounting for pharmacokinetic/

pharmacodynamic characteristics. According to studies, the target skin layer should begin at 4x to 10x MIC when determining a dose for in vivo delivery; thus, we planned to employ a dose greater than MIC or MBC obtained in vitro. Because in vitro tests are conducted under controlled conditions, they do not account for the wide range of physiological events that occur in the animal body.

8. How could you mix the polar extract with the very hydrophobic ointment base to make a uniform texture? Unless the researchers use surfactants, it is impossible to make a uniform-texture ointment. With a non-uniform-texture ointment, the wound area of mice could not be exposed to the extract. This could not generate reliable data.

Response: Dear reviewer, thank you for all the insightful and constructive comments. Table 1: Page 8

We have prepared the ointment based on the British Pharmacopoeia. These four ingredients are commonly combined to create the British Pharmacopoeia (B.P.) Simple Ointment is used as a base for medicated topical creams and ointments.

Together, they form an emollient, occlusive barrier that traps moisture in the skin. Their specific functions in the formulation are:

• Wool Fat (Lanolin): Acts as an emollient and natural emulsifier. It helps the base absorb water and promotes the penetration of active medications into the skin.

An emulsifier is actually a specific type of surfactant. While all emulsifiers are surfactants, not all surfactants are emulsifiers. The core difference lies in their primary function: surfactants are designed to clean and reduce surface tension, whereas emulsifiers are designed specifically to stabilize mixtures of oil and water. To successfully mix a polar extract with a hydrophobic ointment base, we used a water-in-oil (w/o) emulsifier (e.g., wool fat) to create a uniform, stable texture and avoid phase separation through levigation /wetting

• Hard Paraffin: Acts as a stiffening and hardening agent. It raises the melting point of the mixture so the ointment maintains a stable, usable consistency at room temperature.

• Ceto-stearyl Alcohol: Functions as a stabilizing and thickening agent. It improves the texture of the product, preventing the formula from separating or melting in warm weather.

• White Soft Paraffin (Vaseline/Petrolatum): Serves as the main carrier and a heavy occlusive emollient. It provides a greasy, protective layer that prevents moisture loss from the skin's surface.

9. Explain whether free diffusion of the extract and controls took place or not with respect to the polarity of the media, extract, and controls.

Response: Dear reviewer, thank you for all the insightful and constructive comments. We have revised the method section based on your comment. Page 10-11

Diffusion Rate vs. Solubility:

Agar diffusion effectiveness depends heavily on the physicochemical properties of the antimicrobial agent. Hydrophilic or low-molecular-weight compounds diffuse very efficiently through the agar gel, forming large zones of inhibition. However, these same compounds might lack the high chemical potency or concentration required to irreversibly kill the bacteria once they are suspended in liquid broth (where the MBC is evaluated).

In agar diffusion assays, free diffusion depends strictly on matching polarities between the medium, the extract, and the controls. Because agar is a highly polar, aqueous matrix, polar substances will diffuse freely, while non-polar or highly lipophilic compounds will fail to diffuse, leading to inconsistent or false-negative results.

Diffusion Analysis

The Agar Media: Agar is an aqueous (water-based) and hydrophilic gel, making it highly polar.

The Extract: If your plant or compound extract is polar (e.g., extracted with water or methanol), it will diffuse freely, creating a uniform concentration gradient. However, if the extract is non-polar/lipophilic (e.g., essential oils or hexane fractions), it will not dissolve well in the aqueous agar. Instead of diffusing radially into the gel, non-polar extracts tend to pool on the surface or evaporate, leading to irregular or no zones of inhibition.

The Controls:

o Positive Controls: Standard antibiotics are typically water-soluble (polar), allowing them to diffuse freely and radially through the agar.

o Negative Controls: Solvents used to dissolve the extract (DMSO) should share a similar polarity with the extract and agar to prevent precipitation and ensure the validity of the assay.

For valid comparisons in agar-diffusion testing, the extract, the solvent, the controls, and the agar must share comparable polarity.

10. This in vivo acute toxicity study could not be used as a test concentration basis for the in vitro study. Selectivity index is recommended for in vitro tests.

Response: Dear reviewer, we have made the edits as per your recommendation. We moved the sentence to the in vivo dose assumptions.

11. Visual analysis might not be accurate; it could be analyzed by inoculating the test tube without visible turbidity on solid media and observing bacterial growth

Response: Dear reviewer, we have made the edits as per your recommendation. Page 11-12

Yes, laboratory culturing is the fundamental step to determine both the MIC (Minimum Inhibitory Concentration) and MBC (Minimum Bactericidal Concentration). You cannot calculate these values without first growing the bacteria. Here is how the culture process is used to determine these values:

• MIC Determination: A standardized bacterial culture is grown in a liquid broth or on an agar plate containing varying, diluted concentrations of an antibiotic. The MIC is the lowest concentration that visibly stops the bacteria from growing (the lowest concentration that remains completely clear).

• MBC Determination: To find the MBC, scientists must subculture the samples from the clear MIC tubes/wells onto a new, drug-free agar plate. The MBC is the lowest antibiotic concentration that effectively kills ≥ 99.9% of the original bacterial population (meaning almost no colonies will grow on the new plate).

12. Researchers need to explain why these bacterial strains did not have an MBC, while the strongest activity was observed against S. aureus on the agar diffusion method

Response: Dear reviewer, we have revised the method section as per your recommendation. Page 27-28

The absence of a Minimum Bactericidal Concentration (MBC) alongside a strong zone of inhibition in an agar diffusion assay typically indicates that the tested agent is bacteriostatic rather than bactericidal.

The agar diffusion method primarily measures the susceptibility and growth-inhibiting power of an antimicrobial agent as it diffuses through the agar, but it does not differentiate between inhibiting bacterial growth and killing the bacteria.

Here are the specific scientific reasons researchers can use to explain this discrepancy:

• Bacteriostatic vs. Bactericidal Activity: The zone of inhibition confirms that the agent successfully halts the multiplication of S. aureus. However, to determine an MBC, the agent must kill 99.9% of the initial bacterial inoculum. When sub-cultured into fresh broth or onto agar without the antimicrobial agent, the bacteria will resurrect and regrow if the antibiotic merely momentarily inhibits growth, making it impossible to establish an MBC.

• High Tolerance or "Persister" States: In broth dilution assays used for MBC, S. aureus strains may exhibit a high tolerance or enter a dormant "persister" state. The agent may inhibit them at the Minimum Inhibitory Concentration (MIC) without completely eradicating them, requiring extremely high concentrations to achieve a bactericidal effect.

• Diffusion Rate vs. Solubility: Agar diffusion effectiveness depends heavily on the physicochemical properties of the antimicrobial agent. Hydrophilic or low-molecular-weight compounds diffuse very efficiently through the agar gel, forming large zones of inhibition. However, these same compounds might lack the high chemical potency or concentration required to irreversibly kill the bacteria once they are suspended in liquid broth (where the MBC is evaluated).

• Alternative assays to confirm whether the agent is primarily static or cidal

• Methods to troubleshoot solubility or agar-binding limitations in their MBC assays

In the conclusion section:

13. Revise and make an appropriate conclusion based on your findings (S. aureus is G+ve with higher activity)

Response: Dear reviewer, we have revised the method section as per your recommendation. Page 30

Attachment Submitted filename: Response to Reviewer V4.docx

# Editor report: Decision Letter 3

**DOI:** 10.1371/journal.pone.0354982.r007

**Authors:** Jorddy Neves Cruz

16 Jul 2026

In vitro and in vivo antibacterial activities and phytochemical screening of 80% methanol extract from Ehretia cymosa leaves.

PONE-D-25-54833R3

Dear Dr. Gashaw,

We’re pleased to inform you that your manuscript has been judged scientifically suitable for publication and will be formally accepted for publication once it meets all outstanding technical requirements.

Within one week, you’ll receive an e-mail detailing the required amendments. When these have been addressed, you’ll receive a formal acceptance letter and your manuscript will be scheduled for publication.

An invoice will be generated when your article is formally accepted. Please note, if your institution has a publishing partnership with PLOS and your article meets the relevant criteria, all or part of your publication costs will be covered. Please make sure your user information is up-to-date by logging into Editorial Manager at Editorial Manager® and clicking the ‘Update My Information' link at the top of the page. For questions related to billing, please contact billing support.

If your institution or institutions have a press office, please notify them about your upcoming paper to help maximize its impact. If they’ll be preparing press materials, please inform our press team as soon as possible -- no later than 48 hours after receiving the formal acceptance. Your manuscript will remain under strict press embargo until 2 pm Eastern Time on the date of publication. For more information, please contact onepress@plos.org.

Kind regards,

Jorddy Neves Cruz

Academic Editor

PLOS One

Additional Editor Comments (optional):

Reviewers' comments:

# Editor report: Acceptance letter

**DOI:** 10.1371/journal.pone.0354982.r008

**Authors:** Jorddy Neves Cruz

PONE-D-25-54833R3

PLOS One

Dear Dr. Gashaw,

I'm pleased to inform you that your manuscript has been deemed suitable for publication in PLOS One. Congratulations! Your manuscript is now being handed over to our production team.

At this stage, our production department will prepare your paper for publication. This includes ensuring the following:

* All references, tables, and figures are properly cited

* All relevant supporting information is included in the manuscript submission,

* There are no issues that prevent the paper from being properly typeset

You will receive further instructions from the production team, including instructions on how to review your proof when it is ready. Please keep in mind that we are working through a large volume of accepted articles, so please give us a few days to review your paper and let you know the next and final steps.

Lastly, if your institution or institutions have a press office, please let them know about your upcoming paper now to help maximize its impact. If they'll be preparing press materials, please inform our press team within the next 48 hours. Your manuscript will remain under strict press embargo until 2 pm Eastern Time on the date of publication. For more information, please contact onepress@plos.org.

You will receive an invoice from PLOS for your publication fee after your manuscript has reached the completed accept phase. If you receive an email requesting payment before acceptance or for any other service, this may be a phishing scheme. Learn how to identify phishing emails and protect your accounts at https://explore.plos.org/phishing.

If we can help with anything else, please email us at customercare@plos.org.

Thank you for submitting your work to PLOS ONE and supporting open access.

Kind regards,

PLOS ONE Editorial Office Staff

on behalf of

Dr. Jorddy Neves Cruz

Academic Editor

PLOS One
